# From [2Mn2S] Diamond Cores to Butterfly Rhombs: Transformations That Highlight Alternating Peptide Binding Sites

**DOI:** 10.1021/acs.inorgchem.4c04051

**Published:** 2024-10-25

**Authors:** Trung H. Le, Kyle T. Burns, Manish Jana, Heather A. Arnold, Connor R. Vann, Marcetta Y. Darensbourg

**Affiliations:** Department of Chemistry, Texas A&M University, College Station, Texas 77845, United States

## Abstract

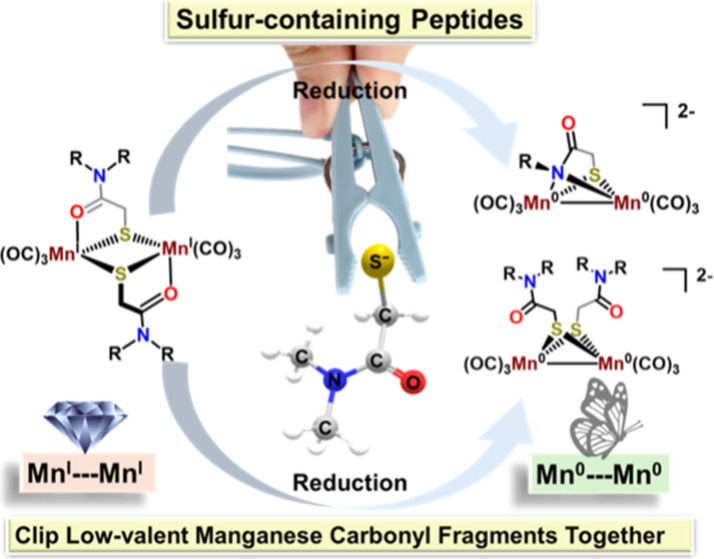

Thiocarboxamide chelates are known to assemble [2Mn2S] diamond core complexes via μ-S bridges that connect two Mn^I^(CO)_3_ fragments. These can exist as *syn-* and *anti*-isomers and interconvert via 16-electron, monomeric intermediates. Herein, we demonstrate that reduction of such Mn_2_ derivatives leads to a loss of one thiocarboxamide ligand and a switch of ligand binding mode from an O- to N-donor of the amide group, yielding a dianionic butterfly rhomb with a short Mn^0^–Mn^0^ distance, 2.52 Å. Structural and chemical analyses suggest that reduction of the Mn(I) centers is dependent on the protonation state of the amide-H, as total deprotonation followed by reduction does not result in the reduction of the Mn_2_ core. Partial deprotonation followed by reduction suggests a pathway that involves monomeric Mn(CO)_3_(S–O) and Mn(CO)_3_(S–N) intermediates. Ligand modifications to tertiary amides that remove the possibility of amide-H reduction led to complexes that preserve the [2Mn2S] diamond core during chemical reduction. Further comparison with the *tethered* system, linking the Mn(CO)_3_(S–O) sites together, suggests that dimer dissociation is necessary for the overall reductive transformation. These results highlight organomanganese carbonyl chemistry to establish illustrations of peptide fragment binding modes in the uptake of low-valent metal carbonyls related to binuclear active sites of biocatalysts.

## Abstract 2

Sulfur-containing peptide-like ligands clip manganese carbonyl fragments together in reduction-level-dependent structural forms.

## Introduction

A large research effort within the field of bioinorganic/bioorganometallic chemistry has addressed the assembly and function of a metalloenzyme family that catalyzes the production or utilization of dihydrogen (H_2_) known as hydrogenases. Iron is the natural metal of choice in both [FeFe]- and [NiFe]-H_2_ase, and biomimetic chemists are inclined to contrast ligands rather than metals to gain a better understanding of the action of the enzyme active sites. Hu et al. changed this paradigm and demonstrated that considerable information is to be gained from replacing the low-spin d^6^ Fe(II) center of the monoiron (or [Fe]-only) hydrogenase with Mn(I) (also d^6^ low-spin).^1−3^ Dimanganese carbonyls also offer comparisons to the diiron carbonyl biomimetics.^4−7^ The spectroscopic properties of manganese, when substituted for iron in ribonucleotide reductase (RNR), proved valuable to establish a model for the native diferrous form.^8^

**Scheme 1:**
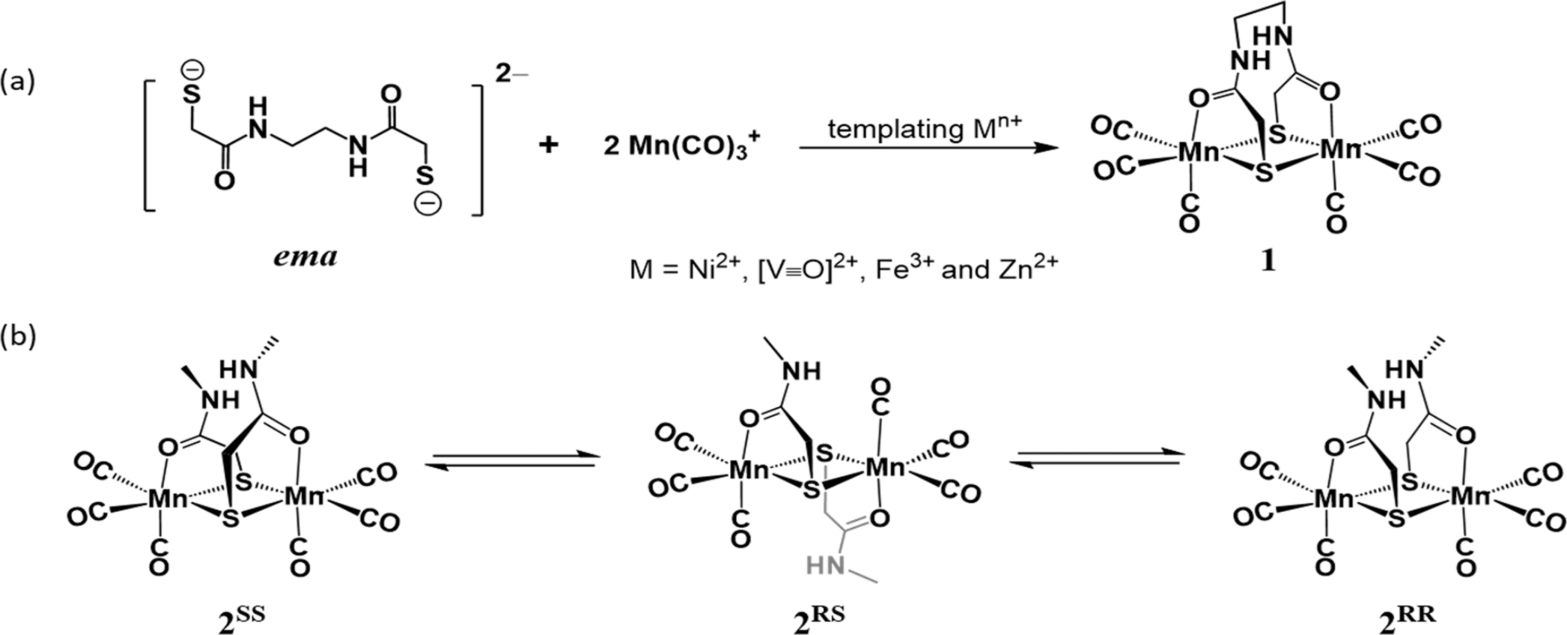
(a) Metal-Templated Assembly of **1**; (b) *Anti*/*Syn* Isomerization of **2**

While peptides as transition-metal carriers occupy a popular position in nutritional chemistry,^9−11^ systematic explorations in modern bioinorganic chemistry are lacking. Their attractive features as ligands lie in their versatility to accommodate, in concert with metal preferences, different redox and protonation levels. Our venture with a series of low-valent manganese carbonyl complexes featuring peptide-like ligands began with attempts to develop metallodithiolate ligands for another purpose, the construction of a S-bridged (MN_2_S_2_)·Mn(CO)_3_Br compound, using Holm’s *ema* ligand^12^ (*ema* = *N*,*N*′-ethylene-bis(mercaptoacetamide)) as a Cys-X-Cys biomimetic. While we expected that the sulfur-bridged bimetallic complex might have the potential for CO_2_ activation via M···Mn proximity,^13−25^ we found instead that the original tetradentate ligand ejected the metal from its N_2_S_2_ binding site, repositioned its chalcogen binding possibilities, and captured two Mn(CO)_3_^+^ moieties to form an octahedral, S-bridged, 18-e^–^ Mn^I^ complex, **Mn**_**2**_**ema**, compound **1**, Scheme 1.^26^ The synthesis of this *tethered* complex **1** was successful using several M^2+^ (Ni^2+^, [V≡O]^2+^, Fe^3+^, and especially Zn^2+^) as templating agents, to generate the κ^2^, S-bridging donor in the first coordination sphere of each Mn^I^, completed by a carboxamide oxygen. Further exploration of the interaction between amide and Mn^I^ within the diamond [2Mn2S] framework of complex **1** focused on the isomerization between the *anti* and *syn* isomers of the *nontethered* compound **2**, **[MnHE]**_**2**_ (HE = half-*ema*), Scheme 1b.^27^ Kinetic analysis, coupled with scrambling experiments,^27^ suggested that the isomers interconvert through an intermolecular pathway that involves 16-electron monomeric intermediates.

Note that the tethered thiocarboxamide is related to a pincer-type tridentate ligand, the pyridine bis-carboxamide, which is capable of stabilizing metals in higher oxidation states; it has found extensive application by Holm, Tolman, and others.^28−37^ The thiocarboxamide ligands in our studies are more flexible and in all cases use the thiolate-S to bridge two metals. The nearby N donors complete the mixed hard/soft first coordination sphere of the manganese.

**Scheme 2:**
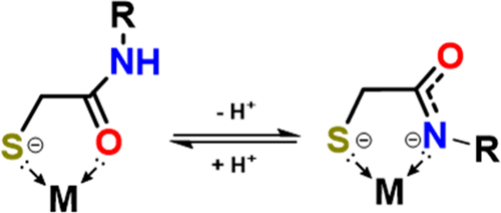
Protonation-State-Dependent Binding Modes of Thiocarboxamide Ligand

We present below multiple derivatives of the peptide analogues that present S, O, and N binding sites, whose binding properties are governed by steric/electronic effects, deprotonation, and/or chemical reduction while maintaining a dimanganese core, albeit with a significant change in bridging atoms, N or O, as shown in Scheme 2. The effects of modification of substituents at various positions on the peptide-like ligands result in dimanganese reduction products that consistently maintain the dimeric composition with such cysteine-like ligands.

## Classes of Compounds and Their Reductions

**Figure 1:**
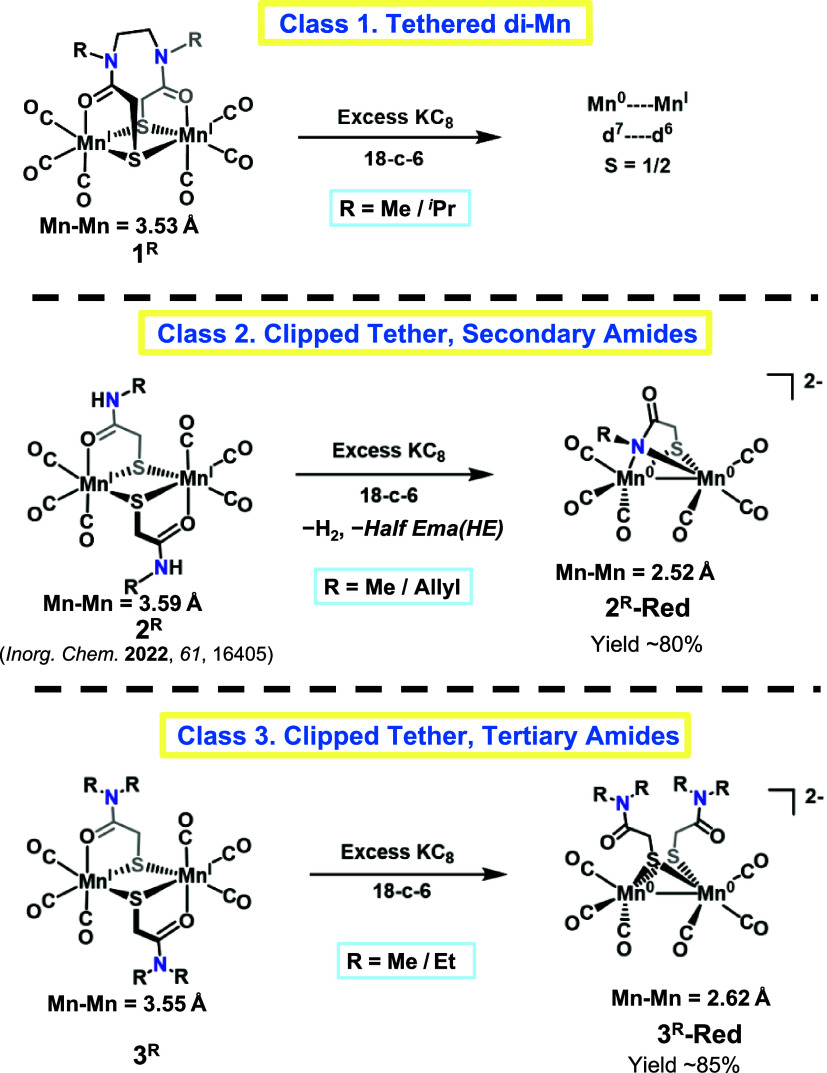
Classes of compounds and their reduced forms are found in this study. THF was used as solvent in all reactions.

There are three classes of S-bridged dimanganese complexes presented in this study, with their reduction products described in Figure 1.

### Reductions in Class **1**

Complex **1** is the introductory structure resulting from the *tethered* S–O, binucleating ligand.^26^ It presents each manganese tricarbonyl unit with two bridging thiolate sulfurs and one carboxy oxygen, which is described above and in Scheme 1. Attempts to reduce Complex 1 result in deprotonation of the amide backbone, followed by further decomposition, and do not readily yield an isolable product. The complex can be derivatized at the amide nitrogen with alkyl groups, such as methyl or isopropyl. This allowed for the reduction of the **1**^***i*****Pr**^ using excess KC_8_, resulting in a paramagnetic species with EPR signal at *g* = 1.99, Figure S5, suggesting a mixed-valent Mn^0^–Mn^I^ species having an *S* = 1/2 spin state. Repeated attempts to crystallize and obtain the molecular structure of this reduced species, compound **1-Red**, were not successful. These results suggest that the rigidity of the tethered binding sites prohibits electron uptake via the same mechanism discussed below in classes 2 and 3, which are stabilized through structural rearrangement to butterfly rhombs featuring Mn···Mn metal bonds.

### Reductions in Class **2**

Complex **2**, Scheme 1b, is derivatized at the amide-N in the forms of N-Me, complex **2**^**Me**^, or N-Allyl, **2**^**allyl**^ (Class 2, Secondary Amides), as shown in Figure 1. On exposure of a THF solution of dimeric **2**^**Me**^ and 18-crown-6 to excess potassium graphite (KC_8_), a quick color change from bright yellow to orange occurred, evolving into a deep red color. Mass spectral analysis in the negative-ESI mode found a single main species at *m*/*z* = 381.86 which corresponds to the chemical formula of the original compound **2**^**Me**^ minus one thiocarboxamide ligand, designated as *half-ema***HE**, Figure S7.

**Figure 2:**
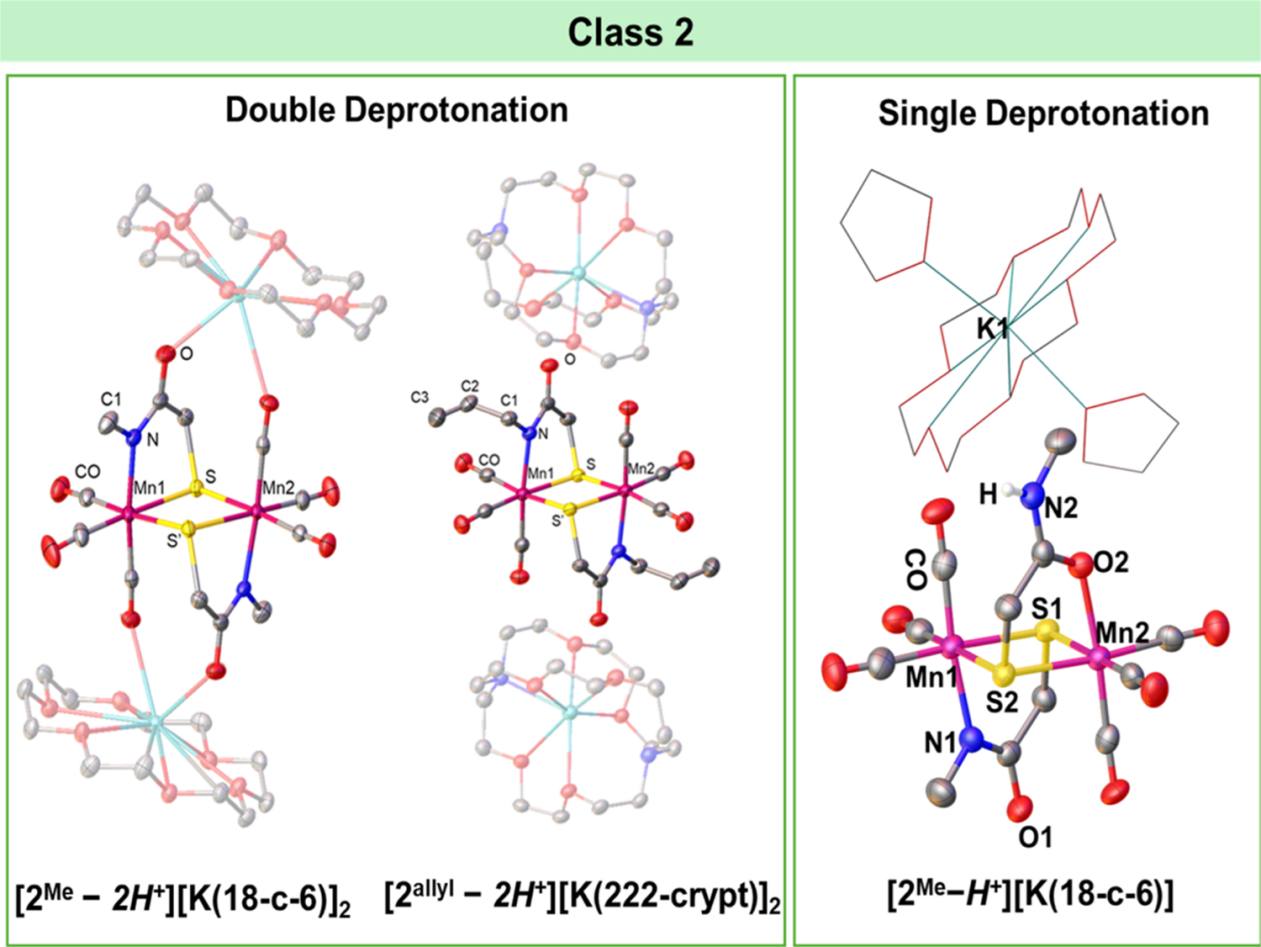
Single-crystal X-ray structures of doubly and singly deprotonated **2**^**R**^ (R = methyl, allyl) species. Note that for the single deprotonated species, both S–O and S–N coordination sites are utilized. Countercation is shown as a wireframe for clarity in the singly deprotonated species.

The formulation of the metal-containing product resulting from reduction of the *clipped tether***2**^**Me**^, yielding compound **2**^**Me**^**-Red**, was confirmed by XRD analysis, Figure 2, as a dianionic species **[Mn**_**2**_**HE]**^**2–**^ containing two Mn^0^(CO)_3_ moieties bridged by one **HE** ligand through the thiolate and a deprotonated amide N donor. The detailed crystallographic description of **2**^**Me**^**-Red** is discussed later. Headspace analysis of the reduction reaction via gas chromatography detected H_2_, Figure S8, indicating that the strong reductant has also reductively deprotonated the amide-H. This suggests that the observed reductive transformation would require 4e^***–***^ overall, two for the reduction of the Mn(I) centers, and an additional two for the amide-H deprotonation. The stepwise deprotonation of complex **2**^**Me**^ is addressed in a later part of this manuscript.

### Reduction of Class **3**

Complexes of Class **3** were developed in attempts to decouple deprotonation from reduction. The solid-state molecular structures of **3**^**Me**^ and **3**^**Et**^ were obtained in the *anti*-configuration (Figures S30 and S31). Reaction of **3**^**Et**^ with KC_8_ displays a color change from bright yellow to deep red within minutes with IR shifts almost identical to those of **2**^**R**^**-Red (R = Me, Allyl)**, indicating a similar orientation of the diatomic CO ligands within the butterfly rhomb (Figures S19 and S20). The molecular structure of this reduced product, compound **3**^**Et**^**-Red**, was obtained for the ethyl derivative with K^+^ as countercation encapsulated in 2,2,2-cryptand, confirming the proposed structure. Details of the molecular structure of **3**^**Et**^**-Red** are discussed in the Notable Crystallographic Features section, vide infra.

## Stepwise Deprotonation of the Clipped Tether, Secondary Amides

**Scheme 3:**
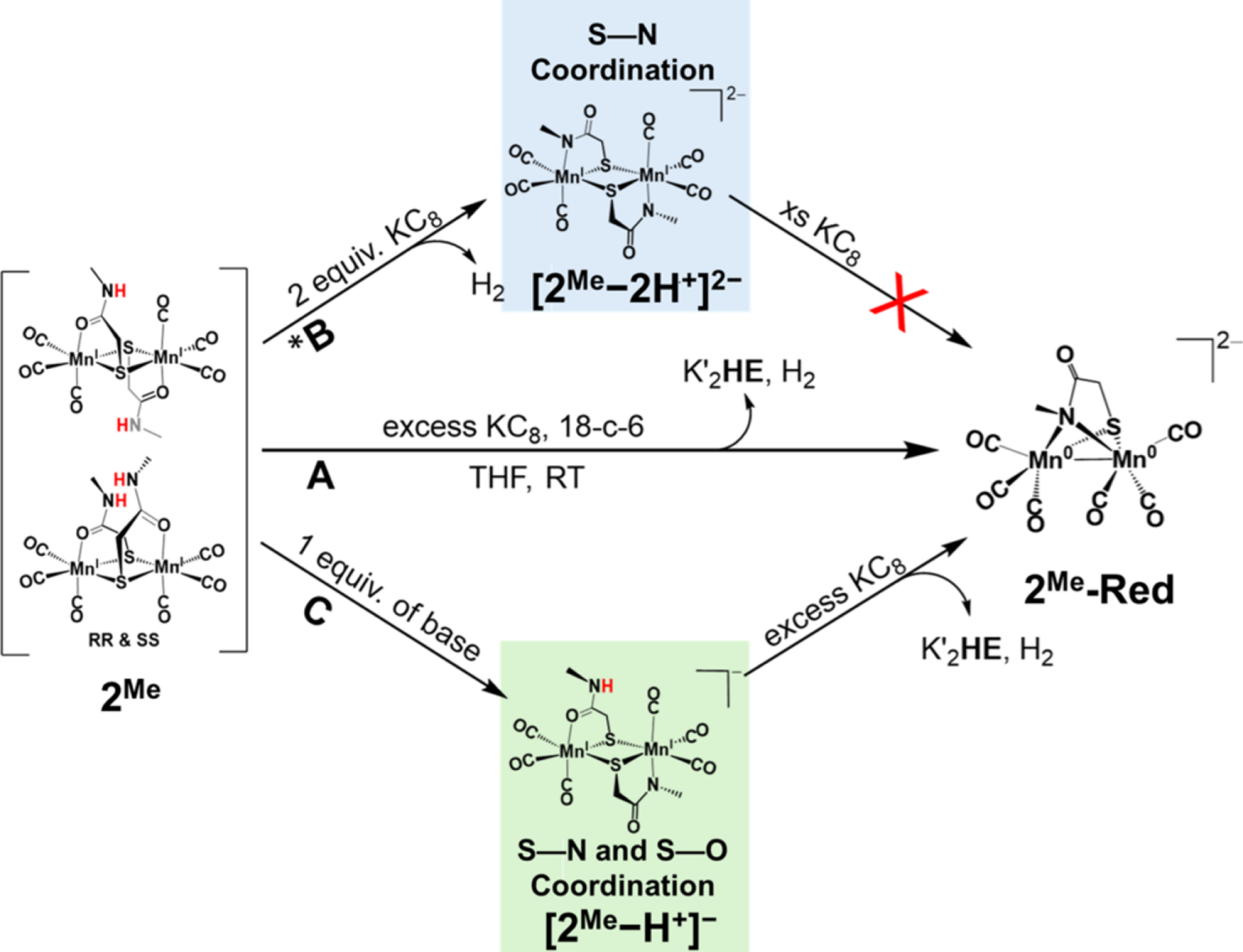
(A) Reaction of **2**^**Me**^ with an excess amount of KC_8_ that leads to the formation of **2**^**Me**^**-Red**; (B) Formation of doubly deprotonated [**2**^**Me**^-2*H*^**+**^]^**2–**^ from 2 equiv of KC_8_ (*or strong base) followed by further reaction with KC_8_; and (C) Formation of singly deprotonated [**2-***H*^**+**^**]**^**–**^ upon reaction with 1 equiv of NaH or KO^*t*^Bu followed by reaction with excess KC_8_ to form **2**^**Me**^**-Red**; K′ = [K(18-*c*-6)]

The reaction of complex **2**^**Me**^ with just 2 equiv of KC_8_, Scheme 3, path B, resulted in a new dianionic diamond core complex having Mn^I^–Mn^I^ configuration, determined to be the doubly deprotonated product of **2**, **[MnHE]**_**2**_^**2–**^ or **[2**^**Me**^**–2H**^**+**^**]**^**2–**^. Compound **[2**^**Me**^**–2H**^**+**^**]**^**2–**^ can alternatively be obtained from the reaction of **2**^**Me**^ with a strong base (Na/KO^t^Bu or NaH) as shown in Scheme 3. The products from the sodium-based reactions generally yielded low-quality crystals, with only one [Na(18-*c*-6)]^+^ structure sufficient for XRD analysis, see Figure S34. However, the spectroscopic similarity of products derived from both sodium and potassium reagents suggests that the structures of the core dimanganese units are not defined by the counterion; that is, they are the same. Deprotonation of the amide-H is indicated by the significant 48 cm^–1^ shift of the amide C=O band (Figure S6b) as well as the disappearance of the resonance corresponding to the amide-H in the ^1^H NMR spectrum, Figure S9. Most notably, the XRD analysis of this deprotonated product, obtained both with **2**^**Me**^ and **2**^**allyl**^, found the binding mode of the amide has switched from the carboxamide-O to deprotonated amide-N, Figure 2 (double deprotonation), while maintaining the Mn•Mn distance beyond bonding (3.55 Å). Interestingly, further reaction of **[2**^**Me**^**–2H**^**+**^**]**^**2–**^ with excess KC_8_ does not result in the reduced species **2**^**Me**^**-Red**. Cyclic voltammetry for complex **[2**^**Me**^***-2H***^***+***^**]**^**2–**^ shows an irreversible reduction event at −2.87 V vs Fc^+/0^, shifted cathodically over 600 mV from the reduction potential of the neutral **2**^**Me**^ complex. This reduction potential should still be accessible by KC_8_,^38^ making the observed lack of further reduction all the more unexpected (Figure S12).

The necessity of the amide-H to the formation of **2-Red** upon reacting with excess KC_8_ is highlighted in path C, Scheme 3. The single deprotonation of **2**^**Me**^, in which only one amide-H gets deprotonated, results in monoanionic **[2**^**Me**^**–*****H***^**+**^**]**^**–**^ shown in Figure 2, i.e., single deprotonation. The formation of **[2**^**Me**^**–*****H***^**+**^**]**^**–**^ can be observed through FT-IR spectroscopy upon the slow addition of ^t^BuO^–^ into a THF solution of **2**^**Me**^, Figure S6c. The slight shift of ca. 20 cm^–1^ toward lower wavenumber of all ν(CO) bands, represents a smaller displacement compared to the doubly deprotonated product **[2**^**Me**^***–2H***^***+***^**]**^**2–**^. Additionally, two amide C=O stretches are observed at 1610 and 1562 cm^–1^ corresponding to both the protonation levels and binding modes of the amide. We conclude these observations are consistent with a single deprotonation of one amide-H within the complex. The molecular structure of **[2**^**Me**^**–*****H***^**+**^**]**^**–**^ is shown in Figure 2 finding both {S,N} and {S,O} binding from the ligand. The reaction of **[2**^**Me**^**–*****H***^**+**^**]**^**–**^ with KC_8_ led to the formation of the reduced species [**2**^**Me**^**-Red]** in moderate yield as indicated by the appearance of the characteristic cluster of bands at ca. 1800 cm^–1^, Figure S6c, red trace. This observation implies that the presence of the amide protons is crucial for the formation of **2**^**Me**^**-Red**, in which the reduction of the Mn(I) centers most likely occurs concomitantly with the reductive deprotonation of the amide-H.

**Figure 3:**
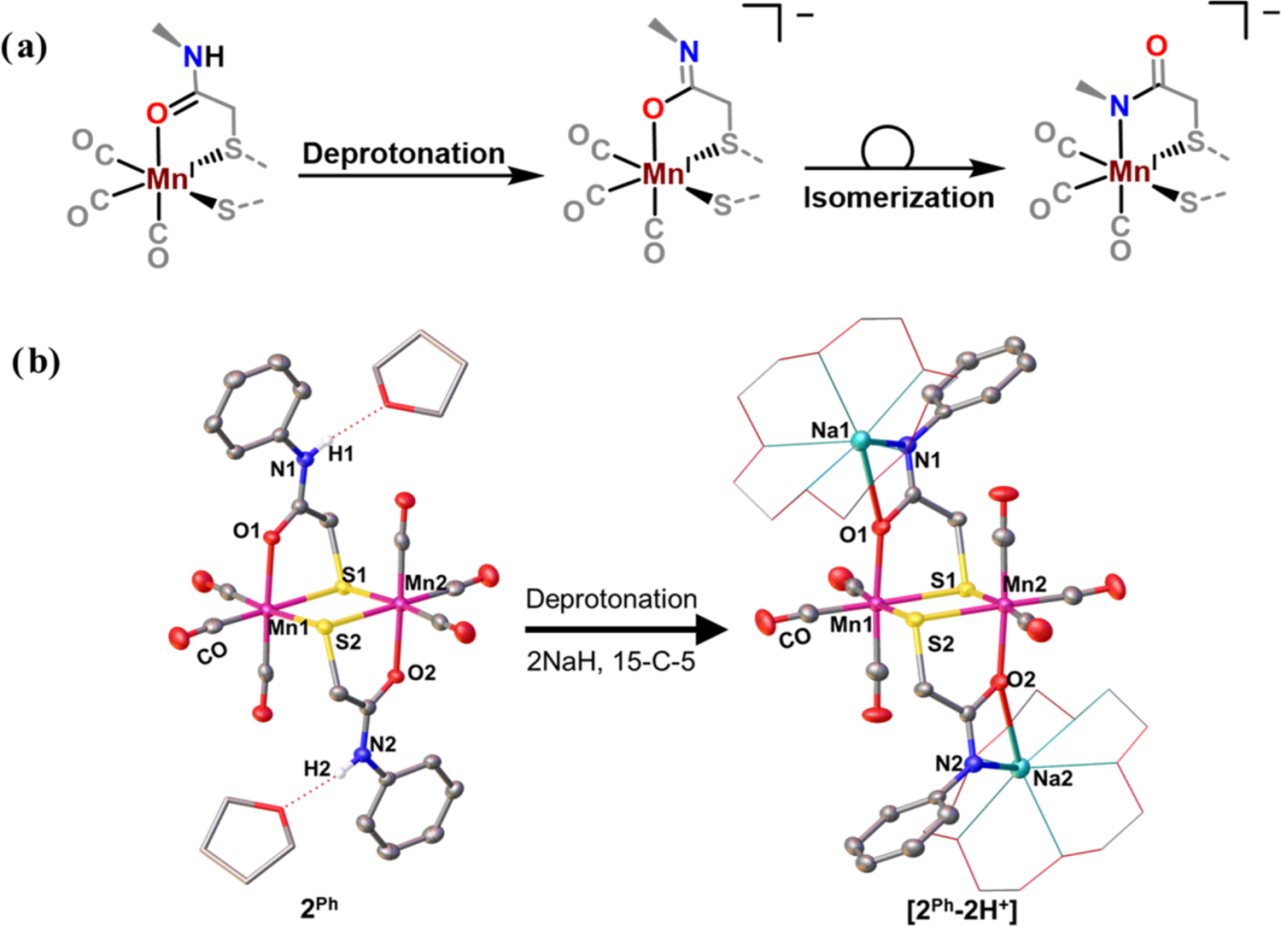
(a) Hypothesized stepwise binding mode switching of an amide. (b) Deprotonation of **2**^**Ph**^ leads to the formation of an O-bound deprotonated species [**2**^**Ph**^**–2*****H*****+**]^2–^.

The single-crystal X-ray structures shown in Figure 2 of the compound resulting upon deprotonation of **2**^**Me**^ and **2**^**allyl**^ demonstrate the amide binding mode switches from carboxamide-O to deprotonated amide-N. Such a change in amide coordination was previously observed in studies with tripodal-type ligands derivatized with amino acids.^39,40^ Notably, binding through deprotonated amide-N is suggested as the dominant interaction between transition metals that find entrapment in a polypeptide or protein backbone.^33,41−49^ Theoretically, this binding mode switching might occur sequentially, as depicted in Figure 3a, in which deprotonation initially results in an imidate group that binds to a Mn(I) center through an O atom, followed by an O- to N-bound isomerization.

Density functional theory calculations were performed, and it was found that the conversion from an {S,O} to {S,N} chelate likely involves monomeric intermediates nostalgic of our previous report.^27^ After deprotonation, the N-bound dimer is more favorable by about 15 kcal/mol, Figure S37, indicating thermodynamic control. Initial dissociation of the oxygen to open a coordination site and allow for O to N-bound isomerization has an unlikely high energy intermediate, Figure S36. However, one possible low energy transition state was found that involves initial cleavage of the dimer to 5-coordinate 16e^–^ monomeric fragments, followed by linkage isomerization through an η^3^-{N,C,O} transition state. Further details of these preliminary DFT computations are given in the Supporting Information.

Experimental evidence that supports the proposed initial formation of the O-bound imidate complex upon deprotonation is observed with an aryl derivatized complex, **2**^**Ph**^ (Figure 3b). The formation of **[2**^**Ph**^***-2H***^***+***^**]**^**2–**^ via 2 equiv of NaH leads to a shift in the diatomic ν(CO) regions very similar to **2**^**Me**^, Figure S16. Interestingly, the XRD analysis of the structure shown in Figure 3b shows an O-bound imidate strongly ion-paired with the Na^+^ counterion ensconced in the 15-c-5 macrocycle. Seemingly, the phenyl group bound directly to the amide-N results in a species that does not isomerize to the N-bound form. Whether this is due to steric or electronic control remains in question.

### Notable Crystallographic Features

#### Ion Pairing in **2**^**Red**^

**Figure 4:**
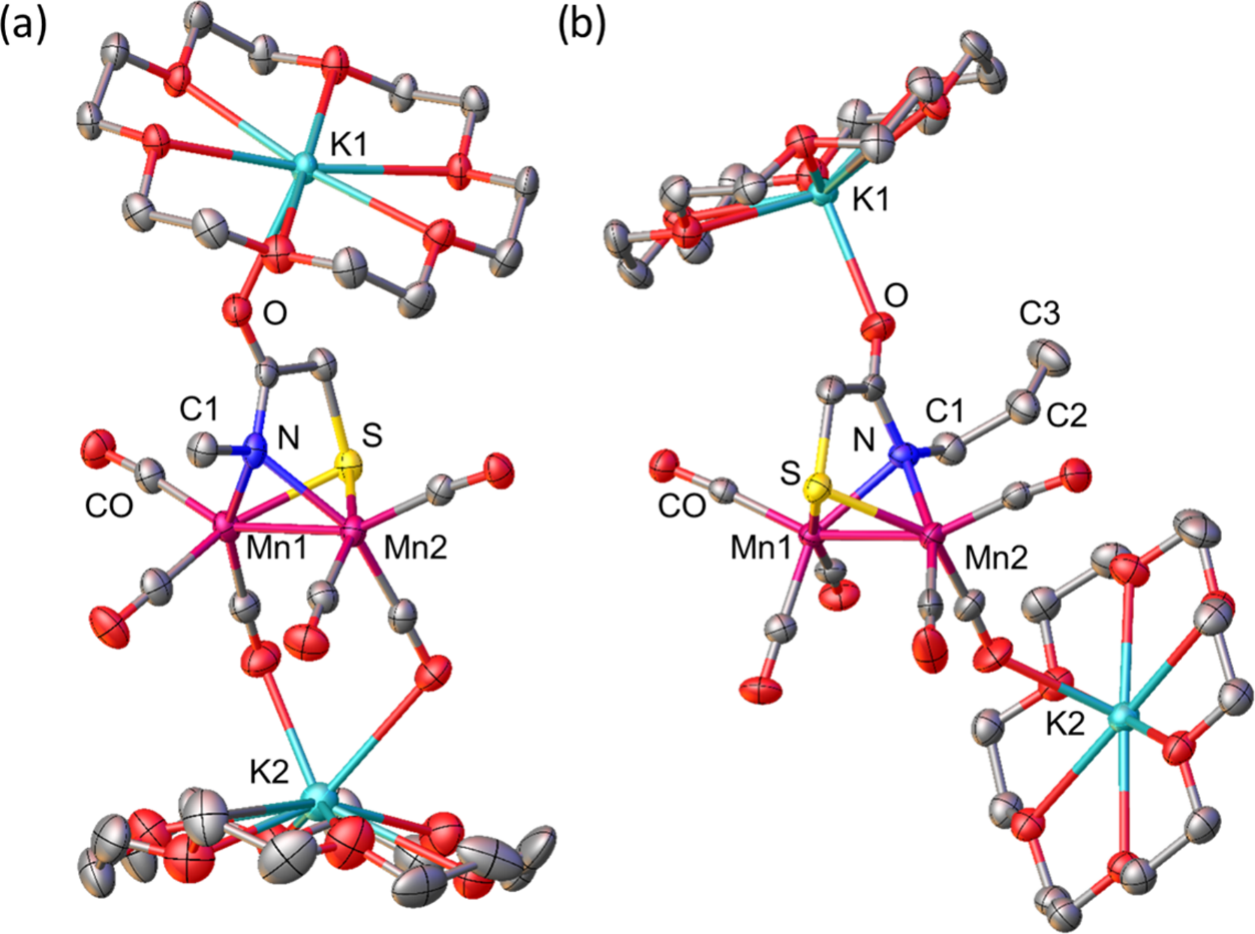
Thermal ellipsoid plots at 50% probability of (a) compound **2**^**Me**^**-Red** (Mn–Mn = 2.52 Å) and (b) compound **2**^**allyl**^**-Red** (Mn–Mn = 2.53 Å).

Single crystals suitable for XRD crystallography of the reduced products **2**^**Me**^**-Red** and **2**^**allyl**^**-Red** were obtained by Et_2_O/THF layer diffusion. As depicted in Figure 4a, the reduced product **2**^**Me**^**-Red** in the solid state has an Mn_2_ core that is sandwiched between two [K(18-*c*-6)]^+^ countercations. The core metal complex features a [Mn_2_SN] butterfly rhomb in which the two Mn(CO)_3_ moieties are bridged by a thiolate S and a deprotonated carboxamide-N from a single **HE** ligand. The molecule has overall *pseudo*-*C*_*s*_ symmetry where the local geometry around each Mn center is very close to square pyramidal with an τ_5_ value of 0.1. The metal is displaced by ca. 0.4 Å from a plane defined by the μ-{S,N} donors and two CO ligands. The charge balance between the 2^–^ charged ligand and the two K^+^ countercations indicates that the oxidation states of the metal centers are both Mn(0), d^7^. Consequently, the distance between the two 17*e*^*–*^ metal centers is found to be very short and within bonding distance (2.52 Å). So far, this is the shortest μ-S-bridged Mn–Mn single bond known, as reported values range from 2.58 to 2.92 Å.^4−7,50−59^ The bonding interaction between the two 17-electron metal centers is also evidenced by the diamagnetic, EPR-silent characteristic of the compound.

The molecular structure of an allyl derivative, **[Mn**_**2**_**HEA]**^**2–**^, **2**^**allyl**^, was also obtained from XRD analysis, Figure 4b. The overall structures of the dimanganese core in **2**^**allyl**^**-Red** are very similar to **2**^**Me**^**-Red** except that the local environments around the Mn centers are more distorted from an ideal square pyramidal geometry, τ_5_ = 0.3, and there is a slight dissymmetry between the two sites of **2**^**allyl**^**-Red**; a slight torsion of 20° when viewed along the Mn–Mn axis, Figure S33. This is presumably caused by the difference in the solid-state interaction of the two molecules with their respective [K(18-*c*-6)]^+^ countercations. In both cases, one (the top) [K(18-*c*-6)]^+^ associates with the carboxamide-O (K1–O1, 2.67 Å in **3** and 2.61 Å in **2**^**allyl**^**-Red**), while the other (the bottom one in the figure) associates with the O atom of the CO ligands. In **2**^**Me**^**-Red**, the bottom [K(18-*c*-6)]^+^ is within an interacting distance with the basal CO ligands on both Mn sites with K2–O distances of 2.83 and 2.94 Å. On the other hand, the bottom [K(18-*c*-6)]^+^ in **2**^**allyl**^**-Red** only interacts with the basal CO ligand on one Mn site (K2–O, 2.70 Å), which ultimately accounts for the dissymmetry in the molecule. This solid-state ion-pairing interaction where electron-rich metal carbonyls act as bases toward Lewis acids is well-known and has been observed with Mn^0^ carbonyl complexes.^25,57^ In solution, the IR spectra of **2**^**Me**^**-Red** and **2**^**allyl**^**-Red** are identical, Figure S3, suggesting similar solution structures, and the site-selective ion-pairing interaction in **2**^**allyl**^**-Red** comes from the solid-state packing effects.

#### Reduced Product of **3**

**Figure 5:**
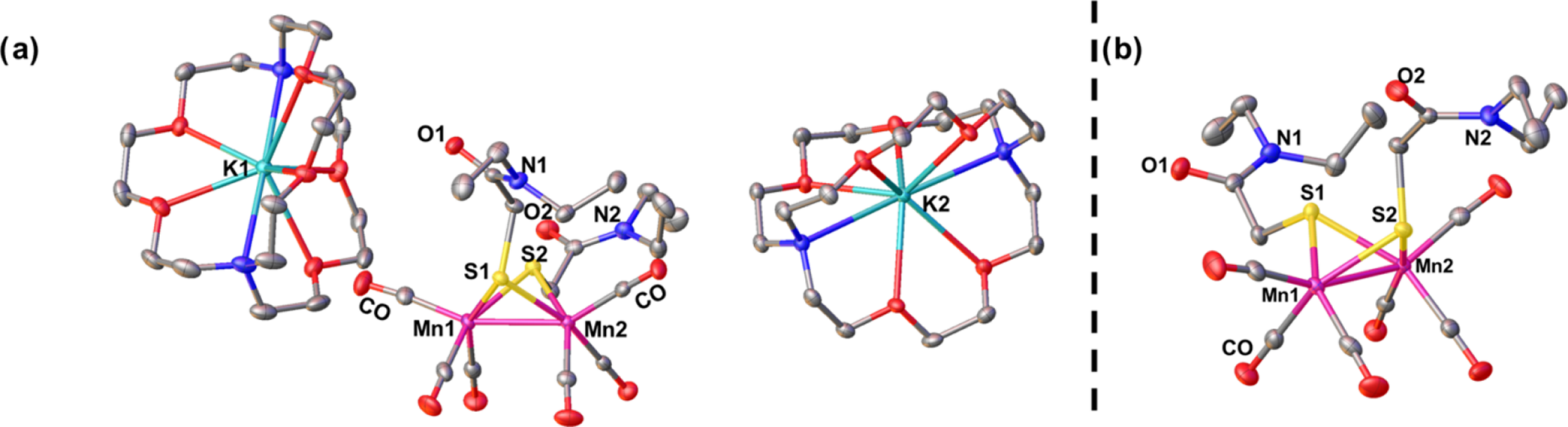
Molecular structure of **3**^**Et**^**-Red** (a) with [K(2,2,2-cryptand)]^+^ countercations displayed and (b) rotated perspective of dianionic unit with countercations removed for the sake of clarity. Thermal ellipsoids were plotted at 50% probability; Mn–Mn = 2.61 Å. H atoms are omitted for clarity.

The molecular structure of this reduced product, compound **3**^**Et**^**-Red**, was obtained for the ethyl derivative with K^+^ as countercations encapsulated in 2,2,2-cryptand, hence noninteracting with the anionic dimanganese complex. The structure is a butterfly rhomb, as shown in Figure 5b. Complex **3**^**Et**^**-Red** displays a dianionic dimanganese core similar to **2-Red** with an Mn–Mn distance of 2.61 Å. However, unlike compound **2-Red**, all atoms are conserved in compound **3**^**Et**^**-Red**, and the butterfly rhomb exhibits two μ-S bridges instead of two μ-{S,N}. The dissociation of the amide-O from the Mn centers led to a structural change from [2Mn2S] diamond core to butterfly rhomb. The overall 2^–^ charge and the short Mn–Mn distance found in **3**^**Et**^**-Red** suggest the same Mn^0^–Mn^0^ configuration as in **2**^**Me**^**-Red**.

## Concluding Comments

**Figure 6:**
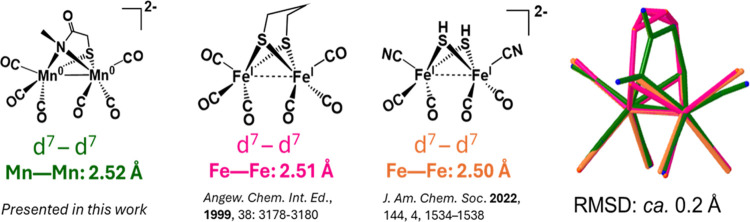
Structures of [μ-(S–S)Fe_2_(CO)_*x*_] in comparison with **2-Red** and overlays.

Interestingly, the core structures of compounds **2-Red** and **3-Red** resemble the [Fe_2_^I^S_2_(CO)_4_(CN)_2_] dimeric intermediate proposed to be along the assembly line toward the [FeFe]-H_2_ase H-cluster. This is expected as the d^7^–d^7^ configuration coming from the Mn(0) centers in compounds **2-Red** and **3-Red** is isoelectronic with the Fe(I)–Fe(I) configuration. The bridging atoms are competent to hold two metals together in the butterfly arrangement that permits the metals to accommodate the electron surfeit by forming metal–metal bonds. Additionally, they are isostructural as evident from overlaying the structures of compound **2-Red**, the proposed [Fe_2_^I^S_2_(CO)_4_(CN)_2_] intermediate, and the [Fe_2_(CO)_6_(μ_2_-S-pdt)] model compound, Figure 6.^60,61^ In this regard, compounds **2-Red** and **3-Red** are examples of dimanganese complexes that exhibit the edge-shared, bisquare pyramidal structure characteristic of the [FeFe]-H_2_ase active site model compounds. Most of the known dimanganese butterfly structures contain either a bridging CO or H^–^ ligand, creating local octahedral geometry, or have an asymmetric octahedral–square pyramidal geometry.^4−7^ (Note: a preliminary abstract deposited by Berggren et al. mentions a potentially closer dimanganese mimic of the diiron active site^62^).

As key takeaways from the results of this study, we offer the following observations:

- Biomimetics of sulfur-containing peptides show the ability to participate in the binding of low-valent manganese carbonyl [Mn(CO)_3_] fragments, using S, N, and/or O donor sites, whose interconversions can accommodate differences in charge from protonation and reduction levels in dimeric Mn(CO)_3_ complexes.
- Thiocarboxamide bidentate ligands, as cysteine mimics, have permitted observation of two rhombic geometries for S-bridged dimanganese units, diamond core, and butterfly rhomb, the latter moving the resultant Mn^0^ Mn^0^ centers to within a short, characteristic bonding distance, resulting in diamagnetism.
- The versatility of peptide-like units or fragments facilitates uptake of low-valent metals in the form of carbonyls, particularly manganese. An attractive hypothesis is that this chemistry connects arguments that primordial peptide fragments could have been a part of cooperative catalysis in their own buildup programs and those of metals. The uptake of low-valent metal carbonyls is worth further explorations in biosynthetic paths and in metal transport. It also points to a new application of bioorganometallic chemistry toward understanding metal-binding peptides as the paradigm of enhanced absorption and bioavailability of minerals.
- The intricacies of the binding paradigms for thiocarboxamides appear to be especially well matched for low-valent manganese ligand preferences. Our attempts to extend to analogous iron carbonyls in either dimeric or monomeric forms have yet to be successful.

## Experimental Section

All air-free reactions and manipulations were performed using standard Schlenk-line and syringe/rubber septa techniques under N_2_ or in Ar/N_2_ atmosphere gloveboxes. Dry solvents were purified and degassed via a Bruker solvent system. THF solvent was distilled over Na/benzophenone. Reagents were purchased from commercial sources and used as received. Compound 2^Me^ and its derivatives, 2^allyl^ and 2^Ph^, and compound 3^Me/Et^ along with the tethered complexes 1^Me/i-Pr^ were synthesized using the previously reported procedure.^26,27^

**Caution!***Potassium graphite is highly flammable when in contact with air and water and should be handled with care. Potassium tert-butoxide and sodium hydride are strong bases that react violently with water. All manipulations were performed in an Ar- or N*_2_-*filled glovebox and properly quenched prior to disposal.*

### Physical Measurements

Electrospray ionization mass spectrometry (ESI-MS) was performed in the Laboratory for Biological Mass Spectrometry at Texas A&M University. Infrared spectra were recorded on a Bruker Tensor 37 spectrometer using a CaF_2_ liquid cell with a path length of 0.2 mm path length. ^1^H and ^13^C NMR spectra were recorded by using a Bruker Avance III 400 MHz Broadband spectrophotometer operating at 400.1 MHz and an Inova 500 MHz spectrophotometer operating at 125.6 MHz, respectively. Data for X-ray structure determination was collected at 110 K using Bruker D8-Venture with CPAD-Photon III detector or Bruker D8-Quest with CPAD-Photon II detector, with graphite monochromated Cu radiation source (λ = 1.5406 Å) or Mo radiation source (λ = 0.71073 Å). All crystals were coated with paraffin oil and mounted on a nylon or Mitegen loop. The structures were solved by intrinsic phasing method (SHELXT).^63^ All were refined by standard Fourier techniques against F square with a full-matrix least-squares algorithm using SHELXL.^64^ All nonhydrogen atoms were refined anisotropically, while hydrogen atoms were placed in calculated positions and refined isotropically. Graphical representations were generated using Olex2 software package.^65^ Electron paramagnetic resonance (EPR) spectra were collected on an X-band Bruker Elexsys EPR spectrometer cooled to 4 K.

### Gas Chromatography

Gas chromatography was done in an Agilent Trace 1300 GC equipped with a custom-made 120 cm stainless steel column packed with Carbosieve-II from Sigma-Aldrich and a thermal conductivity detector. The carrier gas was argon. The detector temperature was at 250 °C, while the column was kept at 200 °C. Approximately 300 μL of gas was injected via 0.5 mL of Valco Precision Sampling Syringe.

### Computational Method

DFT calculations were performed using the TPSSTPSS6 functional, and the triple-ζ basis set 6-311+G^66−69^ for Mn and 6-311++G(d,p) for nonmetals in Gaussian16 Revision C.01.11 Transition state structures were located using the Synchronous Transit-Guided Quasi-Newton method from the structures provided from reactants and products with the initial guess for the transition states (qst3).^70^ Free energies of calculated minima and transition states were corrected for solvation via the SMD model.^71^

### General Ligand Synthesis

**Step 1:** To an ice-cold 1:1 H_2_O/THF mixture containing 1 equiv of corresponding primary amine (or diamine) and 4 equiv of K_2_CO_3_, 1.1 equiv of chloroacetyl chloride (or 2.2 equiv for the ema derivatives) was added dropwise. The reaction mixture was then stirred at RT for a minimum of 3 h. The product was then extracted with DCM, washed with brine, and left to stand over MgSO_4_. The resulting solution was concentrated, and the resulting product was recrystallized from hexane or purified via silica column chromatography.

**2-chloro-*N*-allylacetamide**: ^1^H NMR (400 MHz, CDCl_3_, 298 K): δ 6.65 (br, 1H), 5.25–5.18 (m, 2H), 4.08 (s, 2H) and 3.94 (tt, 2H, ^3^*J*_HH_ 5.7 Hz, ^4^*J*_HH_ 1.45 Hz). **2-chloro-*N*,*N*-dimethylacetamide**: ^1^H NMR (400 MHz, CDCl_3_, 298 K): δ 4.07 (s, 2H), 3.08 (s, 3H) and 2.97 (s, 3H). **2-chloro-*N*,*N*-diethylacetamide**: ^1^H NMR (400 MHz, CDCl_3_, 298 K): δ 4.05 (s, 2H), 3.39 (q, 2H, ^3^*J*_HH_ 7.2 Hz), 3.37 (q, 2H, ^3^*J*_HH_ 7.1 Hz), 1.23 (t, 2H, ^3^*J*_HH_ 7.2 Hz) and 1.14 (t, 2H, ^3^*J*_HH_ 7.1 Hz). **2-chloro-***N***-phenylacetamide**: ^1^H NMR (400 MHz, CDCl_3_, 298 K): δ 8.23 (br, 1H), 7.55 (dt, 2H, ^3^*J*_HH_ 7.5 Hz, ^4^*J*_HH_ 1.2 Hz), 7.36 (tt, 2H, ^3^*J*_HH_ 7.5 Hz, ^4^*J*_HH_ 1.9 Hz), 7.18 (tt, 2H, ^3^*J*_HH_ 7.5 Hz, ^4^*J*_HH_ 1.2 Hz) and 4.19 (s, 2H). ***N*,*N*′-ethylene-bis(2-chloro-***N***-methylacetamide)**: ^1^H NMR (400 MHz, CDCl_3_, 298 K): δ 4.04 (s, 4H), 3.60 (s, 4H) and 3.11 (s, 6H). ***N*,*N*′-ethylene-bis(2-chloro-***N***-isopropylacetamide)**: ^1^H NMR (400 MHz, CDCl_3_, 298 K): δ 4.10 (s, 4H), 3.98 (qu, 1H, ^3^*J*_HH_ 6.3 Hz), 3.34 (s, 4H) and 1.29 (d, 12H, ^3^*J*_HH_ 6.3 Hz).

**Step 2:** 1.5 equiv of thioacetic acid was added dropwise to an ice-cold MeOH solution containing 1.3 equiv of KOH. The resulting yellow solution was then transferred to a Schlenk flask containing 1 equiv of corresponding chloroacetamide in EtOH, and the reaction mixture was refluxed under N_2_ for 1 h. It was then cooled to room temperature and stirred for another 12 h. Then, the reaction mixture was dissolved in DCM and washed with H_2_O. The organic phase was then dried with brine then MgSO_4_, concentrated, and the resulting crude product was recrystallized from hexane or purified via silica column chromatography.

**2-thioacetoxy-*N*-allylacetamide**: ^1^H NMR (400, CDCl_3_, 298 K): δ 6.35 (br, 1H), 5.83–5.73 (m, 1H), 5.15–5.09 (m, 2H), 3.82 (tt, 2H, ^3^*J*_HH_ 5.7 Hz, ^4^*J*_HH_ 1.45 Hz), 3.53 (s, 2H) and 2.38 (s, 3H).

**2-thioacetoxy-*N*,*N*-dimethylacetamide**: ^1^H NMR (400 MHz, CDCl_3_, 298 K): δ 3.82 (s, 2H), 3.08 (s, 3H), 2.96 (s, 3H) and 2.36 (s, 3H). **2-thioacetoxy-*N*,*N*-diethylacetamide**: ^1^H NMR (400 MHz, CDCl_3_, 298 K): δ 3.83 (s, 2H), 3.38 (qd, 2H, ^3^*J*_HH_ 7.1 Hz, ^4^*J*_HH_ 2.1 Hz), 2.37 (s, 3H), 1.23 (t, 2H, ^3^*J*_HH_ 7.2 Hz) and 1.12 (t, 2H, ^3^*J*_HH_ 7.2 Hz). **2-thioacetoxy-***N***-phenylacetamide**: ^1^H NMR (400 MHz, CDCl_3_, 298 K): δ 8.09 (br, 1H), 7.49 (dt, 2H, ^3^*J*_HH_ 7.7 Hz, ^4^*J*_HH_ 1.1 Hz), 7.32 (tt, 2H, ^3^*J*_HH_ 7.7 Hz, ^4^*J*_HH_ 1.9 Hz), 7.11 (tt, 2H, ^3^*J*_HH_ 7.5 Hz, ^4^*J*_HH_ 1.1 Hz), 3.66 (s, 2H) and 2.45 (s, 3H). ***N*,*N*′-ethylene-bis(2-thioacetoxy-***N***-methylacetamide)**: ^1^H NMR (400 MHz, CDCl_3_, 298 K): δ 3.77 (s, 4H), 3.56 (s, 4H), 3.09 (s, 6H) and 2.34 (s, 6H). ***N*,*N*′-ethylene-bis(2-thioacetoxy-***N***-isopropylacetamide)**: ^1^H NMR (400 MHz, CDCl_3_, 298 K): δ 4.00 (qu, 2H, ^3^*J*_HH_ 6.7 Hz), 3.83 (s, 4H), 3.25 (s, 4H), 2.31 (s, 6H) and 1.22 (d, 12H, ^3^*J*_HH_ 6.7 Hz).

### General Synthesis for Class 1^R^ (R = Me/^i^Pr)

A mixture of 0.09 mmol of the S-acetylated ethylene-bis(N,N′-dialkyl-N,N′-mercaptoacetamide) ligand, 8 mg (0.2 mmol) of NaOH, and 20 mg (0.1 mmol) of Zn(OAc)_2_.2H_2_O was dissolved and stirred in 15 mL of MeOH for 15 min. A 10 mL portion of MeOH containing 50 mg (0.18 mmol) of Mn(CO)_5_Br was then added, and the resulting mixture was kept in the dark and heated at 60 °C. The reaction reached completion after 3 h, upon which the solvent was evaporated under reduced pressure and the resulting yellow residue was dissolved in THF. The yellow THF solution was then filtered through Celite, and the solvent was evaporated in vacuo to yield a yellow powder. XRD quality crystals were obtained by THF/pentane vapor diffusion. Spectroscopic yield: 80–90%.

1^Me^: IR in THF (ν_CO_, cm^–1^): 2025 (w), 2006 (s), 1913 (s, br), and 1568 (m, amide C=O).

1^iPr^: IR in THF (ν_CO_, cm^–1^): 2025 (w), 2006 (s), 1913 (s, br) and 1568 (m, amide C=O). ^1^H NMR (400 MHz, DMSO-*d*_6_, 298 K): δ 4.25 (sept, 2H, ^3^J_HH_ 6.6 Hz), 4.18 (dd, 2H, ^2^J_HH_ 14.4 Hz, ^3^J_HH_ 4.4 Hz), 3.45 (d, 2H, ^2^J_HH_ 14.5 Hz), 3.37 (dd, 2H, ^2^J_HH_ 14.4 Hz, ^3^J_HH_ 4.4 Hz), 3.12 (d, 2H, ^2^J_HH_ 14.5 Hz), 1.39 (d, 6H, ^3^J_HH_ 6.6 Hz) and 1.17 (d, 6H, ^3^J_HH_ 6.6 Hz).

### General Synthesis Classes **2** and **3**

A mixture of 0.18 mmol of the corresponding thioester acetamide and 7 mg of NaOH (0.18 mmol) in 15 mL of MeOH stirred for 15 min under N_2_. 50 mg (0.18 mmol) of Mn(CO)_5_Br dissolved in 10 mL of MeOH was then added. The reaction mixture was stirred in the dark at room temperature. After the reaction reached completion, as evident from IR spectroscopy, the solvent was removed in vacuo. The resulting orange-yellow residue was then dissolved in THF, filtered through Celite, and purified through a short alumina column. The solution volume was then reduced, and precipitation via the addition of hexane afforded the crude yellow powder product. X-ray quality crystals were obtained from pentane/THF vapor diffusion.

**2**^**allyl**^: IR in THF (ν_CO_, cm^–1^): 2025 (w), 2003 (s), 1917 (s), 1902 (s), and 1608 (m, amide C=O);

**2**^**Ph**^: IR in THF (ν_CO_, cm^–1^): 2027 (w), 2007 (s), 1921 (s), 1907 (s), 1612 (m, aromatic C=C), 1598 (m, amide C=O) and 1562 (m, aromatic C=C). ^1^H – NMR (400 MHz, DMSO-*d*_6_, 298 K): δ 11.70 (s, 1H), 11.00 (s, 1H), 7.66 (s, 1H), 7.65 (s, 1H), 7.49 (t, 2H), 7.28–7.22 (m, 3H), 7.17–7.10 (m, 3H), 3.73 (dd, 2H) and 3.36 (dd, 2H).

**3**^**Me**^: IR in THF (ν_CO_, cm^–1^): 2025 (w), 2004 (s), 1917 (s), 1899 (s), and 1597 (m, amide C=O).

**3**^**Et**^: IR in THF (ν_CO_, cm^–1^): 2025 (w), 2004 (s), 1915 (s), 1900 (s), and 1597 (m, amide C=O).

**Caution!***Alkali metals and their intercalation compounds are highly flammable when in contact with air and water and should be handled with care. All reductions were performed in a nitrogen-filled glovebox and properly quenched prior to disposal.*

### Formation of [Mn_2_HE]^2–^, Compound **2**^**Me**^-Red

**Path A:** To a THF solution containing 22 mg (0.045 mmol) of 2^Me^, [MnHE]_2_ and 26 mg of 18-c-6 (0.1 mmol), 27 mg (0.2 mmol) of KC_8_ was added. Over the course of the reaction, the solution quickly changed from bright yellow to orange and finally to deep red. Depending on how much excess KC_8_ is being added, the reaction time ranges from 30 min to several hours. When the reaction reached completion, as monitored by FT-IR, the deep red solution was filtered through Celite. Crystallization via ether/THF layer diffusion afforded red crystals that were suitable for X-ray diffraction. The yield of the product is dependent on the amount of excess KC_8_ with spectroscopic yield as high as 80%. (ν_CO_, cm^–1^): 1944 (m), 1886 (s), 1832 (m), 1813 (m), and 1796 (sh); ESI-HRMS(−) *m*/*z*: 386.81 [M + H]^−^.

**Path B:** A THF solution containing 4 mg (0.035 mmol) of KO^t^Bu was added dropwise to 4 mL of THF solution containing 15 mg (0.03 mmol) of 2^Me^. Upon reaching the singly deprotonated species [2^Me^–H^+^]^−^ evident from FT-IR, 16 mg (0.06 mmol) of 18-c-6 was added followed by 16 mg (0.12 mmol) of KC_8_. A similar workup to path A was then employed. Spectroscopic yield: 35%.

#### Synthesis of [Mn_2_HE]_2_^2–^, [2^Me^–2H^+^]^2–^

To a THF solution containing 20 mg (0.04 mmol) of 2^Me^ and 25 mg (0.09 mmol) of 18-c-6, 11 mg (0.08 mmol) of KC_8_ was added and stirred at room temperature. The reaction reached completion after 15 min as monitored by FT-IR. The solution was then filtered through Celite, and the resulting orange-yellow solution was layered with diethyl ether for crystallization. IR in THF (ν_CO_, cm^–1^): 1977 (s), 1880 (s), and 1568 (m, amide C=O).

*Note: [2^Me^–2H^+^]^2–^ can be independently synthesized via deprotonation of 2^Me^ with a strong base either in THF for FT-IR monitor or DMSO-*d*_6_ for NMR. ^1^H NMR (400 MHz, DMSO, 298 K): 3.31 (s, 3H), 2.89 (d, ^2^*J*_HH_ 15.6 Hz, 1H) and 2.68 (d, ^2^*J*_HH_ 15.6 Hz, 1H).

#### Synthesis of [2^Me^–H^+^]^−^

To a THF solution containing 15 mg (0.03 mmol) of **2^Me^** and 10 mg (0.04 mmol) of 18-*c*-6, 4 mg (0.035 mmol) of KO*^t^*Bu was slowly added and stirred at room temperature. The reaction reached completion after 5 min as monitored by FT-IR. The resulting yellow solution was filtered to Celite and layered with diethyl ether for crystallization. FT-IR in THF (ν_CO_, cm^–1^): 2010 (w) 1990 (s), 1890 (s, br), 1614 (m, amide C=O) and 1574 (m, amide C=O).

#### Synthesis of [2^allyl^–2H^+^]^2–^

To a THF solution containing 31 mg (0.06 mmol) of 2^allyl^, 10 mg (0.25 mmol) of 60% w/w NaH dispersed in mineral oil was added and allowed to stir at room temperature. After 5 min, the mixture was filtered through Celite and 45 mg (0.12 mmol) of 2,2,2-cryptand was added. The resulting solution was layered with diethyl ether for crystallization. IR in THF (ν_CO_, cm^–1^): 1977 (s), 1880 (s), and 1568 (m, amide C=O). ^1^H – NMR (400 MHz, DMSO-*d*_6_, 298 K): δ 5.99 (m, 1H), 5.05 (d, 1H), 4.91 (d, 1H), 4.57 (dd, 1H), 3.73 (dd, 1H), 2.87 (d, 1H) and 2.71 (d, 1H).

#### Synthesis of [2^Ph^–2H^+^]^2–^

To a THF solution containing 37 mg (0.06 mmol) of 2^Ph^, 10 mg (0.25 mmol) of 60% w/w NaH dispersed in mineral oil was added, and the mixture was stirred at room temperature. After 5 min, the mixture was filtered through Celite and 24 μL (0.12 mmol) of 15-c-5 was added. The resulting solution was layered with diethyl ether for crystallization. IR in THF (ν_CO_, cm^–1^): 1985 (s), 1890 (s), and 1564 (m, br, amide C=O). ^1^H NMR (400 MHz, DMSO-*d*_6_, 298 K): δ 7.26 (dt, 2H, ^3^J_HH_ 7.1 Hz, ^4^J_HH_ 1.2 Hz, ^5^J_HH_ 1.9 Hz), 7.16 (tt, 2H, ^3^J_HH_ 7.1 Hz, ^5^J_HH_ 1.9 Hz), 6.87 (tt, 1H, ^3^J_HH_ 7.1 Hz, ^4^J_HH_ 1.2 Hz), 2.99 (d, 1H, ^2^J_HH_ 16.0 Hz), 2.91 (d, 1H, ^2^J_HH_ 16.0 Hz).

#### Synthesis of [3^Et^]^2–^, 3^Et^-Red

To a THF solution containing 0.05 mmol of **4** and 38 mg (0.1 mmol) of 2,2,2-cryptand, 27 mg (0.2 mmol) of KC_8_ was added. The solution immediately changed from bright yellow to dark red. After 10 min, the solution was filtered through Celite to afford a bright-red-colored solution. More product was collected by passing dry MeCN through a Celite column. XRD quality crystals were obtained from THF/diethyl ether or MeCN/diethyl ether layer diffusion. IR in THF (ν_CO_, cm^–1^): 1938 (m), 1881 (s), 1827 (m), 1809 (m), 1794 (sh), and 1624 (w, amide C=O).

## Supporting Information Available

Experimental procedures, XRD structural characterizations, spectral data, and additional supporting analysis. (PDF)
