# Supplementary material for: From [2Mn2S] Diamond Cores to Butterfly Rhombs: Transformations That Highlight Alternating Peptide Binding Sites
Source: Inorg Chem. 2024 Oct 25;63(44):21375–85. doi: 10.1021/acs.inorgchem.4c04051 (PMC11539053; doi:10.1021/acs.inorgchem.4c04051)
Supplement: Supplementary file 1 — ic4c04051_si_001.pdf [file ic4c04051_si_001.pdf]

Supporting Information

From [2Mn2S] Diamond Cores to Butterfly Rhombs: Transformations that Highlight Alternating Peptide Binding Sites

Trung H. Le, Kyle T. Burns, Manish Jana, Heather A. Arnold, Connor R. Vann, and Marcetta Y. Darensbourg*

Department of Chemistry, Texas A&M University, College Station TX 77845

Email: marcetta@mail.chem.tamu.edu

I. Experimental

General. All air-free reactions and manipulations were performed using standard Schlenk-line and syringe/rubber septa techniques under N_2 or in Ar/N_2 atmosphere gloveboxes. Dry solvents were purified and degassed via a Bruker solvent system. THF solvent was distilled over Na/benzophenone. Reagents were purchased from commercial sources and used as received. Compound $\mathbf{2}^{\mathbf{Me}}$ and its derivatives, $\mathbf{2}^{\mathbf{allyl}}$ and $\mathbf{2}^{\mathbf{Ph}}$, and compound $\mathbf{3}^{\mathbf{Me/Et}}$ along with the *tethered* complexes $\mathbf{1}^{\mathbf{Me}/\boldsymbol{i}\text{-}\mathbf{Pr}}$ were synthesized using the previously reported procedure.[1-2]

Physical measurements. Electrospray ionization mass spectrometry (ESI-MS) was performed in the Laboratory for Biological Mass Spectrometry at Texas A&M University. Infrared spectra were recorded on a Bruker Tensor 37 spectrometer using a CaF_2 liquid cell of 0.2 mm path length. ^1H and ^{13}C-NMR spectra were recorded using a Bruker Avance III 400 MHz Broadband spectrophotometer operating at 400.1 MHz and an Inova 500 MHz spectrophotometer operating at 125.6 MHz, respectively. Data for X-ray structure determination was collected at 110K using Bruker D8-Venture with CPAD – Photon III detector or Bruker D8-Quest with CPAD – Photon II detector, with graphite monochromated Cu radiation source (λ = 1.5406 Å) or Mo radiation source (λ = 0.71073 Å). All crystals were coated with paraffin oil and mounted on a nylon or Mitegen loops. The structures were solved by intrinsic phasing method (SHELXT).[3] All were refined by standard Fourier techniques against F square with a full-matrix least squares algorithm using SHELXL.[4] All non-hydrogen atoms were refined anisotropically while hydrogen atoms were placed in calculated position and refined isotropically. Graphical representations were generated using Olex2 software package.[5] Electron paramagnetic resonance (EPR) spectra were collected on an X-band Bruker Elexsys EPR spectrometer cooled to 4 K.

Gas chromatography. Gas chromatography was done in an Agilent Trace 1300 GC equipped with a custom made 120 cm stainless steel column packed with Carbosieve-II from Sigma-Aldrich and a thermal conductivity detector. The carrier gas was Argon. The detector temperature was at 250 °C while the column was kept at 200 °C. Approximately 300 μL gas was injected via 0.5 mL Valco Precision Sampling Syringe.

Synthesis.

General synthesis for derivatives of '*ema*' and 'half-*ema*' ligands from their corresponding amines:

Step 1 – formation of chloroacetamide: To an ice-cold 1:1 H_2O/THF mixture containing 1 equiv. of corresponding primary amine (or diamine) and 4 equiv. of K_2CO_3, 1.1 equiv. of chloroacetyl chloride (or 2.2 equiv. for the *ema* derivatives) was added dropwise. The reaction mixture was then stirred at RT for a minimum of 3 hours. The product was then extracted into DCM followed by washing with brine and let sit over $MgSO_4$. The supernatant was concentrated, and the resulting product was recrystallized from hexane or purified via silica column chromatography:

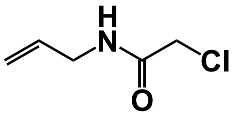

Dense colorless oil purified via silica column chromatography (1:1 Hex/EtOAc) followed by solvent removal under vacuo, yield 45%. ^{1}H-NMR (400 MHz, $CDCl_3$, 298 K): δ 6.65 (br, 1H), 5.25 – 5.18 (m, 2H), 4.08 (s, 2H) and 3.94 (tt, 2H, $^3J_{HH}$ 5.7 Hz, $^4J_{HH}$ 1.45 Hz).

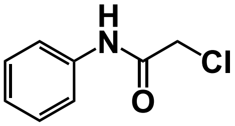

The white crystalline solid was obtained from recrystallization from hexane, yield 57.5%. ^{1}H-NMR (400 MHz, $CDCl_3$, 298 K): δ 8.23 (br, 1H), 7.55 (dt, 2H, $^3J_{HH}$ 7.5 Hz, $^4J_{HH}$ 1.2 Hz), 7.36 (tt, 2H, $^3J_{HH}$ 7.5 Hz, $^4J_{HH}$ 1.9 Hz), 7.18 (tt, 2H, $^3J_{HH}$ 7.5 Hz, $^4J_{HH}$ 1.2 Hz) and 4.19 (s, 2H).

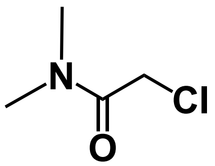

Obtained as a dense colorless oil purified via silica column chromatography, yield: 50%. ^{1}H-NMR (400 MHz, $CDCl_3$, 298 K): δ 4.07 (s, 2H), 3.08 (s, 3H) and 2.97 (s, 3H).

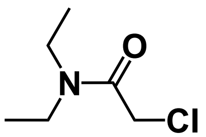

Dense colorless oil purified via silica column chromatography, yield: 45%. ^{1}H-NMR (400 MHz, $CDCl_3$, 298 K): δ 4.05 (s, 2H), 3.39 (q, 2H, $^3J_{HH}$ 7.2 Hz), 3.37 (q, 2H, $^3J_{HH}$ 7.1 Hz), 1.23 (t, 2H, $^3J_{HH}$ 7.2 Hz) and 1.14 (t, 2H, $^3J_{HH}$ 7.1 Hz).

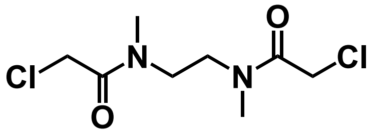

White crystalline solid obtained from recrystallization from hexane, yield 60 %. ^{1}H-NMR (400 MHz, $CDCl_3$, 298 K): δ 4.04 (s, 4H), 3.60 (s, 4H) and 3.11 (s, 6H).

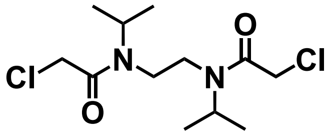

White crystalline solid obtained from recrystallization from hexane, yield 60 %. ^{1}H-NMR (400 MHz, $CDCl_3$, 298 K): δ 4.10 (s, 4H), 3.98 (qu, 1H, $^3J_{HH}$ 6.3 Hz), 3.34 (s, 4H) and 1.29 (d, 12H, $^3J_{HH}$ 6.3 Hz).

Step 2 – formation of thioester acetamides: 1.5 equiv. of thioacetic acid was added dropwise to an ice cold MeOH solution containing 1.3 equiv. of KOH. The resulting yellow solution was then transferred to a Schlenk flask containing 1 equiv. of corresponding chloroacetamide in EtOH, and the reaction mixture was refluxed under N_2 for 1h. It was then cooled to room temperature and stirred for another 12h. After which, the reaction mixture was dissolved in dichloromethane and washed with H_2O. The organic phase was then dried with brine then $MgSO_4$, concentrated and the resulting crude product was recrystallized from hexane or purified via silica column chromatography:

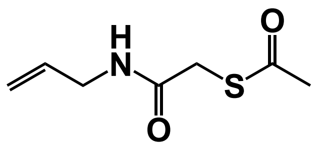

Dense yellow oil obtained purified via silica column chromatography (1:1 Hex/EtOAc) followed by solvent removal under vacuo, yield: 80%. ^{1}H-NMR (400, $CDCl_3$, 298 K): δ 6.35 (br, 1H), 5.83 – 5.73 (m, 1H), 5.15 – 5.09 (m, 2H), 3.82 (tt, 2H, $^3J_{HH}$ 5.7 Hz, $^4J_{HH}$ 1.45 Hz), 3.53 (s, 2H) and 2.38 (s, 3H).

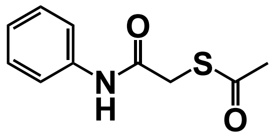

White crystalline solid obtained from recrystallization from hexane, yield: 73%. ^{1}H-NMR (400 MHz, $CDCl_3$, 298 K): δ 8.09 (br, 1H), 7.49 (dt, 2H, $^3J_{HH}$ 7.7 Hz, $^4J_{HH}$ 1.1 Hz), 7.32 (tt, 2H, $^3J_{HH}$ 7.7 Hz, $^4J_{HH}$ 1.9 Hz), 7.11 (tt, 2H, $^3J_{HH}$ 7.5 Hz, $^4J_{HH}$ 1.1 Hz), 3.66 (s, 2H) and 2.45 (s, 3H).

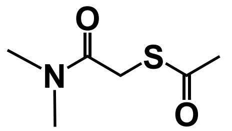

Dense yellow oil purified via silica column chromatography, yield: 60%. ^{1}H-NMR (400 MHz, $CDCl_3$, 298 K): δ 3.82 (s, 2H), 3.08 (s, 3H), 2.96 (s, 3H) and 2.36 (s, 3H).

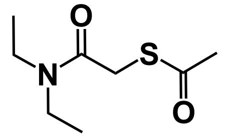

Dense yellow oil purified via silica column chromatography, yield: 63%. ^{1}H-NMR (400 MHz, $CDCl_3$, 298 K): δ 3.83 (s, 2H), 3.38 (qd, 2H, $^3J_{HH}$ 7.1 Hz, $^4J_{HH}$ 2.1 Hz), 2.37 (s, 3H), 1.23 (t, 2H, $^3J_{HH}$ 7.2 Hz) and 1.12 (t, 2H, $^3J_{HH}$ 7.2 Hz).

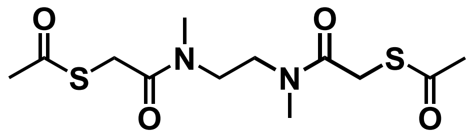

An off-white low melting-point solid obtained from silica column chromatography, yield 70 %. ^{1}H-NMR (400 MHz, $CDCl_3$, 298 K): δ 3.77 (s, 4H), 3.56 (s, 4H), 3.09 (s, 6H) and 2.34 (s, 6H).

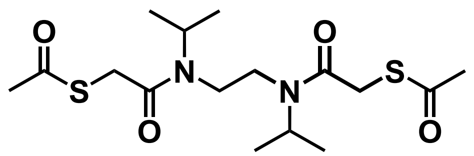

Off-white crystalline solid obtained from recrystallization from hexane, yield 65 %. ^{1}H-NMR (400 MHz, $CDCl_3$, 298 K): δ 4.00 (qu, 2H, $^3J_{HH}$ 6.7 Hz), 3.83 (s, 4H), 3.25 (s, 4H), 2.31 (s, 6H) and 1.22 (d, 12H, $^3J_{HH}$ 6.7 Hz).

General synthesis of *tethered* complexes 1^R (R = Me/^{i}Pr):

A mixture of 0.09 mmol of the S-acetylated ethylene-bis(N,N'-dialkyl-N,N'-mercaptoacetamide) ligand, 8 mg (0.2 mmol) of NaOH and 20 mg (0.1 mmol) of $Zn(OAc)_2.2H_2O$ was dissolved and stirred in 15mL of MeOH for 15 minutes. A 10mL portion of MeOH containing 50 mg (0.18 mmol) of $Mn(CO)_5Br$ was then added, and the resulting mixture was kept in the dark and heated at 60°C. The reaction reached completion after 3h, upon which the solvent was

evaporated under reduced pressure and the resulting yellow residue was dissolved in THF. The yellow THF solution was then filtered through Celite, and the solvent was evaporated in vacuo to yield a yellow power. XRD quality crystals were obtained from THF/pentane vapor diffusion. Spectroscopic yield: 80 – 90 %.

1^{Me}: IR in THF (ν_{CO}, cm^{-1}): 2025 (w), 2006 (s), 1913 (s, br) and 1568 (m, amide C=O).

1^{iPr}: IR in THF (ν_{CO}, cm^{-1}): 2025 (w), 2006 (s), 1913 (s, br) and 1568 (m, amide C=O). ^{1}H – NMR (400 MHz, DMSO-d$_6$, 298 K): δ 4.25 (sept, 2H, $^{3}J_{HH}$ 6.6 Hz), 4.18 (dd, 2H, $^{2}J_{HH}$ 14.4 Hz, $^{3}J_{HH}$ 4.4 Hz), 3.45 (d, 2H, $^{2}J_{HH}$ 14.5 Hz), 3.37 (dd, 2H, $^{2}J_{HH}$ 14.4 Hz, $^{3}J_{HH}$ 4.4 Hz), 3.12 (d, 2H, $^{2}J_{HH}$ 14.5 Hz), 1.39 (d, 6H, $^{3}J_{HH}$ 6.6 Hz) and 1.17 (d, 6H, $^{3}J_{HH}$ 6.6 Hz).

Synthesis of compound 1^{R}-Red:

To a THF solution containing 0.05 mmol of **1*^{iPr}*** and 38 mg (0.1 mmol) of 2,2,2-cryptand, 27 mg (0.2 mmol) of KC_8 was added. The solution color immediately changed from bright yellow to dark red. After 10 minutes, the solution was filtered through Celite to afford a bright red colored solution. More product was collected by passing dry MeCN through the Celite column. XRD quality crystals were obtained from THF/diethyl ether or MeCN/diethyl ether layer diffusion. IR in THF (ν_{CO}, cm^{-1}): 1938 (m), 1881 (s), 1827 (m), 1809 (m), 1794 (sh) and 1624 (w, amide C=O).

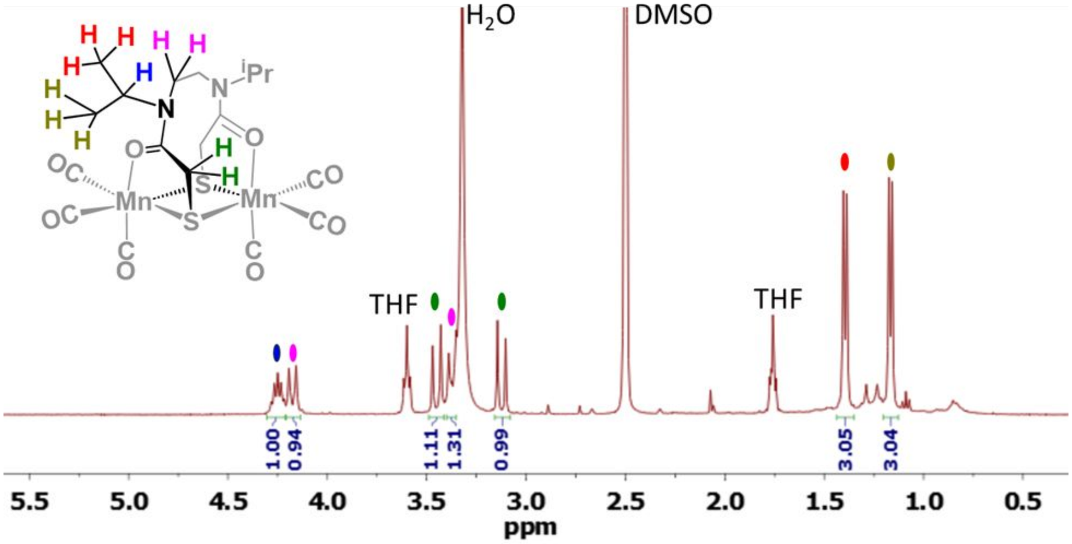

Figure S1. ^{1}H-NMR spectrum of **1*^{iPr}*** in DMSO-d$_6$ at 298 K. Assignments of the ethylene protons (pink ovals) were suggested by the COSY – NMR spectrum of **1^{iPr}** in DMSO-d$_6$.

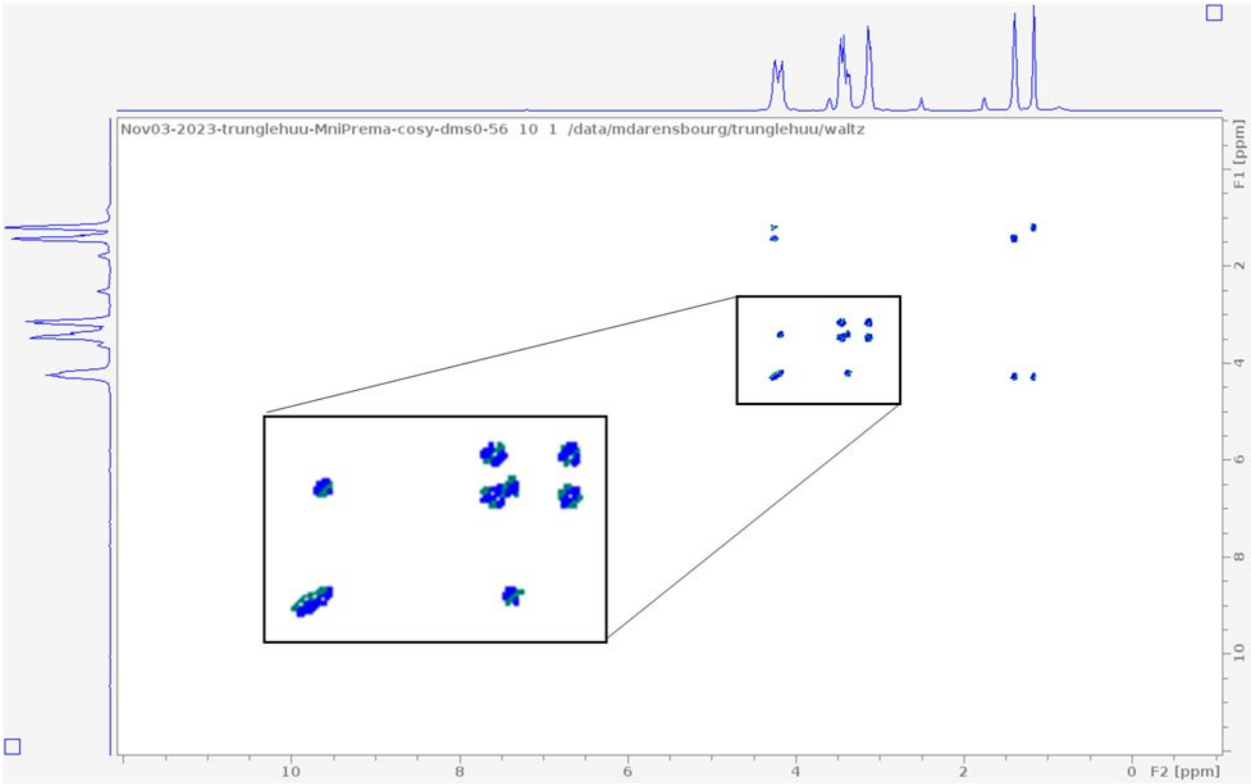

Figure S2. COSY-NMR spectrum of $\mathbf{1}^{\mathbf{iPr}}$ indicating coupling of the doublets at 4.18 ppm and 3.37 ppm.

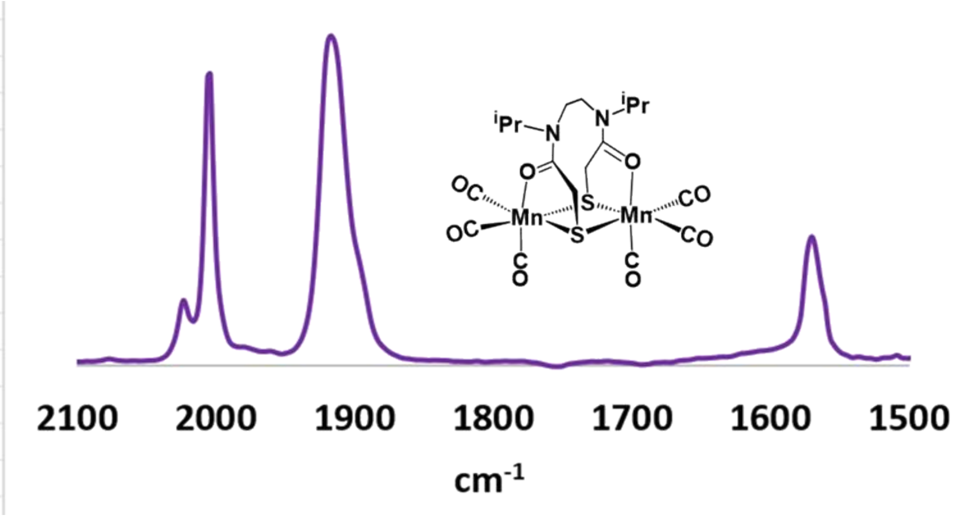

Figure S3. FT-IR spectrum of $\mathbf{1}^{\mathbf{iPr}}$ in THF.

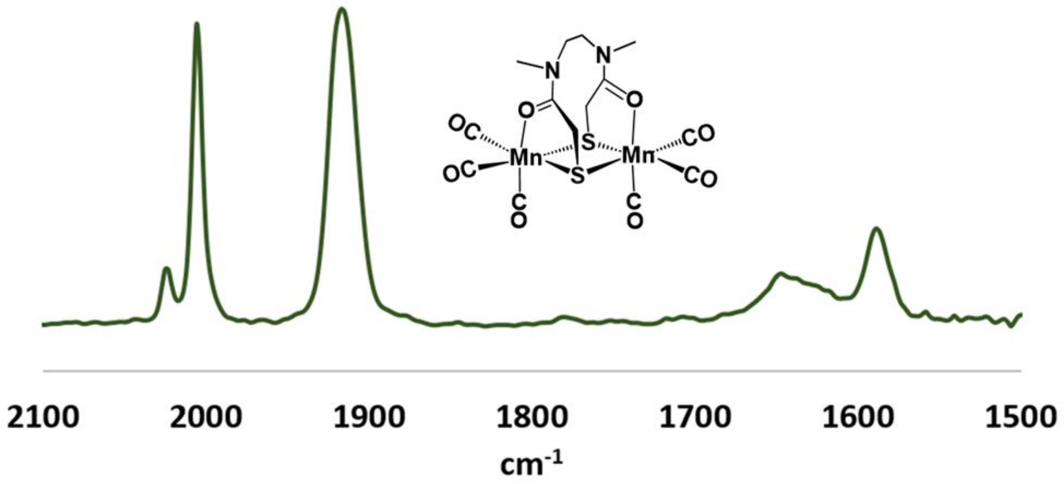

Figure S4. FT-IR spectrum of $\mathbf{1^{Me}}$ in THF.

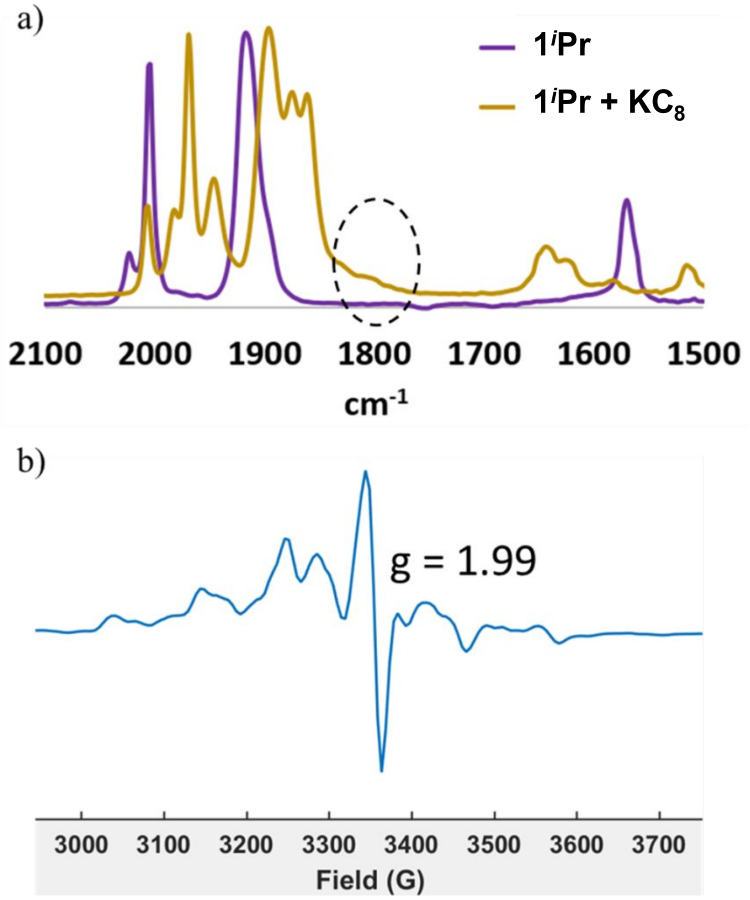

Figure S5. a) FT-IR monitor of the reaction of $\mathbf{1^{\mathit{i}}Pr}$ with KC_8; b) EPR spectrum of $\mathbf{1^{\mathit{i}Pr}}$ collected at 4K.

Synthesis of $[Mn_2HE]_2^{2-}$, $[2^{Me}-2H^+]^{2-}$: To a THF solution containing 20 mg (0.04 mmol) of $\mathbf{2^{Me}}$ and 25 mg (0.09 mmol) of 18-c-6, 11 mg (0.08 mmol) of KC_8 was added and stirred at room temperature. The reaction reached completion after 15 minutes as monitored by FT-IR. The solution was then filtered through Celite, and the resulting orange-yellow solution was layered with diethyl ether for crystallization. IR in THF (ν_{CO}, cm^{-1}): 1977 (s), 1880 (s), and 1568 (m, amide C=O).

*Note: $[2^{Me}-2H^+]^{2-}$ can also be obtained from the reaction of $\mathbf{2^{Me}}$ excess amount of strong base either in THF for FT-IR monitor or DMSO-d_6 for NMR. ^{1}H-NMR (400 MHz, DMSO, 298K): 3.31 (s, 3H), 2.89 (d, $^2J_{HH}$ 15.6 Hz, 1H) and 2.68 d, $^2J_{HH}$ 15.6 Hz, 1H).

Synthesis of $[2^{Me}-H^+]^-$: To a THF solution containing 15 mg (0.03 mmol) of $\mathbf{2^{Me}}$ and 10 mg (0.04 mmol) of 18-c-6, 4 mg (0.035 mmol) of KO^tBu was slowly added and stirred at room temperature. The reaction reached completion after 5 minutes as monitored by FT-IR. The resulting yellow solution was filtered to Celite and layered with diethyl ether for crystallization. FT-IR in THF (ν_{CO}, cm^{-1}): 2010 (w) 1990 (s), 1890 (s, br), 1614 (m, amide C=O) and 1574 (m, amide C=O).

General synthesis for derivatives of Complexes 2 and 3:

A mixture 0.18 mmol of the corresponding thioester acetamide and 7 mg of NaOH (0.18 mmol) in 15 mL of MeOH stirred for 15 mins under N_2. 50 mg (0.18 mmol) of $Mn(CO)_5Br$ dissolved in 10 mL of MeOH was then added. The reaction mixture was stirred in dark at room temperature. After reaching completion, as evident from IR spectroscopy, the solvent was removed in vacuo. The resulting orange-yellow residue was then dissolved in THF, filtered through Celite and purified through a short alumina column. The solution volume was then reduced, and precipitation via the addition of hexane afforded the crude yellow powder product. X-ray quality crystals were obtained from pentane/THF vapor diffusion.

2^{allyl}: IR in THF (ν_{CO}, cm^{-1}): 2025 (w), 2003 (s), 1917 (s), 1902 (s) and 1608 (m, amide C=O);

2^{Ph}: IR in THF (ν_{CO}, cm^{-1}): 2027 (w), 2007 (s), 1921 (s), 1907 (s), 1612 (m, aromatic C=C), 1598 (m, amide C=O) and 1562 (m, aromatic C=C). ^{1}H – NMR (400 MHz, DMSO-d_6, 298 K): δ 11.70 (s, 1H), 11.00 (s, 1H), 7.66 (s, 1H), 7.65 (s, 1H), 7.49 (t, 2H), 7.28 – 7.22 (m, 3H), 7.17 – 7.10 (m, 3H), 3.73 (dd, 2H) and 3.36 (dd, 2H).

3^{Me}: IR in THF (ν_{CO}, cm^{-1}): 2025 (w), 2004 (s), 1917 (s), 1899 (s), and 1597 (m, amide C=O).

3^{Et}: IR in THF (ν_{CO}, cm^{-1}): 2025 (w), 2004 (s), 1915 (s), 1900 (s), and 1597 (m, amide C=O).

Synthesis of $[2^{allyl}-2H^+]^{2-}$: To a THF solution containing 31 mg (0.06 mmol) of $\mathbf{2^{allyl}}$, 10 mg (0.25 mmol) of 60 % w/w NaH dispersed in mineral oil was added and let stirred at room temperature. After 5 minutes, the mixture is filtered through Celite and 45 mg (0.12 mmol) of 2,2,2-cryptand was added. The resulting solution was layered with diethyl ether for crystallization. IR in THF

(ν_{CO}, cm^{-1}): 1977 (s), 1880 (s), and 1568 (m, amide C=O). ^{1}H – NMR (400 MHz, DMSO-d_6, 298 K): δ 5.99 (m, 1H), 5.05 (d, 1H), 4.91 (d, 1H), 4.57 (dd, 1H), 3.73 (dd, 1H), 2.87 (d, 1H) and 2.71 (d, 1H).

Synthesis of $[2^{Ph}-2H^{+}]^{2-}$: To a THF solution containing 37 mg (0.06 mmol) of **2^{Ph}**, 10 mg (0.25 mmol) of 60 % w/w NaH dispersed in mineral oil was added and stirred at room temperature. After 5 min, the mixture was filtered through Celite and 24 µL (0.12 mmol) of 15-c-5 was added. The resulting solution was layered with diethyl ether for crystallization. IR in THF (ν_{CO}, cm^{-1}): 1985 (s), 1890 (s) and 1564 (m, br, amide C=O).). ^{1}H – NMR (400 MHz, DMSO-d_6, 298 K): δ 7.26 (dt, 2H, $^{3}J_{HH}$ 7.1 Hz, $^{4}J_{HH}$ 1.2 Hz, $^{5}J_{HH}$ 1.9 Hz), 7.16 (tt, 2H, $^{3}J_{HH}$ 7.1 Hz, $^{5}J_{HH}$ 1.9 Hz), 6.87 (tt, 1H, $^{3}J_{HH}$ 7.1 Hz, $^{4}J_{HH}$ 1.2 Hz), 2.99 (d, 1H, $^{2}J_{HH}$ 16.0 Hz), 2.91 (d, 1H, $^{2}J_{HH}$ 16.0 Hz).

Synthesis of $[3^{Et}]^{2-}$, 3^{Et}-Red: To a THF solution containing 0.05 mmol of **4** and 38 mg (0.1 mmol) of 2,2,2-cryptand, 27 mg (0.2 mmol) of KC_8 was added. The solution color immediately changed from bright yellow to dark red. After 10 minutes, the solution was filtered through Celite to afford a bright red colored solution. More product was collected by passing dry MeCN through the Celite column. XRD quality crystals were obtained from THF/diethyl ether or MeCN/diethyl ether layer diffusion. IR in THF (ν_{CO}, cm^{-1}): 1938 (m), 1881 (s), 1827 (m), 1809 (m), 1794 (sh) and 1624 (w, amide C=O).

II. Formation of $[Mn_2HE]^{2-}$, compound 2^{Me}-Red:

Path A: To a THF solution containing 22 mg (0.045 mmol) of **2^{Me}**, **$[MnHE]_2$** and 26 mg of 18-c-6 (0.1 mmol), 27 mg (0.2 mmol) of KC_8 was added. Over the course of the reaction, the solution color quickly changed from bright yellow to orange and finally deep red. Depending on how much excess KC_8 is being added, the reaction time ranges from 30 mins to several hours. When the reaction reached completion as monitored by FTIR, the deep red solution was filtered through Celite. Crystallization via ether/THF layer diffusion afforded red crystals that were suitable for X-ray diffraction. The yield of the product is dependent on the amount of excess KC_8 with spectroscopic yield as high as 80 %. (ν_{CO}, cm^{-1}): 1944 (m), 1886 (s), 1832 (m), 1813 (m), and 1796 (sh); ESI-HRMS(−) m/z: 386.81 $[M+H]^{-}$.

Slight modification was applied as follows to analyze the headspace of the reaction vessel to determine the formation of H_2: Compound, 18-c-6 and KC_8 was placed in a 3-dram vial in a glovebox. The vial was then tightly secure with a rubber septum and placed under vacuo. THF

solvent (5mL) was added via syringe. The reaction was stirred for at least 30 mins and the headspace was subjected to GC analysis.

Path B: A THF solution containing 4 mg (0.035 mmol) of KO^tBu was added dropwise to a 4 mL THF solution containing 15 mg (0.03 mmol) of $\mathbf{2^{Me}}$**.** Upon reaching the singly deprotonated species $[\mathbf{2^{Me}}-H^{+}]^{-}$ evident from FT-IR, 16 mg (0.06 mmol) of 18-c-6 was added followed by 16 mg (0.12 mmol) of KC_8. Similar work-up procedure to path A was then employed. Spectroscopic yield: 35 %.

IR Monitoring of the Reduction of 2^{Me} .

The reaction was monitored via FT-IR in the diatomic ν(CO) region, which, over the course of 30 min, displayed a drastic change in band pattern and position, Figure S6-a. While the red-shift of the ν(CO) bands, of *ca.* 80 cm^{-1}, confirms the increased electron density around the metal centers resulted from chemical reduction, the change in pattern suggests a significant difference in the positions and electronic environment of CO ligands. Mass spectrometry analysis in the ESI (−) mode found a single main species at m/z = 381.86 that corresponds to a chemical formula of the original compound $\mathbf{2^{Me}}$ minus a **HE** ligand, Figure S7.

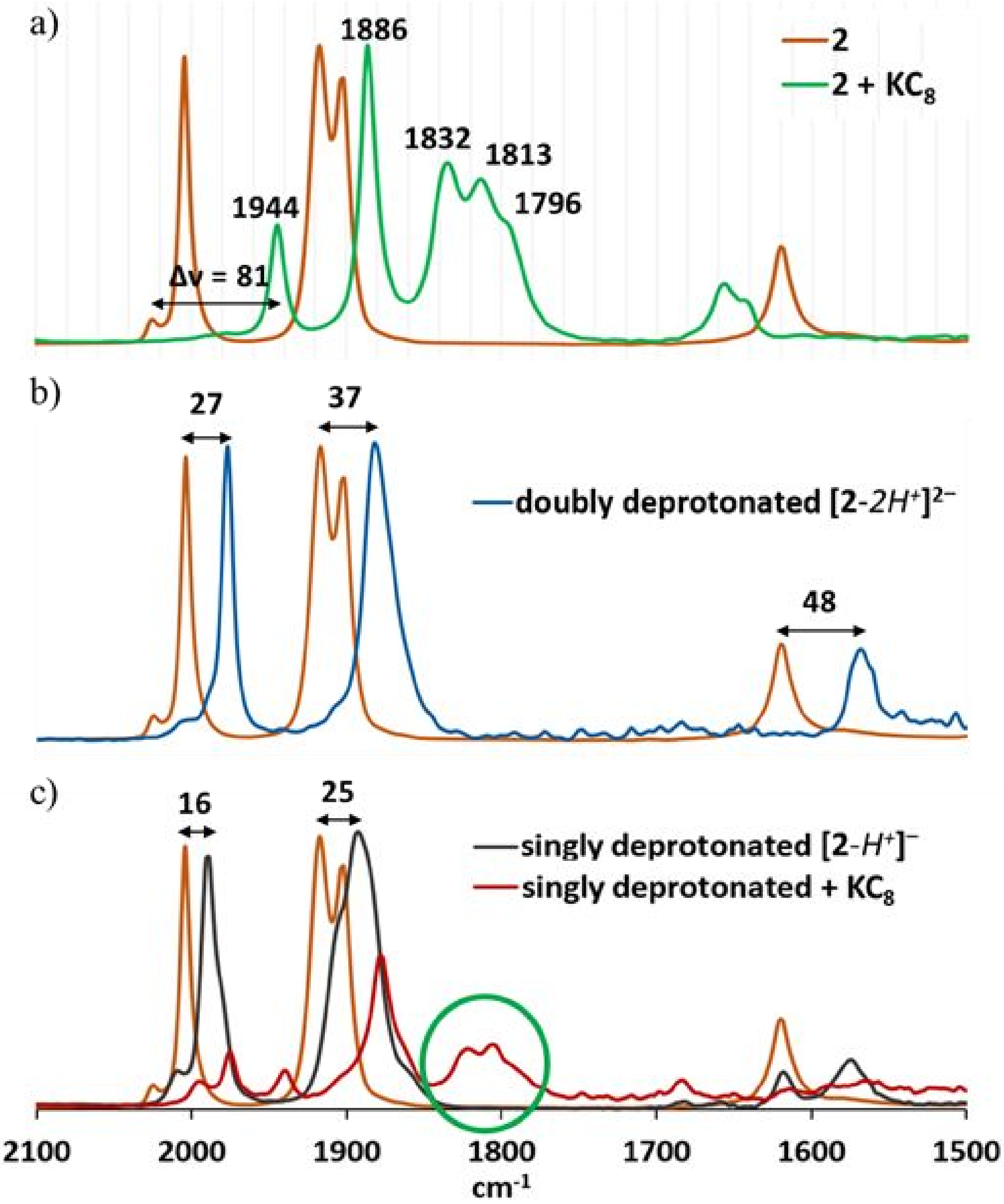

Figure S6. FT-IR monitor of reactions shown in **Scheme-2** a) Path A – reaction of $\mathbf{2}^{\mathbf{Me}}$ with excess KC_8; b) Path B – reaction of $\mathbf{2}^{\mathbf{Me}}$ with 2 equiv. of KC_8 or strong base; c) Path C – reaction of $\mathbf{2}^{\mathbf{Me}}$ with 1 equiv. of strong base followed by reaction with KC_8.

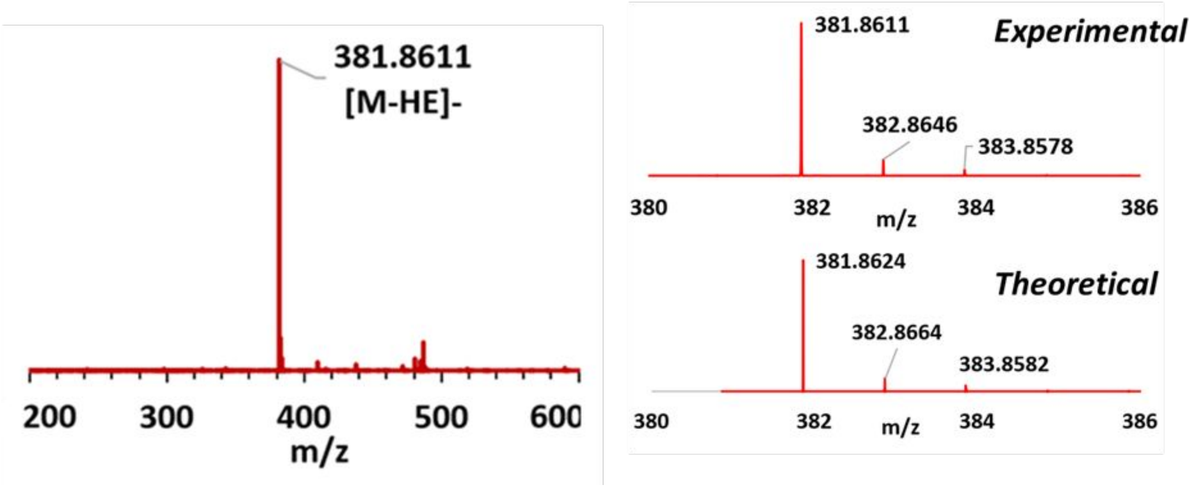

Figure S7. ESI (−) spectrum of the reaction mixture of $\mathbf{2^{Me}}$ with excess KC_8 displaying a single major m/z signal corresponding to $\mathbf{[2^{Me}-HE]^-}$**.**

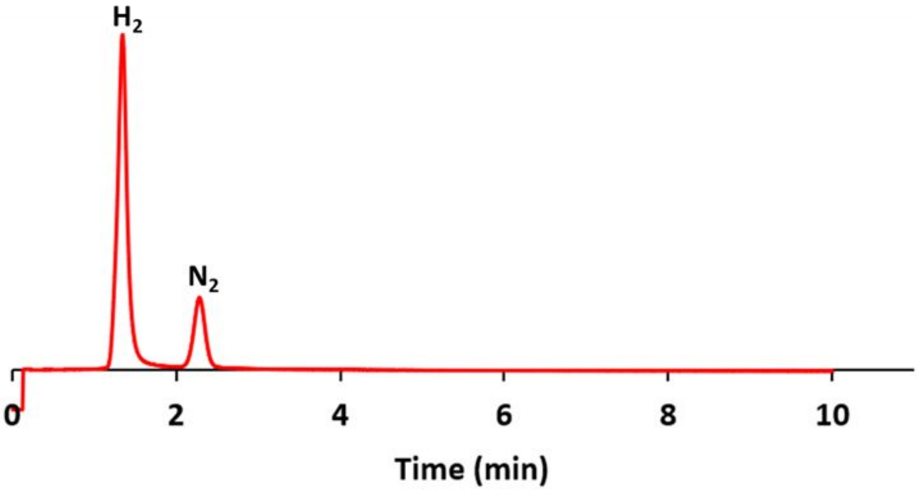

Figure S8. Headspace analysis of the reaction of $\mathbf{2^{Me}}$ with KC_8 via gas-chromatography indicating the formation of H_2 (detected at t = 1.4 min).

III. Characterizations:

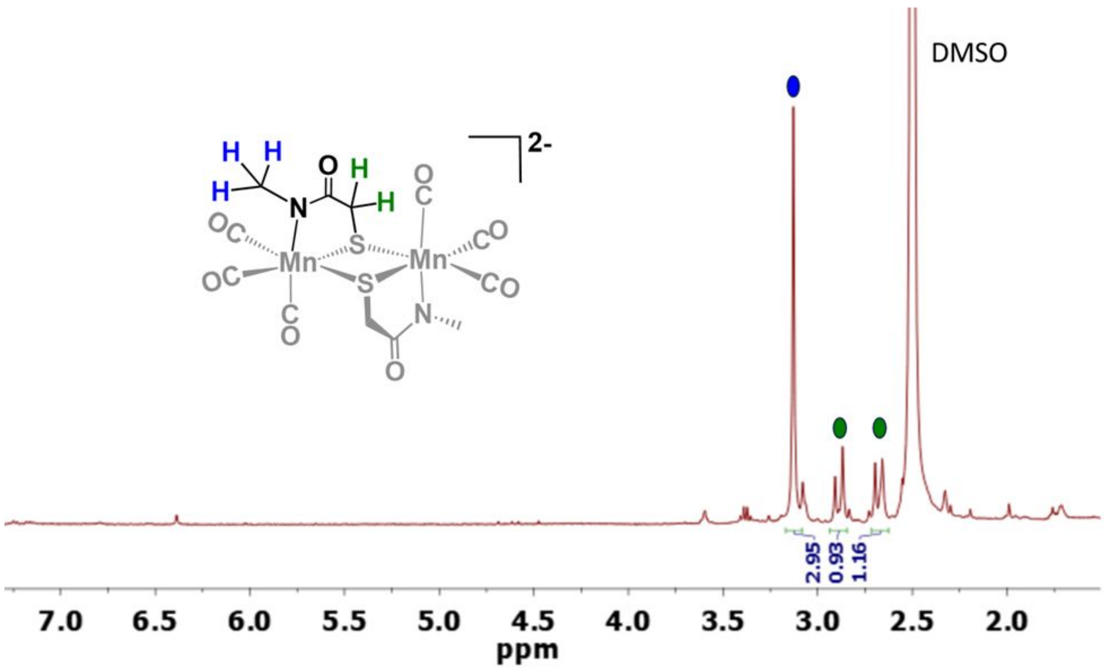

Figure S9. ^{1}H-NMR spectrum of **[2^{Me}−*2H*$^+$]$^{2-}$** with naked Na$^+$ counter cations in DMSO-d$_6$ at 298 K

1. Characterization of **2^{Allyl} - Red**:

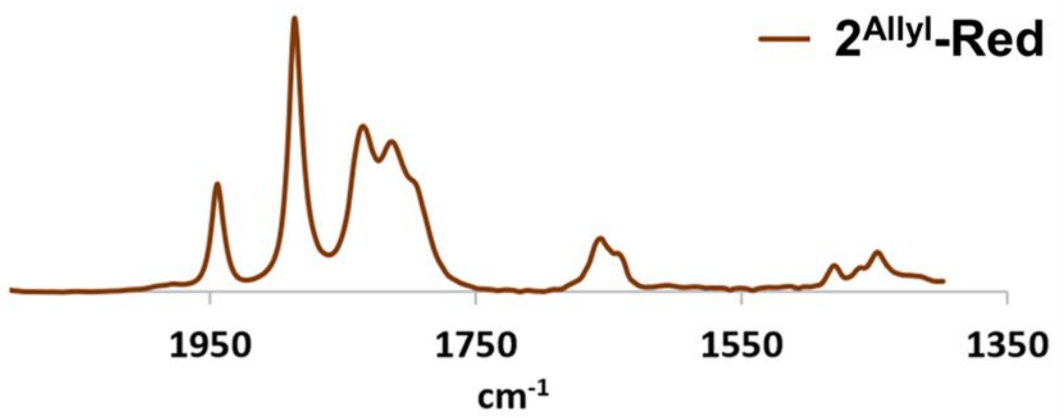

Figure S10. FT-IR of [**2^{allyl}-Red**][K(222-crypt)]$_2$ in CD$_3$CN.

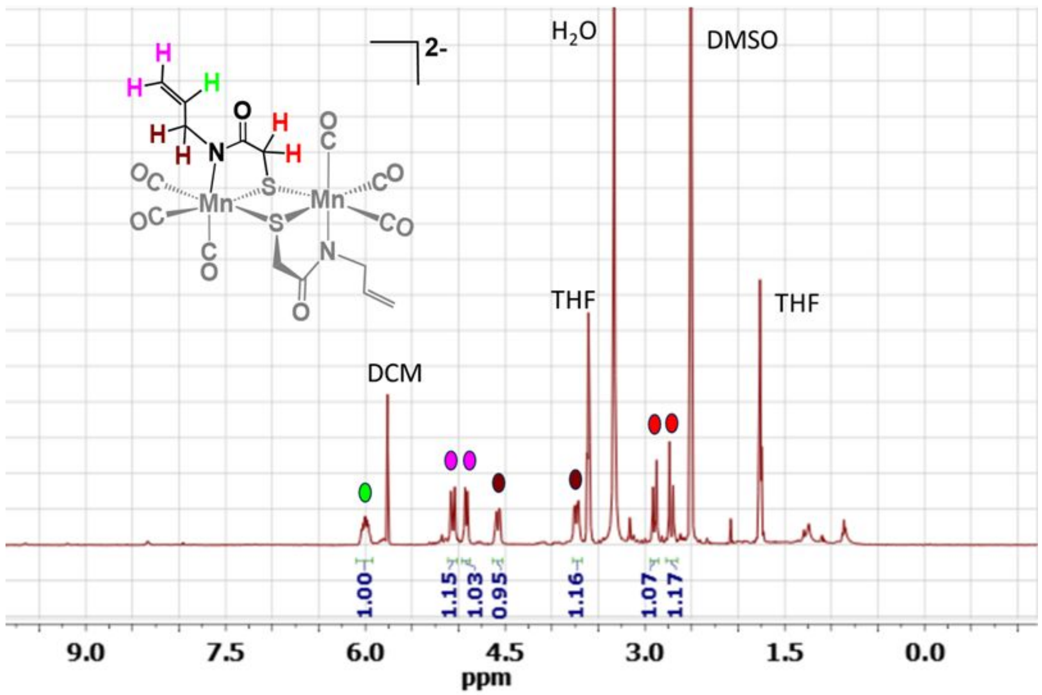

Figure S11. ^{1}H-NMR spectrum of **[2^{allyl}–*2H*$^{+}$]$^{2-}$** with naked Na^{+} counter cations in DMSO-d_6 at 298 K

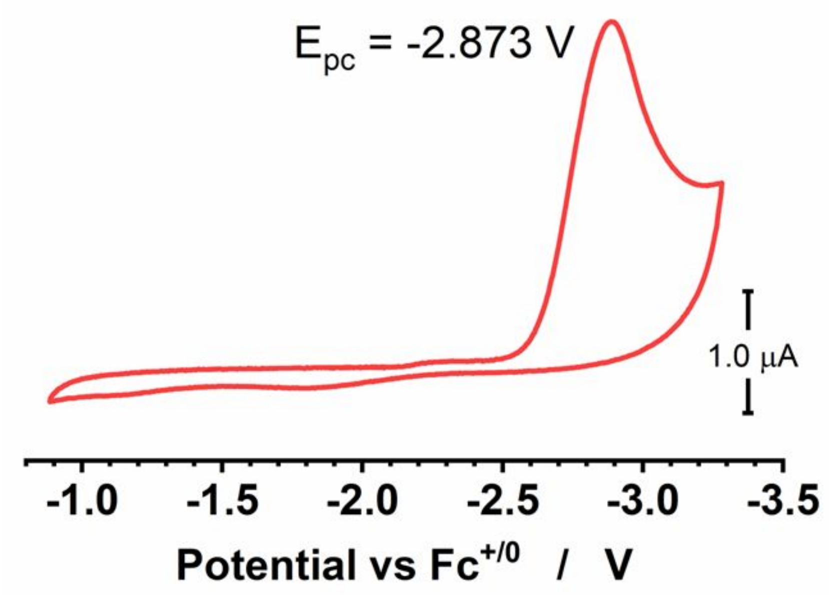

Figure S12. Cyclic voltammogram of 1 mM **[2^{Me}–*2H*$^{+}$]$^{2-}$** in THF (working electrode: glassy carbon; counter electrode: Pt wire; reference electrode: Ag pseudo reference; electrolyte: 100 mM $TBAPF_6$).

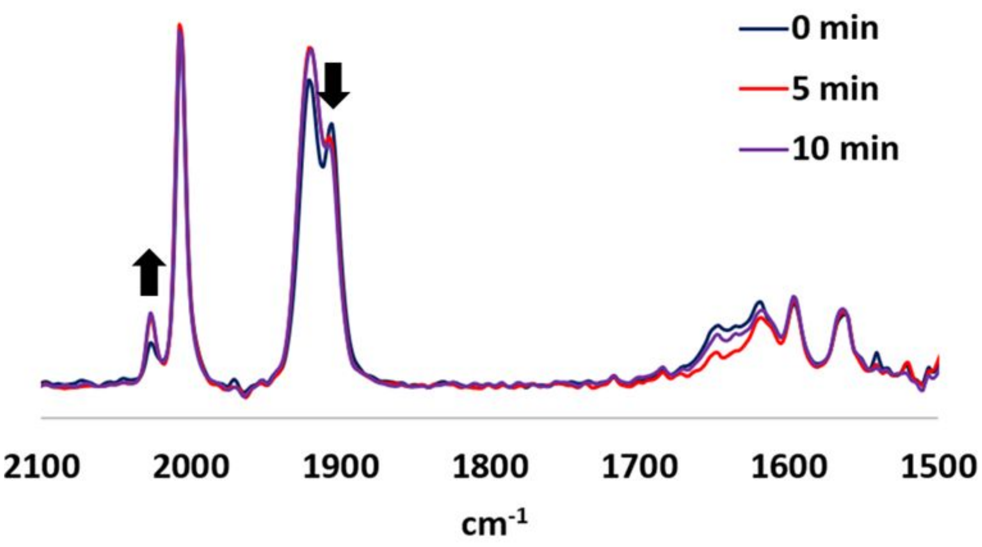

Figure S13. FT-IR of $\mathbf{2^{Ph}}$ in THF displaying similar isomerization between *syn*/*anti* configurations similar to $\mathbf{2^{Me}}$.

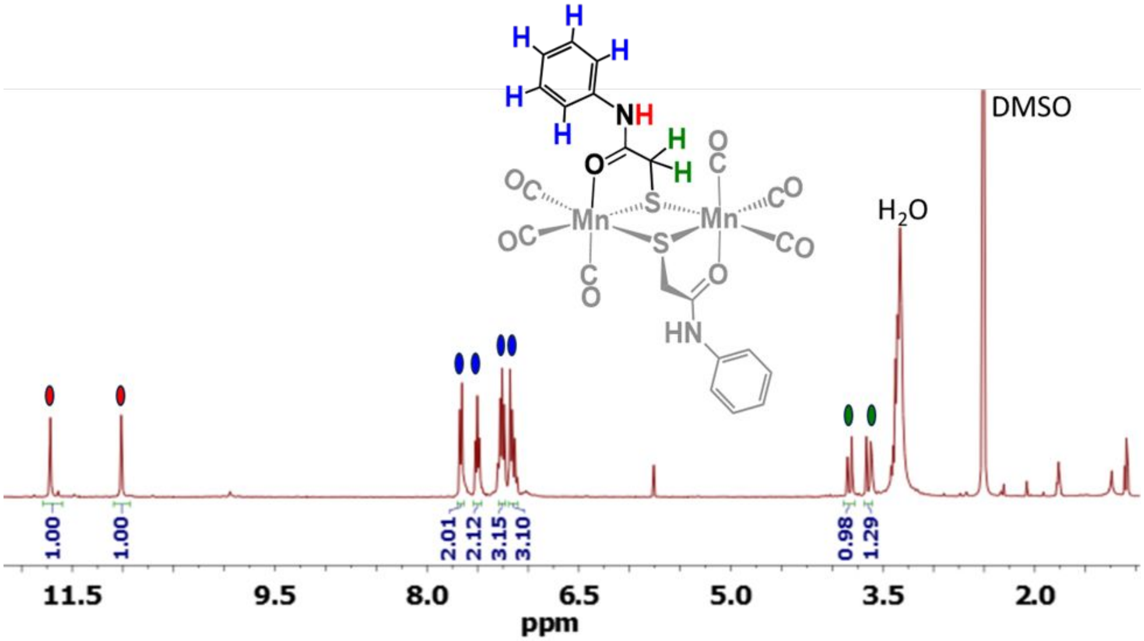

Figure S14. ^{1}H-NMR spectrum of $\mathbf{2^{Ph}}$ in DMSO-d_6 at 298 K.

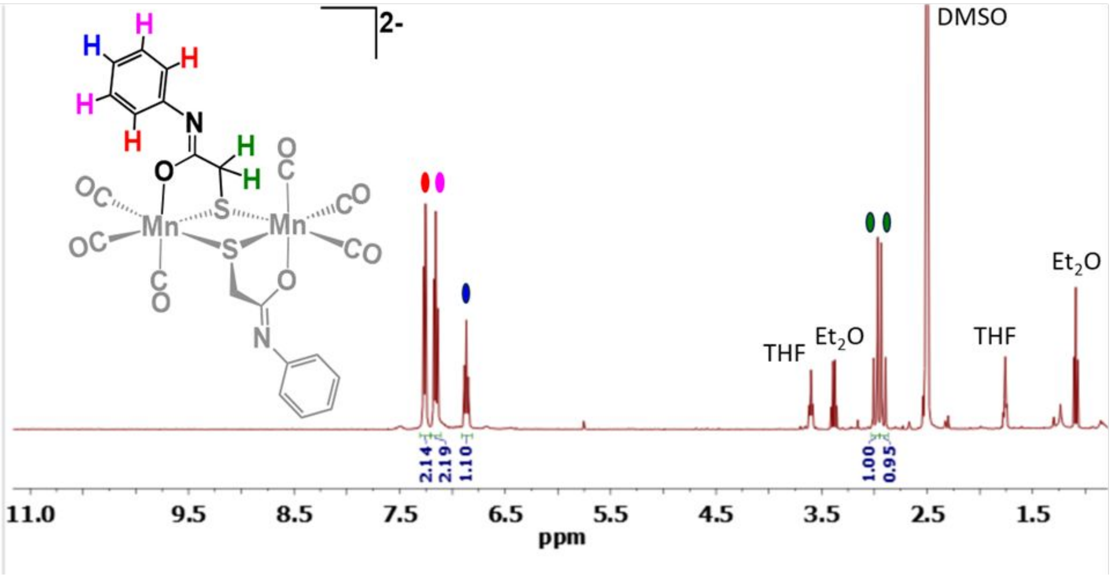

Figure S15. ^{1}H-NMR spectrum of **[2^{Ph}–*2H*$^{+}$]$^{2-}$.**

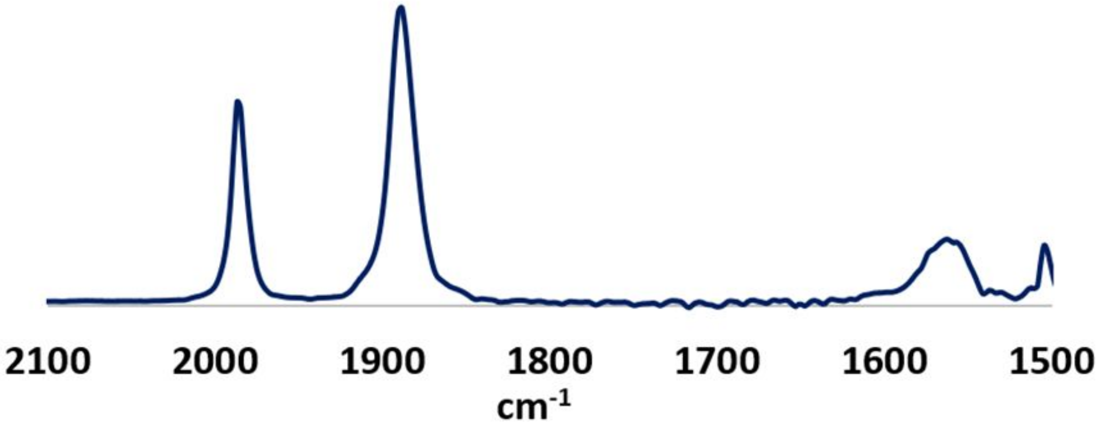

Figure S16. FT-IR spectrum of **[2^{Ph}–*2H*$^{+}$]$^{2-}$** in THF.

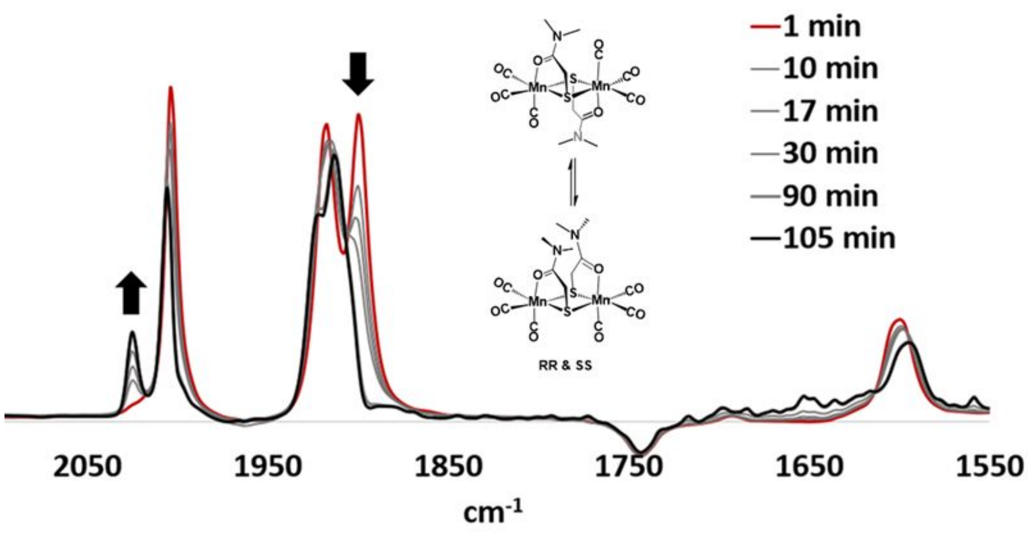

Figure S17. FT-IR monitor of dissolving crystals of **3^{Me}** into THF displaying an isomerization from the *anti*-isomer to a mixture of *anti*/*syn* isomers in solution.

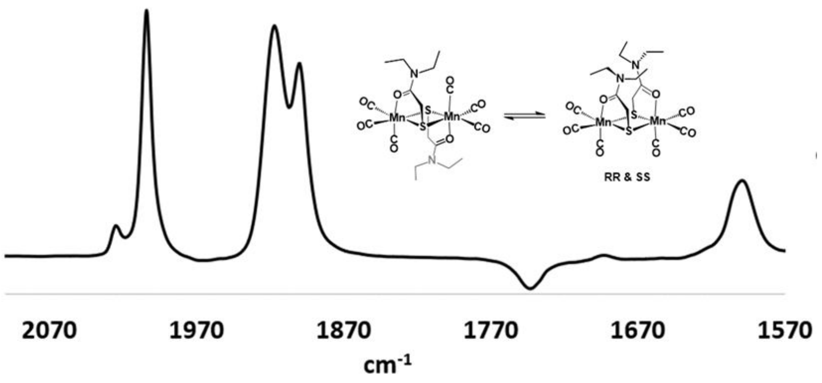

Figure S18. FT-IR spectrum of **3^{Et}** in THF at an equilibrium between the *anti* and *syn* isomers.

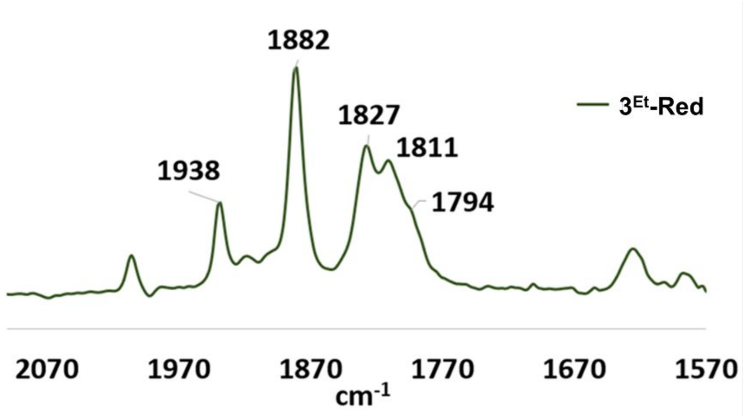

Figure S19. FT-IR spectrum of **[3^{Et}][**K(222-crypt)]$_2$ in CD_3CN.

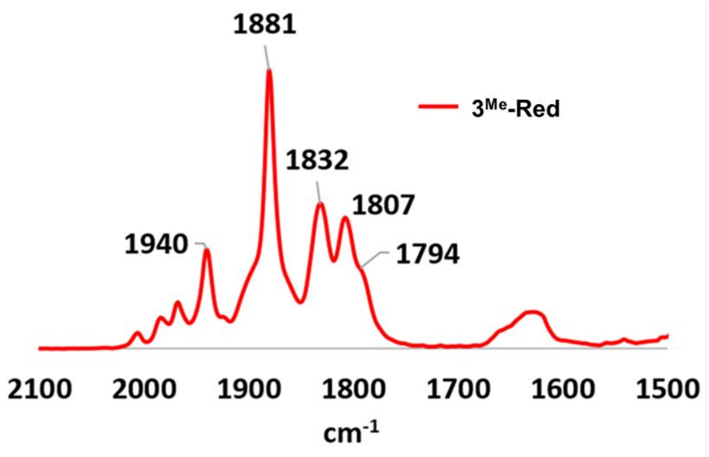

Figure S20. FT-IR spectrum of **[3^{Me}][**K(18-c-6)]$_2$ in THF.

IV. Crystallographic data:

Identification code	08102_MniPrema_0ma
Empirical formula	$C_{18}H_{22}Mn_2N_2O_8S_2$
Formula weight	568.37
Temperature/K	110.00
Crystal system	monoclinic
Space group	$P2_1/c$
a/Å	21.3323(9)
b/Å	18.5348(8)
c/Å	18.0553(8)
α/°	90
β/°	99.971(2)
γ/°	90
Volume/Å^3	7031.1(5)
Z	12
ρ_{calc}g/cm^3	1.611
μ/mm^{-1}	1.301
F(000)	3480.0
Crystal size/mm^3	0.1 × 0.1 × 0.05
Radiation	MoKα (λ = 0.71073)
2Θ range for data collection/°	3.878 to 60.124
Index ranges	$-30 \leq h \leq 30$, $-26 \leq k \leq 26$, $-25 \leq l \leq 25$
Reflections collected	260652
Independent reflections	20601 [R_{int} = 0.0707, R_{sigma} = 0.0345]
Data/restraints/parameters	20601/0/877
Goodness-of-fit on F^2	1.130
Final R indexes [I>=2σ (I)]	R_1 = 0.0327, wR_2 = 0.0646
Final R indexes [all data]	R_1 = 0.0613, wR_2 = 0.0799
Largest diff. peak/hole / e Å^{-3}	0.52/-0.64

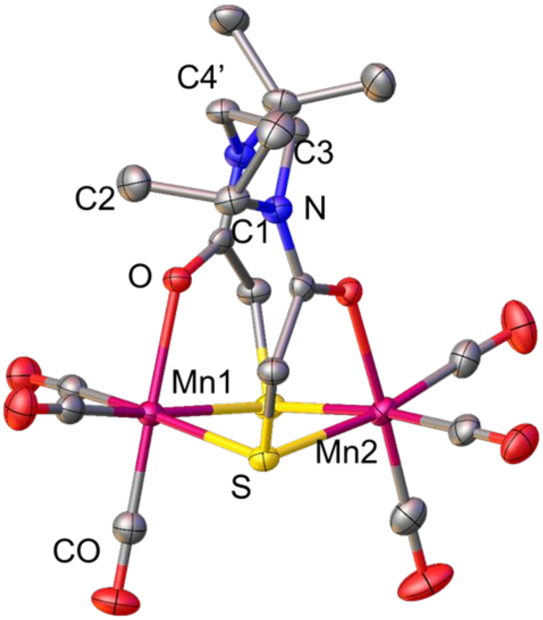

Figure S21. *Left* - Crystal data and structure refinement parameters of **1^{iPr}**; *Right* - Molecular structure of **RR** isomer of **1^{iPr}** with thermal ellipsoids plotted at 50 % probability; **SS** isomer is omitted for clarity.

Identification code	abcde_0ma
Empirical formula	$C_{16}H_{16}Mn_2N_2O_8S_2$
Formula weight	538.31
Temperature/K	110.0
Crystal system	monoclinic
Space group	$P2_1/c$
a/Å	9.666(3)
b/Å	10.100(3)
c/Å	10.935(3)
α/°	90
β/°	99.798(8)
γ/°	90
Volume/Å^3	1052.0(5)
Z	2
ρ_{calc}g/cm^3	1.699
μ/mm^{-1}	1.444
F(000)	544.0
Crystal size/mm^3	0.2 × 0.1 × 0.1
Radiation	MoKα (λ = 0.71073)
2Θ range for data collection/°	4.276 to 61.744
Index ranges	$-13 \leq h \leq 13$, $-14 \leq k \leq 14$, $-15 \leq l \leq 15$
Reflections collected	41302
Independent reflections	3265 [R_{int} = 0.1374, R_{sigma} = 0.0711]
Data/restraints/parameters	3265/0/144
Goodness-of-fit on F^2	1.089
Final R indexes [I>=2σ (I)]	R_1 = 0.0827, wR_2 = 0.2301
Final R indexes [all data]	R_1 = 0.1366, wR_2 = 0.2723
Largest diff. peak/hole / e Å^{-3}	1.86/-1.28

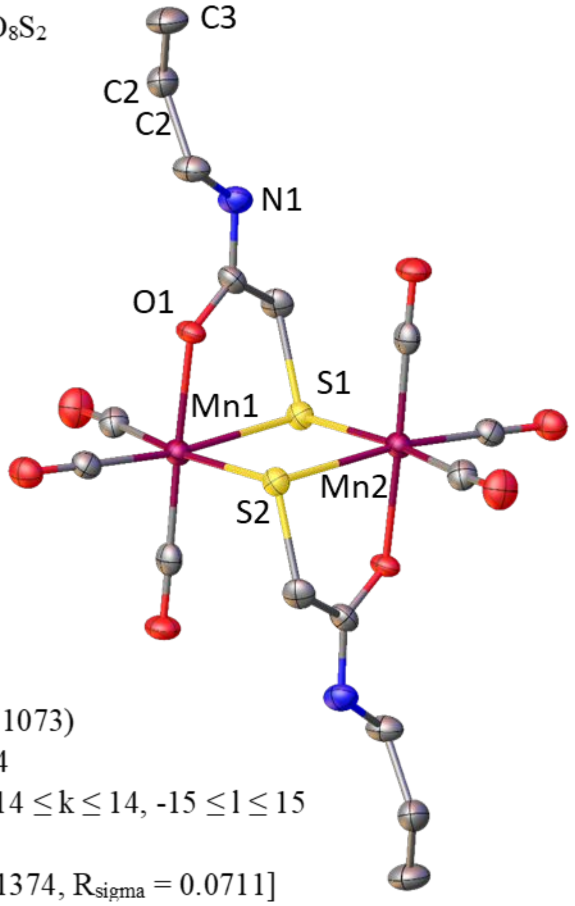

Figure S22. *Left* - Crystal data and structure refinement parameters of $\mathbf{2}^{\mathbf{allyl}}$; *Right* - Molecular structure of $\mathbf{2}^{\mathbf{allyl}}$ with thermal ellipsoids plotted at 50 % probability; H atoms omitted for clarity.

Identification code	CV_MnHEPh_needle_0m
Empirical formula	$C_{30}H_{32}Mn_2N_2O_{10}S_2$
Formula weight	754.57
Temperature/K	110.0
Crystal system	triclinic
Space group	P-1
a/Å	7.4830(15)
b/Å	9.4938(18)
c/Å	12.280(3)
α/°	75.278(9)
β/°	76.616(9)
γ/°	84.616(8)
Volume/Å^3	820.3(3)
Z	1
ρ_{calc}g/cm^3	1.527
μ/mm^{-1}	0.955
F(000)	388.0
Crystal size/mm^3	0.2 × 0.1 × 0.1
Radiation	MoKα (λ = 0.71073)
2Θ range for data collection/°	5.6 to 60.668
Index ranges	-10 ≤ h ≤ 10, -13 ≤ k ≤ 13, -17 ≤ l ≤ 17
Reflections collected	33816
Independent reflections	4915 [R_{int} = 0.1254, R_{sigma} = 0.0915]
Data/restraints/parameters	4915/0/208
Goodness-of-fit on F^2	1.046
Final R indexes [I>=2σ (I)]	R_1 = 0.0514, wR_2 = 0.0988
Final R indexes [all data]	R_1 = 0.1172, wR_2 = 0.1322
Largest diff. peak/hole / e Å^{-3}	0.55/-0.59

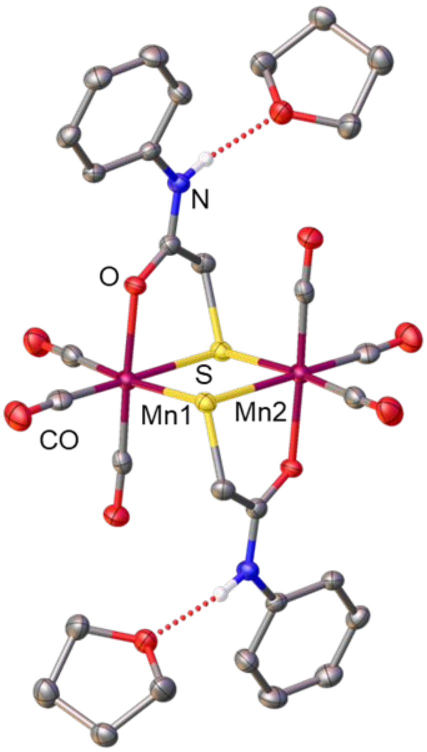

Figure S23. *Left* - Crystal data and structure refinement parameters of **2^{Ph}**; *Right* - Molecular structure of **2^{Ph}** with thermal ellipsoids plotted at 50 % probability; [K(18-c-6)]$^+$ moieties omitted for clarity.

Identification code	MnHE_KC8_second_0m
Empirical formula	$C_{37}H_{45.5}K_2Mn_2NO_{20}S$
Formula weight	1044.38
Temperature/K	110.0
Crystal system	triclinic
Space group	P-1
a/Å	10.0403(13)
b/Å	14.5200(18)
c/Å	33.479(4)
α/°	89.862(4)
β/°	84.755(4)
γ/°	89.996(4)
Volume/Å^3	4860.2(11)
Z	4
ρ_{calc}g/cm^3	1.427
μ/mm^{-1}	0.806
F(000)	2154.0
Crystal size/mm^3	0.2 × 0.1 × 0.1
Radiation	MoKα (λ = 0.71073)
2Θ range for data collection/°	3.666 to 60.296
Index ranges	-14 ≤ h ≤ 14, -20 ≤ k ≤ 20, -47 ≤ l ≤ 47
Reflections collected	302520
Independent reflections	28620 [R_{int} = 0.1300, R_{sigma} = 0.0820]
Data/restraints/parameters	28620/0/1146
Goodness-of-fit on F^2	1.044
Final R indexes [I>=2σ (I)]	R_1 = 0.0864, wR_2 = 0.2061
Final R indexes [all data]	R_1 = 0.1528, wR_2 = 0.2445
Largest diff. peak/hole / e Å^{-3}	2.15/-0.71

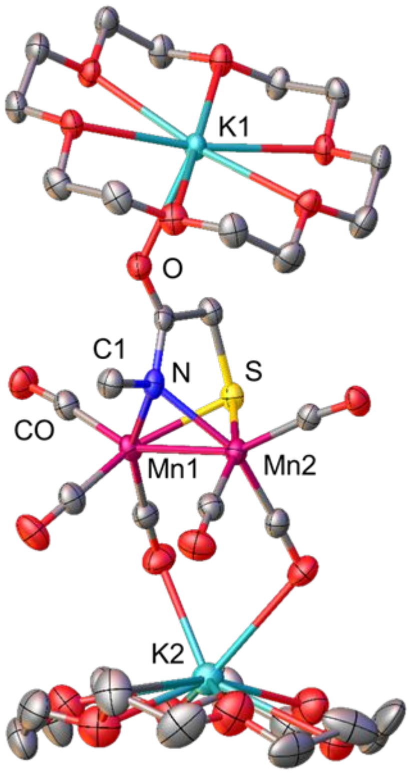

Figure S24. *Left* - Crystal data and structure refinement parameters of **[2^{Me}-Red]**[K(18-c-6)]$_2$; *Right* - Molecular structure of **[2^{Me}-Red]**[K(18-c-6)]$_2$ with thermal ellipsoids plotted at 50 % probability.

Identification code	Mn_allylHE_KC8_0ma
Empirical formula	$C_{39}H_{63}K_2Mn_2NO_{20}S$
Formula weight	1086.04
Temperature/K	110.0
Crystal system	monoclinic
Space group	$P2_1/c$
a/Å	9.854(3)
b/Å	14.450(4)
c/Å	35.538(9)
α/°	90
β/°	90.393(15)
γ/°	90
Volume/Å^3	5060(2)
Z	4
ρ_{calc}g/cm^3	1.426
μ/mm^{-1}	6.543
F(000)	2272.0
Crystal size/mm^3	0.2 × 0.1 × 0.05
Radiation	CuKα (λ = 1.54178)
2Θ range for data collection/°	4.974 to 138.238
Index ranges	$-11 \leq h \leq 11$, $-17 \leq k \leq 17$, $-42 \leq l \leq 41$
Reflections collected	127471
Independent reflections	9331 [R_{int} = 0.1041, R_{sigma} = 0.0410]
Data/restraints/parameters	9331/81/604
Goodness-of-fit on F^2	1.027
Final R indexes [I>=2σ (I)]	R_1 = 0.0494, wR_2 = 0.1210
Final R indexes [all data]	R_1 = 0.0720, wR_2 = 0.1368
Largest diff. peak/hole / e Å^{-3}	1.02/-0.40

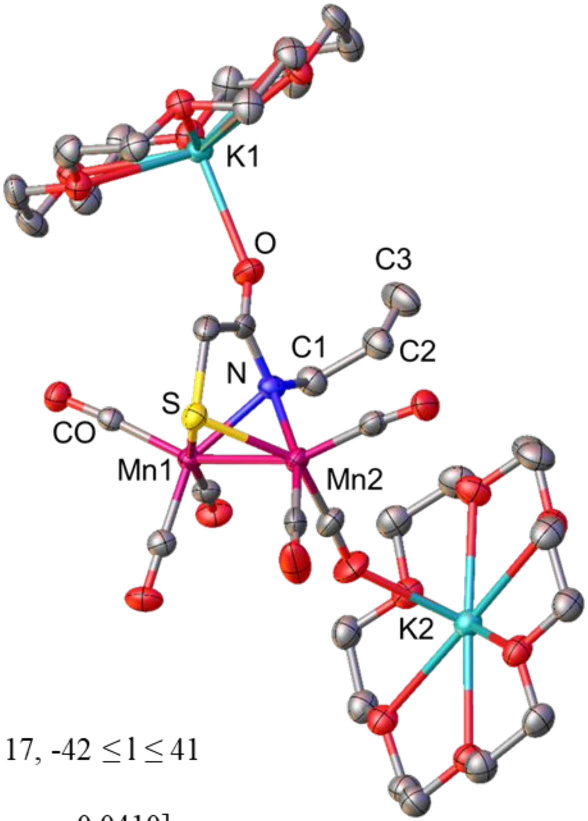

Figure S25. *Left* - Crystal data and structure refinement parameters of **[2^{allyl}-Red]**[K(18-c-6)]$_2$; *Right* - Molecular structure of **[2^{allyl}-Red]**[K(18-c-6)]$_2$ with thermal ellipsoids plotted at 50% probability.

Identification code	MnZ4_0ma	
Empirical formula	$C_{36}H_{55}K_2Mn_2N_2O_{20}S_2$	
Formula weight	1088.02	
Temperature/K	110.0	
Crystal system	monoclinic	
Space group	$P2_1/c$	
a/Å	24.565(5)	
b/Å	8.3519(16)	
c/Å	23.632(4)	
α/°	90	
β/°	96.388(6)	
γ/°	90	
Volume/Å^3	4818.3(16)	
Z	4	
ρ_{calc}g/cm^3	1.500	
μ/mm^{-1}	0.859	
F(000)	2260.0	
Crystal size/mm^3	0.2 × 0.2 × 0.1	
Radiation	MoKα (λ = 0.71073)	
2Θ range for data collection/°	3.468 to 59.304	
Index ranges	$-34 \leq h \leq 34$, $-11 \leq k \leq 11$, $-32 \leq l \leq 32$	
Reflections collected	170646	
Independent reflections	13565 [R_{int} = 0.0861, R_{sigma} = 0.0435]	
Data/restraints/parameters	13565/0/579	
Goodness-of-fit on F^2	1.071	
Final R indexes [I>=2σ (I)]	R_1 = 0.0423, wR_2 = 0.0923	
Final R indexes [all data]	R_1 = 0.0771, wR_2 = 0.1093	
Largest diff. peak/hole / e Å^{-3}	0.83/-0.76	

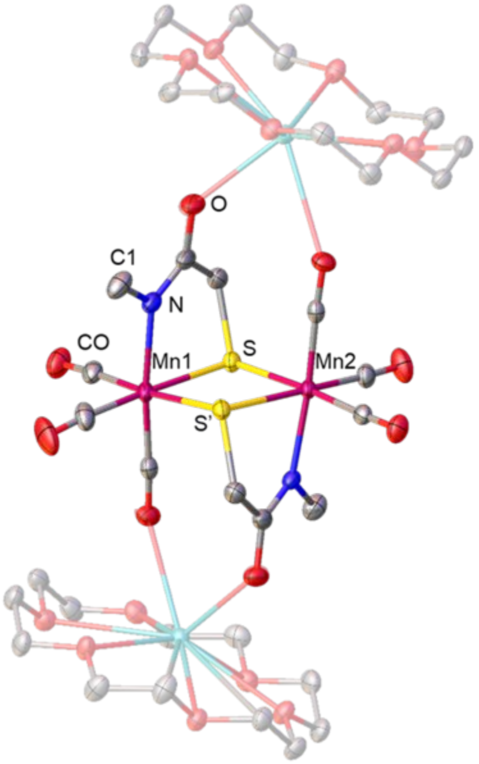

Figure S26. *Left* - Crystal data and structure refinement parameters of **[2^{Me}−*2H*$^+$]**[K(18-c-6)]$_2$; *Right* - Molecular structure of **[2^{Me}−*2H*$^+$]**[K(18-c-6)]$_2$ with thermal ellipsoids plotted at 50 % probability.

Identification code	MnHEA_depro_222crypt_0m
Empirical formula	$C_{52}H_{86}Mn_2N_6Na_2O_{20}S_2$
Formula weight	1335.24
Temperature/K	110.0
Crystal system	triclinic
Space group	P-1
a/Å	10.5714(9)
b/Å	11.7258(10)
c/Å	13.7425(12)
α/°	102.065(5)
β/°	106.840(4)
γ/°	99.105(5)
Volume/Å^3	1550.3(2)
Z	1
ρ_{calc}g/cm^3	1.430
μ/mm^{-1}	4.726
F(000)	704.0
Crystal size/mm^3	0.2 × 0.2 × 0.1
Radiation	CuKα (λ = 1.54178)
2Θ range for data collection/°	6.978 to 137.312
Index ranges	$-12 \leq h \leq 12$, $-13 \leq k \leq 14$, $-16 \leq l \leq 16$
Reflections collected	37434
Independent reflections	5679 [R_{int} = 0.0759, R_{sigma} = 0.0436]
Data/restraints/parameters	5679/0/387
Goodness-of-fit on F^2	1.027
Final R indexes [I>=2σ (I)]	R_1 = 0.0404, wR_2 = 0.0958
Final R indexes [all data]	R_1 = 0.0561, wR_2 = 0.1063
Largest diff. peak/hole / e Å^{-3}	0.46/-0.37

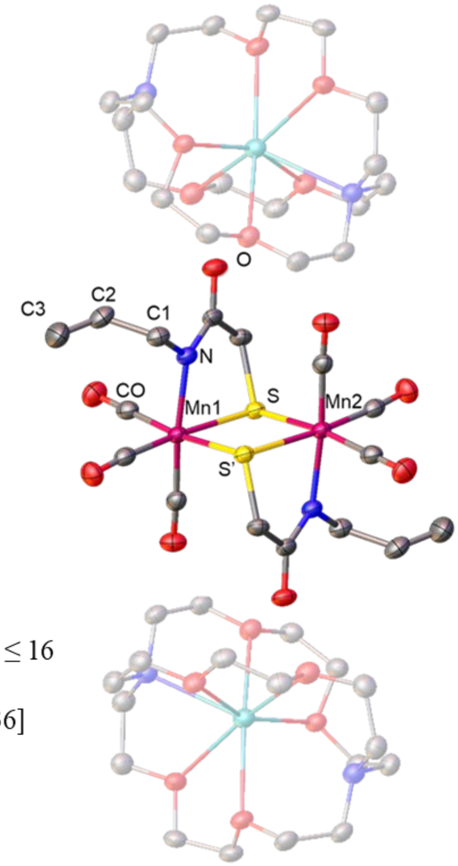

Figure S27. *Left* - Crystal data and structure refinement parameters of **[2^{allyl}–*2H*$^+$]**[K(222-crypt)]$_2$; *Right* - Molecular structure of **[2^{allyl}–*2H*$^+$]**[K(222-crypt)]$_2$ with thermal ellipsoids plotted at 50 % probability.

Identification code	single_depro_0m
Empirical formula	$C_{32}H_{43.77}KMn_2N_2O_{16}S_2$
Formula weight	925.56
Temperature/K	100.0
Crystal system	triclinic
Space group	P-1
a/Å	14.926(2)
b/Å	16.230(2)
c/Å	17.478(3)
α/°	88.644(4)
β/°	89.660(4)
γ/°	82.553(4)
Volume/Å³	4196.9(11)
Z	4
ρ_{calc}g/cm³	1.465
μ/mm⁻¹	0.868
F(000)	1915.0
Crystal size/mm³	0.2 × 0.2 × 0.1
Radiation	MoKα (λ = 0.71073)
2Θ range for data collection/°	3.48 to 51.008
Index ranges	-17 ≤ h ≤ 18, -19 ≤ k ≤ 19, -21 ≤ l ≤ 21
Reflections collected	85232
Independent reflections	15506 [R_{int} = 0.1485, R_{sigma} = 0.1145]
Data/restraints/parameters	15506/375/1086
Goodness-of-fit on F²	1.032
Final R indexes [I>=2σ (I)]	R_1 = 0.0868, wR_2 = 0.2269
Final R indexes [all data]	R_1 = 0.1567, wR_2 = 0.2746
Largest diff. peak/hole / e Å⁻³	1.37/-0.75

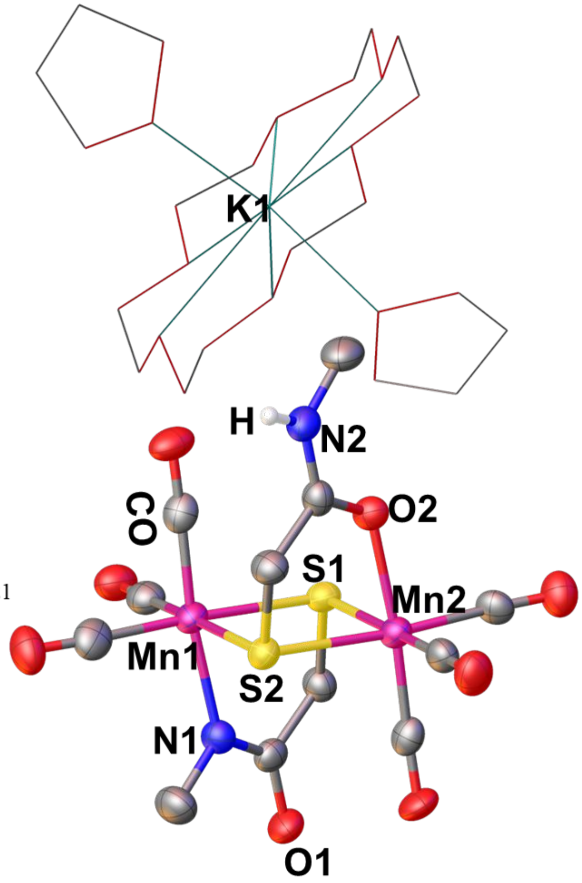

Figure S28. *Left* - Crystal data and structure refinement parameters of **[2^{Me}–*H*$^+$]**[K(18-c-6)]; *Right* - Molecular structure of **[2^{Me}–*H*$^+$]**[K(18-c-6)] with thermal ellipsoids plotted at 50 % probability; [K(18-c-6)]$^+$ is drawn as wireframe for clarity.

Identification code	MnHEPh_depro_15c5_0ma
Empirical formula	$C_{42}H_{50}Mn_2N_2Na_2O_{18}S_2$
Formula weight	1090.82
Temperature/K	110.0
Crystal system	triclinic
Space group	P-1
a/Å	9.560(2)
b/Å	10.7504(19)
c/Å	12.986(2)
α/°	88.027(7)
β/°	71.569(5)
γ/°	75.152(7)
Volume/Å^3	1222.3(4)
Z	1
ρ_{calc}g/cm^3	1.482
μ/mm^{-1}	5.812
F(000)	564.0
Crystal size/mm^3	0.2 × 0.1 × 0.1
Radiation	CuKα (λ = 1.54178)
2Θ range for data collection/°	7.184 to 153.85
Index ranges	$-11 \leq h \leq 11$, $-13 \leq k \leq 13$, $0 \leq l \leq 16$
Reflections collected	4862
Independent reflections	4862 [R_{int} = ?, R_{sigma} = 0.0397]
Data/restraints/parameters	4862/0/308
Goodness-of-fit on F^2	1.152
Final R indexes [I>=2σ (I)]	R_1 = 0.0790, wR_2 = 0.2409
Final R indexes [all data]	R_1 = 0.0835, wR_2 = 0.2450
Largest diff. peak/hole / e Å^{-3}	1.20/-0.96

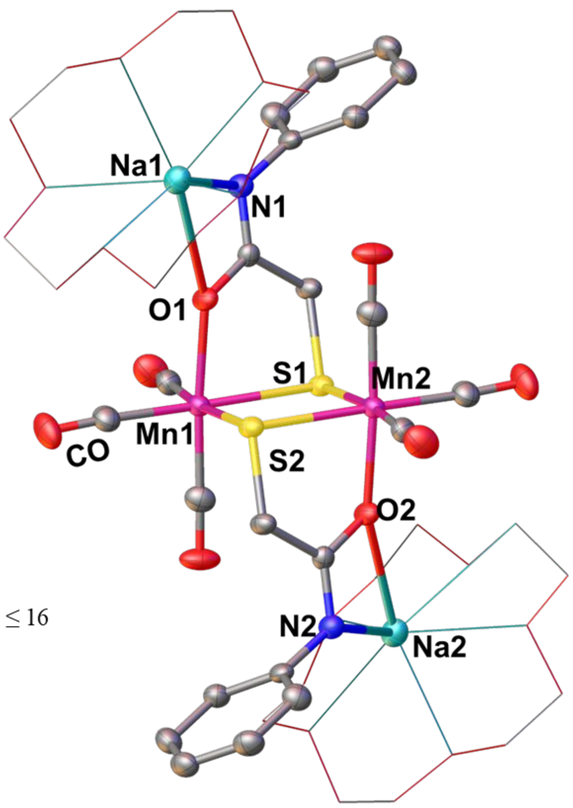

Figure S29. *Left* - Crystal data and structure refinement parameters of **[2^{Ph}−*2H*$^+$]**[Na(15-c-5)]$_2$; *Right* - Molecular structure of **[2^{Ph}−*2H*$^+$]**[Na(15-c-5)]$_2$ with thermal ellipsoids plotted at 50 % probability; 15-c-5 moieties were shown as wireframes for clarity.

Identification code	051121_Mn_Me2HE_0ma
Empirical formula	$C_{14}H_{16}Mn_2N_2O_8S_2$
Formula weight	514.29
Temperature/K	110.0
Crystal system	triclinic
Space group	P-1
a/Å	7.0234(3)
b/Å	8.6338(5)
c/Å	8.8619(4)
α/°	78.658(2)
β/°	72.217(2)
γ/°	78.614(2)
Volume/Å^3	496.25(4)
Z	1
ρ_{calc}g/cm^3	1.721
μ/mm^{-1}	12.733
F(000)	260.0
Crystal size/mm^3	0.1 × 0.05 × 0.05
Radiation	CuKα (λ = 1.54178)
2Θ range for data collection/°	10.566 to 144.274
Index ranges	$-8 \le h \le 8$, $-10 \le k \le 10$, $-10 \le l \le 10$
Reflections collected	5068
Independent reflections	1804 [R_{int} = 0.0357, R_{sigma} = 0.0402]
Data/restraints/parameters	1804/0/129
Goodness-of-fit on F^2	1.109
Final R indexes [I>=2σ (I)]	R_1 = 0.0382, wR_2 = 0.0864
Final R indexes [all data]	R_1 = 0.0419, wR_2 = 0.0898
Largest diff. peak/hole / e Å^{-3}	0.43/-0.43

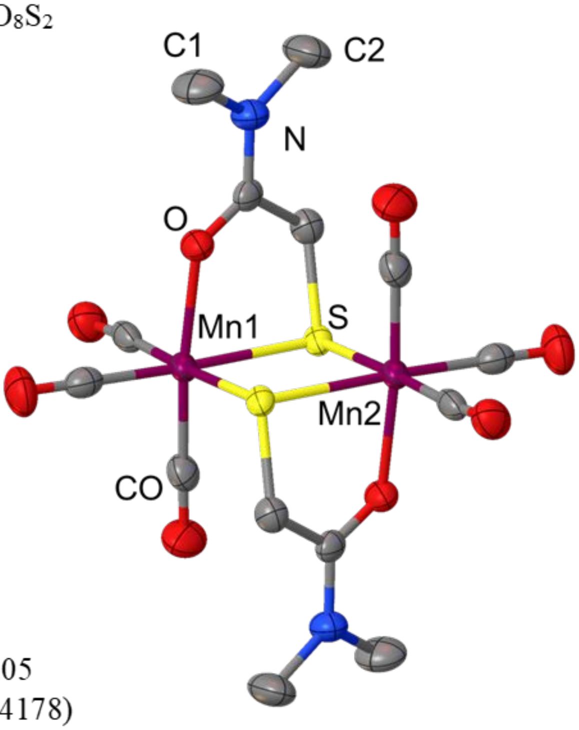

Figure S30. *Left* - Crystal data and structure refinement parameters of **3^{Me}**; *Right* - Molecular structure of **3^{Me}** with thermal ellipsoids plotted at 50 % probability.

Identification code	white_crystal_from_MnHEB_red_0
Empirical formula	$C_{18}H_{24}Mn_2N_2O_8S_2$
Formula weight	570.39
Temperature/K	110.0
Crystal system	triclinic
Space group	P-1
a/Å	7.4994(14)
b/Å	8.9568(17)
c/Å	9.3344(17)
α/°	92.669(7)
β/°	105.713(7)
γ/°	106.055(7)
Volume/Å^3	575.00(19)
Z	1
ρ_{calc}g/cm^3	1.647
μ/mm^{-1}	11.052
F(000)	292.0
Crystal size/mm^3	0.1 × 0.1 × 0.1
Radiation	CuKα (λ = 1.54178)
2Θ range for data collection/°	9.93 to 145.032
Index ranges	$-9 \le h \le 9$, $-10 \le k \le 11$, $-11 \le l \le 11$
Reflections collected	16246
Independent reflections	2247 [R_{int} = 0.0701, R_{sigma} = 0.0371]
Data/restraints/parameters	2247/0/147
Goodness-of-fit on F^2	1.183
Final R indexes [I>=2σ (I)]	R_1 = 0.0435, wR_2 = 0.1316
Final R indexes [all data]	R_1 = 0.0472, wR_2 = 0.1375
Largest diff. peak/hole / e Å^{-3}	0.75/-0.82

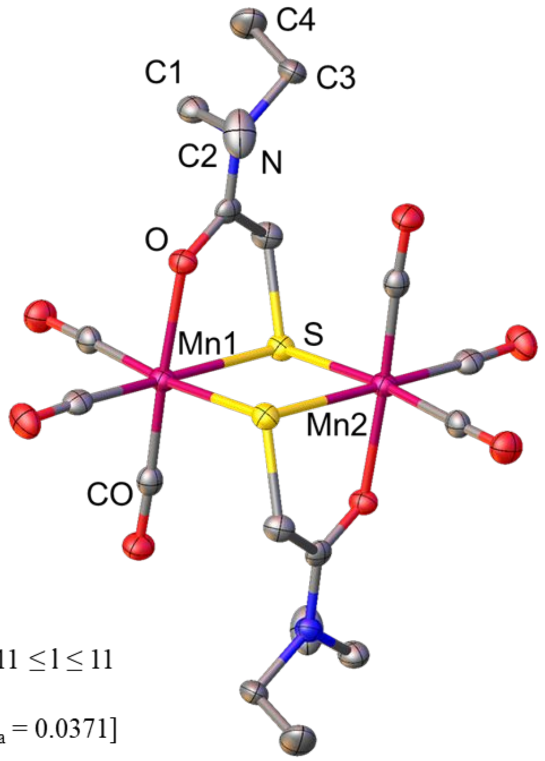

Figure S31. *Left* - Crystal data and structure refinement parameters of $\mathbf{3^{Et}}$; *Right* - Molecular structure of $\mathbf{3^{Et}}$ with thermal ellipsoids plotted at 50 % probability.

Identification code	Mn_Et2HE_red_0m
Empirical formula	$C_{54}H_{96}K_2Mn_2N_6O_{20}S_2$
Formula weight	1401.56
Temperature/K	110.00
Crystal system	triclinic
Space group	P-1
a/Å	11.3597(15)
b/Å	11.5496(15)
c/Å	26.014(3)
α/°	78.830(5)
β/°	85.096(5)
γ/°	89.883(5)
Volume/Å³	3335.7(8)
Z	2
ρ_{calc}g/cm³	1.395
μ/mm^{-1}	0.639
F(000)	1484.0
Crystal size/mm³	0.2 × 0.2 × 0.1
Radiation	MoKα (λ = 0.71073)
2Θ range for data collection/°	3.81 to 60.702
Index ranges	-16 ≤ h ≤ 16, -16 ≤ k ≤ 16, -36 ≤ l ≤ 36
Reflections collected	104901
Independent reflections	19806 [R_{int} = 0.1056, R_{sigma} = 0.0844]
Data/restraints/parameters	19806/0/779
Goodness-of-fit on F²	1.053
Final R indexes [I>=2σ (I)]	R_1 = 0.0542, wR_2 = 0.0978
Final R indexes [all data]	R_1 = 0.1108, wR_2 = 0.1198
Largest diff. peak/hole / e Å^{-3}	0.55/-0.63

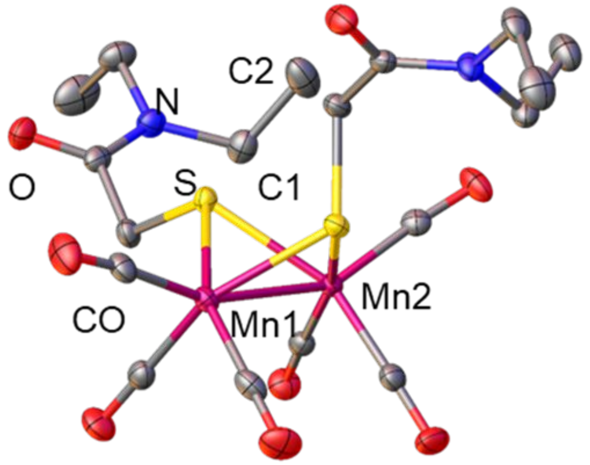

Figure S32. *Left* - Crystal data and structure refinement parameters of **[3^{Et}]**[K(222-crypt)]$_2$; *Right* - Molecular structure of **[3^{Et}]**[K(222-crypt)]$_2$ with thermal ellipsoids plotted at 50 % probability; [K(222-crypt)]$^+$ moieties were omitted for clarity.

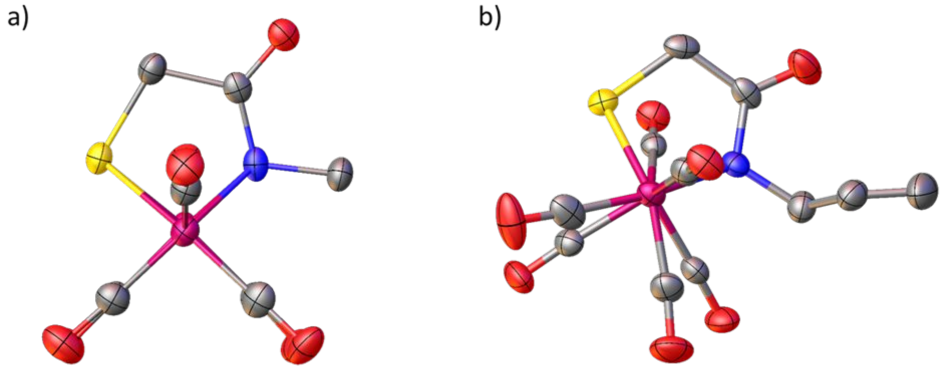

Figure S33. View down Mn—Mn axis of a) dianionic **2^{Me}** displaying the CO ligands are eclipsed and b) dianionic **2^{allyl}** displaying the 20° torsion angle.

Identification code	MnMeHEB
Empirical formula	$C_{50}H_{70}Mn_2N_2Na_2O_{20}S_2$
Formula weight	1239.06
Temperature/K	110.0
Crystal system	monoclinic
Space group	$P2_1$
a/Å	10.9368(19)
b/Å	13.909(2)
c/Å	19.232(3)
α/°	90
β/°	98.187(5)
γ/°	90
Volume/Å^3	2895.8(8)
Z	2
ρ_{calc}g/cm^3	1.421
μ/mm^{-1}	0.597
F(000)	1296.0
Crystal size/mm^3	0.2 × 0.1 × 0.1
Radiation	MoKα (λ = 0.71073)
2Θ range for data collection/°	3.626 to 56.738
Index ranges	-14 ≤ h ≤ 14, -18 ≤ k ≤ 18, -25 ≤ l ≤ 25
Reflections collected	90830
Independent reflections	14492 [R_{int} = 0.1417, R_{sigma} = 0.1083]
Data/restraints/parameters	14492/883/1032
Goodness-of-fit on F^2	1.062
Final R indexes [I>=2σ (I)]	R_1 = 0.0558, wR_2 = 0.1264
Final R indexes [all data]	R_1 = 0.1345, wR_2 = 0.1725
Largest diff. peak/hole / e Å^{-3}	0.64/-0.55
Flack parameter	-0.027(16)

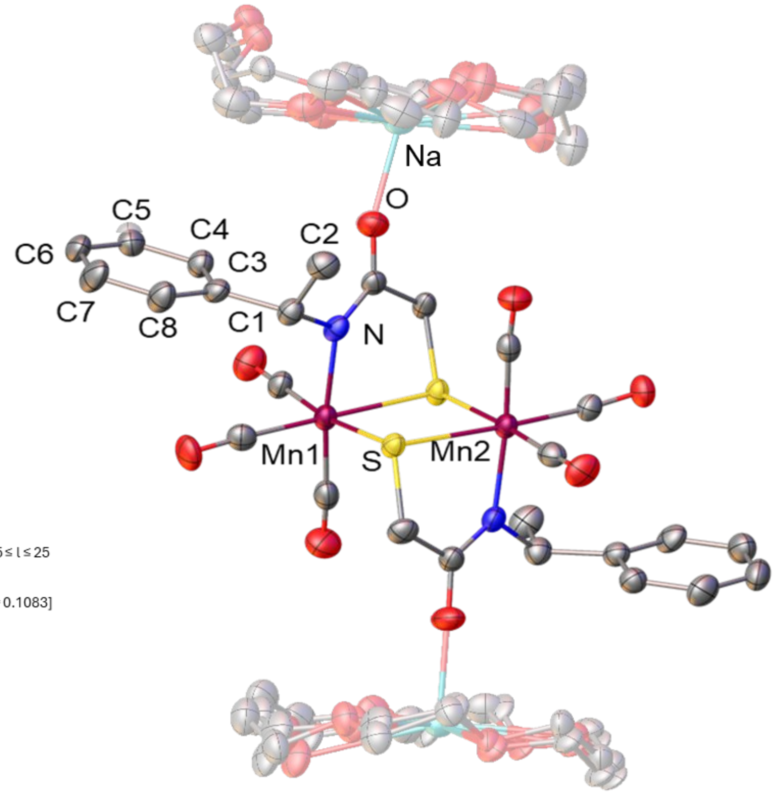

Figure S34. *Left* - Crystal data and structure refinement parameters of **[MnMeHEB-*2H*$^+$]**[Na(18-c-6)]$_2$; *Right* - Molecular structure of **[MnMeHEB-*2H*$^+$]**[Na(18-c-6)]$_2$ with thermal ellipsoids plotted at 50 % probability. Note the disorder of the 18-crown-6 rings.

IV. Computation

DFT calculations were performed using the TPSSTPSS[6] functional, and the triple-ζ basis set 6-311+G[7-10] for Mn and 6-311++G(d,p) for non-metals in Gaussian16 Revision C.01.[11] Transition state structures were located using the Synchronous Transit-Guided Quasi-Newton method from the structures provided from reactants and products with the initial guess for the transition states (***qst3***).[12] Free energies of calculated minima and transition states were corrected for solvation via the SMD model.[13]

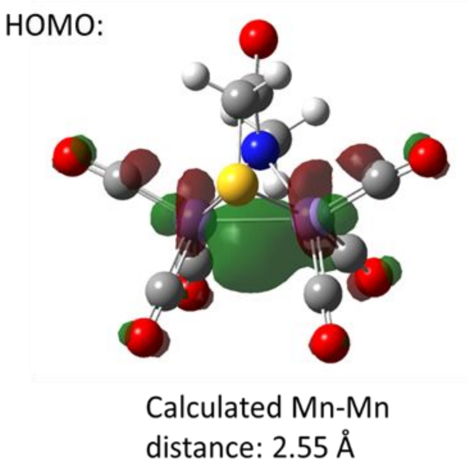

Figure S35. Calculated HOMO of **2^{Me}-Red** displaying Mn—Mn interaction through d_{z^2} orbitals.

Computational analysis on the role of amide binding mode switching and the formation of 2^{Me}-Red .

Visually, the isomerization from O- to N-bound must require rotation around the amide-NCO moiety through an O-atom dissociation. However, DFT computations find that the dissociation of the O atom while keeping the dimeric nature is unlikely, as the potential energy surface (PES) scan along the Mn—O bond suggests a high kinetic barrier, Figure S35.

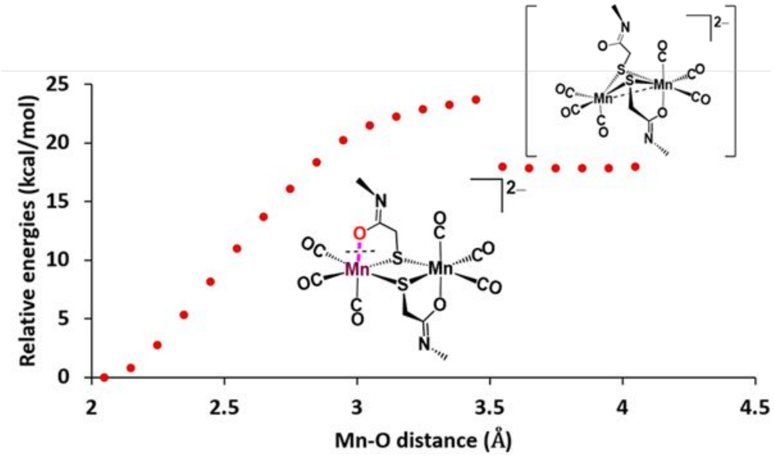

Figure S36. Potential energy surface scan along the Mn—O bond showing a high barrier and unfavorable intermediate.

The resulting intermediate of the O atom dissociation, which is 17 kcal/mol higher in free energy, is also unfavorable. A more viable pathway, suggested by computation, involves cleavage at the diamond [2Mn2S] core, as summarized in Figure S37, similar to what we previously proposed for the *syn/anti* isomerization of $\mathbf{2^{Me}}$.[1] The 16e^- intermediate resulting from the diamond core cleavage is only 5.4 kcal/mol higher in energy compared to the dimeric species, which appears to be a more favorable intermediate compared to the one resulting from O atom dissociation. Additionally, a reasonable transition state for the O- to N-bound isomerization involves an η^3-interaction of the amide via the delocalized π electrons across all three N,C and O atoms, Figure S36 – TS2. This effectively creates a pseudo-octahedral geometry around the Mn(I) center, affirming the need for an open site. It suggests that the O- to N-bound isomerization within these coordinatively saturated Mn(I) centers is most likely to occur via monomeric intermediates. Accordingly, 1-e^- reduction of the N-bound mononuclear intermediate was also found computationally to be more feasible than $\mathbf{[2^{Me}\text{–}\mathit{2H^+}]^{2-}}$ with calculated reduction potential of −1.8 V vs. Fc^+/Fc. This reduction results in a 17e^- Mn(0) species having a trigonal bipyramidal geometry, Figure S36 – B. Combining these computational findings and the experimental analyses, we propose that upon reacting with KC_8, the amide protons of $\mathbf{2^{Me}}$ are reduced first, forming an O-bound deprotonated species. This O-bound deprotonated species then undergoes O- to N-bound isomerization via monomeric intermediates. The N-bound monomeric intermediate then either gets reduced in the presence of excess KC_8 to eventually form $\mathbf{2^{Me}}$**-Red** or dimerizes to form $\mathbf{[2^{Me}\text{–}\mathit{2H^+}]^{2-}}$. As experimental evidence of how the 17-e^- Mn(0) intermediates couple together to generate $\mathbf{2^{Me}}$**-Red** is lacking, further speculation is unwarranted.

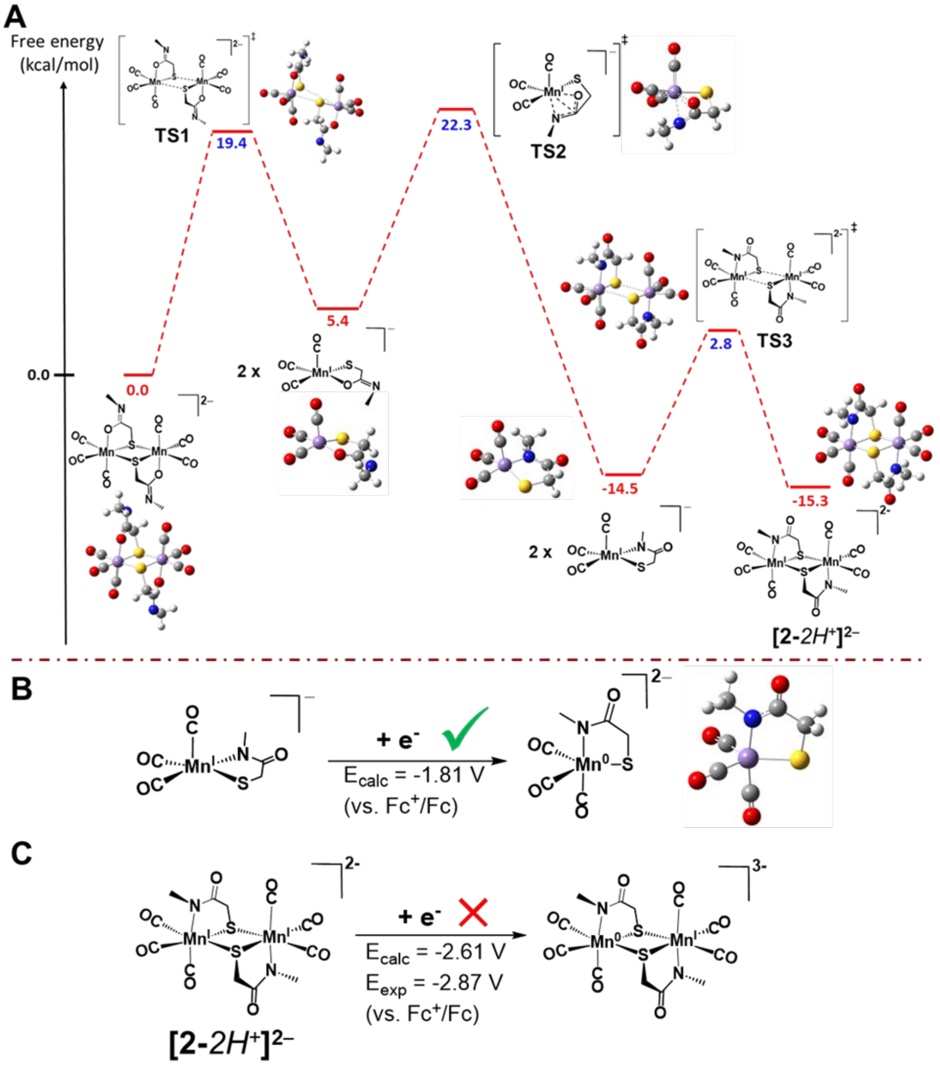

Figure S37. A – A possible pathway of the O- to N-bound isomerization involving monomeric intermediates as suggested by DFT calculation; **B** – Calculated 1e^- reduction potential of the monomeric $Mn(CO)_3$(S—N) anion; **C** – Calculated 1e^- reduction potential of $\mathbf{[2^{Me}–}\boldsymbol{2H^+}\mathbf{]^{2–}}$ in comparision with the experimental value.

References

(1) Le, T. H.; Nguyen, H.; Arnold, H. A.; Darensbourg, D. J.; Darensbourg, M. Y., Chirality-Guided Isomerization of Mn2S2 Diamond Core Complexes: A Mechanistic Study. *Inorg. Chem.* **2022,** *61* (41), 16405-16413.
(2) Le, T.; Nguyen, H.; Perez, L. M.; Darensbourg, D. J.; Darensbourg, M. Y., Metal-Templated, Tight Loop Conformation of a Cys-X-Cys Biomimetic Assembles a Dimanganese Complex. *Angew. Chem. Int. Ed.* **2020,** *59* (9), 3645-3649.

(3) Sheldrick, G. M., SHELXT - Integrated space-group and crystal-structure determination. *Acta Crystallographica a-Foundation and Advances* **2015,** *71*, 3-8.
(4) Sheldrick, G. M., Crystal structure refinement with SHELXL. *Acta Crystallographica Section C-Structural Chemistry* **2015,** *71*, 3-8.
(5) Dolomanov, O. V.; Bourhis, L. J.; Gildea, R. J.; Howard, J. A. K.; Puschmann, H., OLEX2: a complete structure solution, refinement and analysis program. *J. Appl. Crystallogr.* **2009,** *42*, 339-341.
(6) Tao, J. M.; Perdew, J. P.; Staroverov, V. N.; Scuseria, G. E., Climbing the density functional ladder: Nonempirical meta-generalized gradient approximation designed for molecules and solids. *Phys. Rev. Lett.* **2003,** *91* (14).
(7) Krishnan, R.; Binkley, J. S.; Seeger, R.; Pople, J. A., Self-consistent molecular-orbital methods .20. Basis set for correlated wave-functions. *J. Chem. Phys.* **1980,** *72* (1), 650-654.
(8) McLean, A. D.; Chandler, G. S., Contracted gaussian-basis sets for molecular calculations .1. 2nd row atoms, z=11-18. *J. Chem. Phys.* **1980,** *72* (10), 5639-5648.
(9) Wachters, A. J., Gaussian basis set for molecular wavefunctions containing third-row atoms. *J. Chem. Phys.* **1970,** *52* (3), 1033-&.
(10) Raghavachari, K.; Trucks, G. W., Highly correlated systems - excitation-energies of 1st row transition-metals Sc-Cu. *J. Chem. Phys.* **1989,** *91* (2), 1062-1065.
(11) Frisch, M. J.; Trucks, G. W.; Schlegel, H. B.; Scuseria, G. E.; Robb, M. A.; Cheeseman, J. R.; Scalmani, G.; Barone, V.; Petersson, G. A.; Nakatsuji, H.; Li, X.; Caricato, M.; Marenich, A. V.; Bloino, J.; Janesko, B. G.; Gomperts, R.; Mennucci, B.; Hratchian, H. P.; Ortiz, J. V.; Izmaylov, A. F.; Sonnenberg, J. L.; Williams; Ding, F.; Lipparini, F.; Egidi, F.; Goings, J.; Peng, B.; Petrone, A.; Henderson, T.; Ranasinghe, D.; Zakrzewski, V. G.; Gao, J.; Rega, N.; Zheng, G.; Liang, W.; Hada, M.; Ehara, M.; Toyota, K.; Fukuda, R.; Hasegawa, J.; Ishida, M.; Nakajima, T.; Honda, Y.; Kitao, O.; Nakai, H.; Vreven, T.; Throssell, K.; Montgomery Jr., J. A.; Peralta, J. E.; Ogliaro, F.; Bearpark, M. J.; Heyd, J. J.; Brothers, E. N.; Kudin, K. N.; Staroverov, V. N.; Keith, T. A.; Kobayashi, R.; Normand, J.; Raghavachari, K.; Rendell, A. P.; Burant, J. C.; Iyengar, S. S.; Tomasi, J.; Cossi, M.; Millam, J. M.; Klene, M.; Adamo, C.; Cammi, R.; Ochterski, J. W.; Martin, R. L.; Morokuma, K.; Farkas, O.; Foresman, J. B.; Fox, D. J. *Gaussian 16 Rev. C.01*, Wallingford, CT, 2016.
(12) Peng, C.; Bernhard Schlegel, H., Combining Synchronous Transit and Quasi-Newton Methods to Find Transition States. *Isr. J. Chem.* **1993,** *33* (4), 449-454.
(13) Marenich, A. V.; Cramer, C. J.; Truhlar, D. G., Universal Solvation Model Based on Solute Electron Density and on a Continuum Model of the Solvent Defined by the Bulk Dielectric Constant and Atomic Surface Tensions. *The Journal of Physical Chemistry B* **2009,** *113* (18), 6378-6396.

Coordinates:

- **[2^Me^−2*H*^+^]**$^{2-}$ O-bound optimization:

-2 1

Mn	6.45590000	6.37710000	1.29280000
S	4.66760000	5.06490000	0.49920000
O	6.96310000	4.64290000	2.26590000
O	5.65610000	8.75060000	-0.25760000
N	6.23660000	2.94050000	3.52320000
O	8.80970000	7.94350000	2.18100000
O	4.73430000	7.42370000	3.45390000
C	6.00980000	3.82960000	2.62910000
C	5.97600000	7.80730000	0.33350000
C	7.91810000	7.29420000	1.85190000
C	4.64470000	3.98080000	1.94870000
H	3.99850000	4.34110000	2.60640000
H	4.32490000	3.08550000	1.67250000
C	5.41900000	6.99770000	2.64220000
Mn	5.85910000	4.01430000	-1.29280000
S	7.64740000	5.32650000	-0.49920000
O	5.35200000	5.74850000	-2.26590000
O	6.65900000	1.64080000	0.25760000
N	6.07840000	7.45090000	-3.52320000
O	3.50540000	2.44790000	-2.18100000
O	7.58080000	2.96780000	-3.45390000
C	6.30530000	6.56180000	-2.62910000
C	6.33900000	2.58410000	-0.33350000
C	4.39700000	3.09720000	-1.85190000
C	7.67030000	6.41060000	-1.94870000
H	8.31660000	6.05030000	-2.60640000
H	7.99010000	7.30590000	-1.67250000
C	6.89600000	3.39380000	-2.64220000
C	4.73057381	7.50345003	-4.10758263
H	4.01161448	7.66588490	-3.33194587
H	4.67945443	8.30477384	-4.81480746
H	4.51958218	6.57794200	-4.60136134
C	7.58611725	2.88368641	4.10326500
H	7.80197037	3.80886236	4.59556257
H	7.63669822	2.08278715	4.81100926
H	8.30198444	2.71805562	3.32544715

- **[2^{Me}–2*H*$^+$]$^{2-}$** O-bound cleavage TS: ***qst3***

dimer O bound

-2 1

Mn	-1.40826700	-1.13596100	-0.02112700
S	-0.28033000	0.27757800	1.54765800
O	-2.35160100	0.61875400	-0.49875600
O	-0.05722000	-3.61420500	0.86454000
N	-3.24415100	2.62706500	0.24494800
O	-2.72351900	-2.62935800	-2.22534300
O	-3.55487200	-1.94278700	1.86011400
C	-2.47487800	1.57914300	0.38225400
C	-0.52810000	-2.61355800	0.47868100
C	-2.20986000	-2.02681200	-1.36486700
C	-1.69222500	1.45562400	1.68755700
H	-2.35213000	1.05198000	2.46756600
H	-1.33071300	2.43267700	2.02206200
C	-2.71323700	-1.58583600	1.12934600
Mn	1.40827000	1.13596300	0.02117200
S	0.28020200	-0.27743800	-1.54763100
O	2.35146800	-0.61885700	0.49869900
O	0.05745400	3.61439700	-0.86430200
N	3.24405900	-2.62705700	-0.24526500
O	2.72375500	2.62901900	2.22548200
O	3.55496300	1.94279600	-1.85996200
C	2.47477000	-1.57913000	-0.38243600
C	0.52821900	2.61367300	-0.47849700
C	2.20999100	2.02662300	1.36496600
C	1.69209100	-1.45546800	-1.68771300
H	2.35197800	-1.05173700	-2.46769400
H	1.33056800	-2.43248400	-2.02231600
C	2.71328000	1.58582200	-1.12925900
C	4.02519300	-2.67111400	0.99666600
H	3.38207400	-2.75949300	1.88797200
H	4.68725600	-3.54531500	0.96961700
H	4.64504300	-1.77035700	1.13816700
C	-4.02528200	2.67097600	-0.99699000
H	-4.64515800	1.77021800	-1.13837200
H	-4.68731900	3.54520100	-0.97006100
H	-3.38215800	2.75921700	-1.88830500

cleaved

-2 1

Mn	-4.08591075	-2.96599611	2.89606065
S	-2.95797375	-1.55245711	4.46484565
O	-5.02924475	-1.21128111	2.41843165
O	-2.73486375	-5.44424011	3.78172765
N	-5.92179475	0.79702989	3.16213565
O	-5.40116275	-4.45939311	0.69184465

O	-6.23251575	-3.77282211	4.77730165
C	-5.15252175	-0.25089211	3.29944165
C	-3.20574375	-4.44359311	3.39586865
C	-4.88750375	-3.85684711	1.55232065
C	-4.36986875	-0.37441111	4.60474465
H	-5.02977375	-0.77805511	5.38475365
H	-4.00835675	0.60264189	4.93924965
C	-5.39088075	-3.41587111	4.04653365
Mn	1.40827000	1.13596300	0.02117200
S	0.28020200	-0.27743800	-1.54763100
O	2.35146800	-0.61885700	0.49869900
O	0.05745400	3.61439700	-0.86430200
N	3.24405900	-2.62705700	-0.24526500
O	2.72375500	2.62901900	2.22548200
O	3.55496300	1.94279600	-1.85996200
C	2.47477000	-1.57913000	-0.38243600
C	0.52821900	2.61367300	-0.47849700
C	2.20999100	2.02662300	1.36496600
C	1.69209100	-1.45546800	-1.68771300
H	2.35197800	-1.05173700	-2.46769400
H	1.33056800	-2.43248400	-2.02231600
C	2.71328000	1.58582200	-1.12925900
C	4.02519300	-2.67111400	0.99666600
H	3.38207400	-2.75949300	1.88797200
H	4.68725600	-3.54531500	0.96961700
H	4.64504300	-1.77035700	1.13816700
C	-6.70292575	0.84094089	1.92019765
H	-7.32280175	-0.05981711	1.77881565
H	-7.36496275	1.71516589	1.94712665
H	-6.05980175	0.92918189	1.02888265

TS guess

-2 1

Mn	-2.14195623	-1.76744860	1.60856515
S	-1.01401923	-0.35390960	3.17735015
O	-3.08529023	-0.01273360	1.13093615
O	-0.79090923	-4.24569260	2.49423215
N	-3.97784023	1.99557740	1.87464015
O	-3.45720823	-3.26084560	-0.59565085
O	-4.28856123	-2.57427460	3.48980615
C	-3.20856723	0.94765540	2.01194615
C	-1.26178923	-3.24504560	2.10837315
C	-2.94354923	-2.65829960	0.26482515
C	-2.42591423	0.82413640	3.31724915
H	-3.08581923	0.42049240	4.09725815
H	-2.06440223	1.80118940	3.65175415
C	-3.44692623	-2.21732360	2.75903815
Mn	1.40827000	1.13596300	0.02117200
S	0.28020200	-0.27743800	-1.54763100
O	2.35146800	-0.61885700	0.49869900

O	0.05745400	3.61439700	-0.86430200
N	3.24405900	-2.62705700	-0.24526500
O	2.72375500	2.62901900	2.22548200
O	3.55496300	1.94279600	-1.85996200
C	2.47477000	-1.57913000	-0.38243600
C	0.52821900	2.61367300	-0.47849700
C	2.20999100	2.02662300	1.36496600
C	1.69209100	-1.45546800	-1.68771300
H	2.35197800	-1.05173700	-2.46769400
H	1.33056800	-2.43248400	-2.02231600
C	2.71328000	1.58582200	-1.12925900
C	4.02519300	-2.67111400	0.99666600
H	3.38207400	-2.75949300	1.88797200
H	4.68725600	-3.54531500	0.96961700
H	4.64504300	-1.77035700	1.13816700
C	-4.75897123	2.03948840	0.63270215
H	-5.37884723	1.13873040	0.49132015
H	-5.42100823	2.91371340	0.65963115
H	-4.11584723	2.12772940	-0.25861285

- Monomeric O-bound optimization:

-1 1

S	-0.03825600	2.04859700	0.32635200
O	1.38401700	-2.92683900	0.72386000
O	1.59163800	-0.31571300	-2.62711600
O	3.41325600	0.79951400	0.96925300
C	-2.19160900	0.31643300	0.00780000
C	1.24762300	-0.24736400	-1.50993400
C	2.34556400	0.44752000	0.64909800
C	-1.80574500	1.77590200	-0.13398300
H	-1.92893400	2.08797900	-1.17859400
H	-2.44652700	2.41072800	0.48476300
C	1.10097600	-1.81706200	0.50179600
Mn	0.70224800	-0.08145600	0.15282300
N	-3.45218000	0.00472000	0.08133100
O	-1.20739400	-0.56740500	0.01943600
C	-4.38566773	1.13583503	0.16624770
H	-4.33234416	1.78609326	-0.72113720
H	-5.40692310	0.74541786	0.24484490
H	-4.18966356	1.77124732	1.04440773

- Monomeric O-bound to N-bound TS: ***qst3***

O bound

-1 1

S	-0.41077858	-1.84845193	-0.20615117
O	2.19505685	2.53697088	-1.03144651
O	1.59720404	0.46272363	2.63493211
O	3.25330021	-1.55883228	-0.63083085

C	-2.10070790	0.38299471	-0.10936162
C	1.29977428	0.31884199	1.51141023
C	2.29273340	-0.91776107	-0.44783930
C	-2.10473516	-1.13725567	-0.07888212
H	-2.56899412	-1.49400247	0.84947480
H	-2.70987057	-1.52473038	-0.90867583
C	1.63800603	1.57199400	-0.68522080
Mn	0.80826201	0.04966105	-0.15571046
N	-3.18228715	1.10412152	-0.05322152
O	-0.92359278	0.97830761	-0.20840772
C	-4.45957997	0.38957568	0.03294306
H	-4.52528821	-0.25860825	0.92359537
H	-5.27075375	1.12369250	0.09597419
H	-4.65186695	-0.24519397	-0.84891060

N bound

-1 1

S	0.49843200	-2.06148700	-0.12621700
O	-1.80180600	2.22845400	-1.50470200
O	-3.11296400	-1.58590400	-0.42746800
O	-1.44145500	0.94026900	2.52368200
C	2.41177500	-0.00241000	-0.06707300
C	-2.11130100	-0.99635200	-0.30166300
C	-1.11269800	0.55463800	1.46719300
C	2.24529800	-1.51110300	-0.01819200
H	2.81181600	-1.96496500	-0.83895000
H	2.67597900	-1.88053400	0.92074500
C	-1.29651000	1.34371900	-0.93063400
Mn	-0.57408200	-0.07534800	-0.09218100
N	1.26509800	0.72174100	-0.05123900
O	3.57006500	0.48767500	-0.10254300
C	1.48196900	2.18105000	-0.02455600
H	1.67622300	2.57271700	-1.03469100
H	0.58947400	2.67241800	0.37147200
H	2.34604100	2.43445600	0.60190800

TS guess

-1 1

S	0.216060	-2.091720	0.739592
O	-1.663221	2.638782	-0.810627
O	-2.819753	-1.342424	-1.240013
O	-2.276453	0.307235	2.653455
C	1.601448	0.045389	0.147922
C	-1.962765	-0.794359	-0.667937
C	-1.642771	0.206626	1.676819
C	1.919090	-1.377769	0.612584
H	2.492280	-1.913442	-0.146739
H	2.431354	-1.400662	1.577185
C	-1.210772	1.644954	-0.396929

Mn	-0.652541	0.047242	0.228317
N	1.160068	0.877715	1.134980
O	1.608689	0.319469	-1.095822
C	1.308364	2.324646	0.877071
H	0.553015	2.875262	1.447273
H	2.298171	2.638408	1.244931
H	1.233328	2.600493	-0.183572

- Monomeric N-bound optimization:

-1 1

S	0.52836680	-1.61454845	-1.23672879
O	-0.95724209	3.01648799	-2.63006193
O	-2.81325896	-0.71681521	-2.41432156
O	-1.81552331	1.31026569	1.16062533
C	2.51088002	0.20552429	-0.42361495
C	-1.84032283	-0.25631144	-1.95818695
C	-1.24248539	0.99549234	0.18825363
C	2.21739588	-1.27317781	-0.60682433
H	2.94848800	-1.70460028	-1.29961291
H	2.34416311	-1.77749869	0.35917702
C	-0.70041443	2.02977925	-2.05727964
Mn	-0.35037773	0.46487142	-1.24045984
N	1.46518706	1.04662460	-0.62005842
O	3.79118913	0.50814536	0.21102803
C	1.78238545	2.46356684	-0.35740342
H	2.27592329	2.92794713	-1.22474517
H	0.85786497	3.00937615	-0.15202062
H	2.45975739	2.55798227	0.50023586

- **[2−2*H*$^+$]$^{2-}$** N-bound formation TS: ***qst3***

monomeric

-2 1

Mn	-8.64765446	1.13457163	-1.88826180
Mn	1.70040800	-0.61124900	0.07764500
S	-7.08846146	0.01662263	-3.31989280
S	0.14127000	0.50677200	1.50934200
O	-10.89301146	0.96287263	-3.81532780
O	-8.03682046	3.79550663	-3.04967480
O	-10.39322046	2.50288063	0.08348620
N	-8.99548646	-0.80835037	-1.25430080
O	3.94452500	-0.43993500	2.00621500
N	2.04825700	1.33158300	-0.55644600
O	3.44720300	-1.97911600	-1.89332100
C	-9.99754546	1.00677563	-3.06092880
C	-8.21485746	2.74382963	-2.56583580
C	-9.70771246	1.92695063	-0.67266680
C	1.85095200	2.38657200	0.24954700
C	-8.79854446	-1.86313637	-2.06068080

C	1.26736300	-2.22068000	0.75472200
C	3.04970200	-0.48363100	1.25106300
C	1.03454300	2.11945400	1.51532300
H	0.31865200	2.93486000	1.66379400
H	1.71617400	2.11345200	2.37742900
C	-9.79737646	-1.06926537	-0.05525380
H	-9.48214546	-0.39323637	0.74715220
H	-9.67311146	-2.10808637	0.27657820
H	-10.87195946	-0.90143737	-0.24049480
C	-7.98175946	-1.59601537	-3.32617580
H	-8.66311446	-1.59001037	-4.18848280
H	-7.26583646	-2.41142637	-3.47443280
C	2.76112600	-1.40336100	-1.13755100
C	2.84955700	1.59244700	-1.75590600
H	3.92425900	1.42496100	-1.57106300
H	2.72485000	2.63115200	-2.08790900
H	2.53416800	0.91614800	-2.55800900
O	-9.23633946	-3.03572937	-1.87543380
O	1.08923100	-3.27250600	1.23822800
O	2.28824000	3.55927800	0.06381700

dimer

-2 1

Mn	-1.70026800	0.61124900	-0.07762500
Mn	1.70040800	-0.61124900	0.07764500
S	-0.14107500	-0.50670000	-1.50925600
S	0.14127000	0.50677200	1.50934200
O	-3.94562500	0.43955000	-2.00469100
O	-1.08943400	3.27218400	-1.23903800
O	-3.44583400	1.97955800	1.89412300
N	-2.04810000	-1.33167300	0.55633600
O	3.94452500	-0.43993500	2.00621500
N	2.04825700	1.33158300	-0.55644600
O	3.44720300	-1.97911600	-1.89332100
C	-3.05015900	0.48345300	-1.25029200
C	-1.26747100	2.22050700	-0.75519900
C	-2.76032600	1.40362800	1.13797000
C	1.85095200	2.38657200	0.24954700
C	-1.85115800	-2.38645900	-0.25004400
C	1.26736300	-2.22068000	0.75472200
C	3.04970200	-0.48363100	1.25106300
C	1.03454300	2.11945400	1.51532300
H	0.31865200	2.93486000	1.66379400
H	1.71617400	2.11345200	2.37742900
C	-2.84999000	-1.59258800	1.75538300
H	-2.53475900	-0.91655900	2.55778900
H	-2.72572500	-2.63140900	2.08721500
H	-3.92457300	-1.42476000	1.57014200
C	-1.03437300	-2.11933800	-1.51553900
H	-1.71572800	-2.11333300	-2.37784600

H	-0.31845000	-2.93474900	-1.66379600
C	2.76112600	-1.40336100	-1.13755100
C	2.84955700	1.59244700	-1.75590600
H	3.92425900	1.42496100	-1.57106300
H	2.72485000	2.63115200	-2.08790900
H	2.53416800	0.91614800	-2.55800900
O	-2.28895300	-3.55905200	-0.06479700
O	1.08923100	-3.27250600	1.23822800
O	2.28824000	3.55927800	0.06381700

TS guess

-2 1

Mn	-2.70742455	0.84238890	-1.25814027
Mn	1.70040800	-0.61124900	0.07764500
S	-1.14823155	-0.27556010	-2.68977127
S	0.14127000	0.50677200	1.50934200
O	-4.95278155	0.67068990	-3.18520627
O	-2.09659055	3.50332390	-2.41955327
O	-4.45299055	2.21069790	0.71360773
N	-3.05525655	-1.10053310	-0.62417927
O	3.94452500	-0.43993500	2.00621500
N	2.04825700	1.33158300	-0.55644600
O	3.44720300	-1.97911600	-1.89332100
C	-4.05731555	0.71459290	-2.43080727
C	-2.27462755	2.45164690	-1.93571427
C	-3.76748255	1.63476790	-0.04254527
C	1.85095200	2.38657200	0.24954700
C	-2.85831455	-2.15531910	-1.43055927
C	1.26736300	-2.22068000	0.75472200
C	3.04970200	-0.48363100	1.25106300
C	1.03454300	2.11945400	1.51532300
H	0.31865200	2.93486000	1.66379400
H	1.71617400	2.11345200	2.37742900
C	-3.85714655	-1.36144810	0.57486773
H	-3.54191555	-0.68541910	1.37727373
H	-3.73288155	-2.40026910	0.90669973
H	-4.93172955	-1.19362010	0.38962673
C	-2.04152955	-1.88819810	-2.69605427
H	-2.72288455	-1.88219310	-3.55836127
H	-1.32560655	-2.70360910	-2.84431127
C	2.76112600	-1.40336100	-1.13755100
C	2.84955700	1.59244700	-1.75590600
H	3.92425900	1.42496100	-1.57106300
H	2.72485000	2.63115200	-2.08790900
H	2.53416800	0.91614800	-2.55800900
O	-3.29610955	-3.32791210	-1.24531227
O	1.08923100	-3.27250600	1.23822800
O	2.28824000	3.55927800	0.06381700

- **[2^{Me}–2*H*$^{+}$]**$^{2-}$ N-bound optimization:

-2 1			
Mn	6.43850000	6.06470000	10.79950000
Mn	3.25990000	5.76110000	12.31710000
S	5.24260000	4.42480000	12.00680000
S	4.54350000	7.45390000	11.30720000
O	8.66110000	4.22940000	10.15220000
O	8.01230000	6.90620000	13.14330000
O	7.48460000	8.34860000	9.24290000
N	5.27990000	5.31260000	9.26730000
O	0.95800000	7.57180000	12.70670000
N	4.27290000	6.35160000	14.00000000
O	1.86490000	3.42340000	13.47040000
C	7.77870000	4.93760000	10.38280000
C	7.35960000	6.59380000	12.23320000
C	7.08970000	7.43320000	9.83190000
C	4.96490000	7.46990000	14.07070000
C	4.79800000	4.07570000	9.29920000
C	2.52120000	5.37530000	10.73930000
C	1.87620000	6.87780000	12.59330000
C	4.89220000	8.36690000	12.84360000
H	5.75290000	8.84570000	12.74430000
H	4.18400000	9.04430000	12.98250000
C	5.02720000	6.02090000	8.03100000
H	5.18990000	6.97820000	8.16390000
H	4.09600000	5.88240000	7.75940000
H	5.62510000	5.68200000	7.33230000
C	4.98580000	3.31320000	10.59070000
H	5.76680000	2.71160000	10.50300000
H	4.18680000	2.75310000	10.75720000
C	2.42340000	4.35550000	13.06340000
C	4.29150000	5.48720000	15.17480000
H	3.38460000	5.40950000	15.53760000
H	4.88340000	5.87230000	15.85440000
H	4.61930000	4.59890000	14.92180000
O	4.19922910	3.50448634	8.35117103
O	1.98484104	5.19818398	9.77749641
O	5.63600349	7.87222375	15.05625886

- **[2^{Me}–2*H*$^{+}$]$^{2-}$** N-bound reduction:

-3 2			
Mn	-2.26499680	-0.54828374	0.14561677
Mn	1.13567920	-1.77078174	0.30088677
S	-0.70580380	-1.66623274	-1.28601423
S	-0.42345880	-0.65276074	1.73258377
O	-4.51035380	-0.71998274	-1.78144923
O	-1.65416280	2.11265126	-1.01579623
O	-4.01056280	0.82002526	2.11736477
N	-1.14153965	-3.15808845	-3.37626371

O	3.37979620	-1.59946774	2.22945677
N	1.48352820	0.17205026	-0.33320423
O	2.88247420	-3.13864874	-1.67007923
C	-3.61488780	-0.67607974	-1.02705023
C	-1.83219980	1.06097426	-0.53195723
C	-3.32505480	0.24409526	1.36121177
C	1.28622320	1.22703926	0.47278877
C	-1.76601911	-3.81736719	-2.38783803
C	0.70263420	-3.38021274	0.97796377
C	2.48497320	-1.64316374	1.47430477
C	0.46981420	0.95992126	1.73856477
H	-0.24607680	1.77532726	1.88703577
H	1.15144520	0.95391926	2.60067077
C	-1.38175619	-3.66967836	-4.72881304
H	-1.31022522	-2.84566399	-5.44723132
H	-2.37702408	-4.12692945	-4.79999302
H	-0.63892927	-4.43472584	-5.01186711
C	-1.50397958	-3.30509187	-0.97048901
H	-0.83641491	-4.01306496	-0.45962153
H	-2.44721626	-3.27803362	-0.41461727
C	2.19639720	-2.56289374	-0.91430923
C	2.28482820	0.43291426	-1.53266423
H	3.35953020	0.26542826	-1.34782123
H	2.16012120	1.47161926	-1.86466723
H	1.96943920	-0.24338474	-2.33476723
O	-2.52715774	-4.81893420	-2.52380404
O	0.52450220	-4.43203874	1.46146977
O	1.72351120	2.39974526	0.28705877

- Monomeric N-bound reduction:

-2 2

Mn	6.43850000	6.06470000	10.79950000
S	5.24260000	4.42480000	12.00680000
O	8.66110000	4.22940000	10.15220000
O	8.01230000	6.90620000	13.14330000
O	7.48460000	8.34860000	9.24290000
N	5.27990000	5.31260000	9.26730000
C	7.77870000	4.93760000	10.38280000
C	7.35960000	6.59380000	12.23320000
C	7.08970000	7.43320000	9.83190000
C	4.79800000	4.07570000	9.29920000
C	5.02720000	6.02090000	8.03100000
H	5.18990000	6.97820000	8.16390000
H	4.09600000	5.88240000	7.75940000
H	5.62510000	5.68200000	7.33230000
C	4.98580000	3.31320000	10.59070000
H	5.76680000	2.71160000	10.50300000
H	4.18680000	2.75310000	10.75720000
O	4.19922910	3.50448634	8.35117103

- **3** optimization singlet:

-2 1

Mn	6.34980000	3.08480000	11.28120000
Mn	7.52080000	5.00250000	12.42600000
O	5.92530000	0.87640000	9.36680000
O	5.41830000	1.65490000	13.67650000
O	9.69920000	6.92520000	12.89240000
O	5.39640000	7.04510000	12.62400000
O	3.56630000	4.05880000	11.03250000
C	8.87120000	6.14920000	12.64500000
C	6.15260000	1.76670000	10.09230000
C	6.22920000	6.22730000	12.52070000
C	5.79190000	2.21300000	12.70340000
C	7.32750000	4.58170000	14.11790000
C	4.67710000	3.70910000	11.10250000
S	8.54290000	2.96410000	12.02590000
N	7.28460000	4.71930000	10.35800000
C	6.51640000	5.68870000	9.54650000
H	6.46890000	5.37330000	8.61980000
H	6.96190000	6.56110000	9.57230000
H	5.61040000	5.77340000	9.90980000
C	8.50920000	4.47400000	9.78590000
C	9.37190000	3.45780000	10.48940000
H	9.50630000	2.66770000	9.90830000
H	10.25800000	3.84940000	10.69350000
O	8.95495322	5.02803953	8.74767323
O	7.20198480	4.22811062	15.31906237

- **3** optimization triplet:

-2 3

Mn	6.34980000	3.08480000	11.28120000
Mn	7.52080000	5.00250000	12.42600000
O	5.92530000	0.87640000	9.36680000
O	5.41830000	1.65490000	13.67650000
O	9.69920000	6.92520000	12.89240000
O	5.39640000	7.04510000	12.62400000
O	3.56630000	4.05880000	11.03250000
C	8.87120000	6.14920000	12.64500000
C	6.15260000	1.76670000	10.09230000
C	6.22920000	6.22730000	12.52070000
C	5.79190000	2.21300000	12.70340000
C	7.32750000	4.58170000	14.11790000
C	4.67710000	3.70910000	11.10250000
S	8.54290000	2.96410000	12.02590000
N	7.28460000	4.71930000	10.35800000
C	6.51640000	5.68870000	9.54650000
H	6.46890000	5.37330000	8.61980000
H	6.96190000	6.56110000	9.57230000
H	5.61040000	5.77340000	9.90980000

C	8.50920000	4.47400000	9.78590000
C	9.37190000	3.45780000	10.48940000
H	9.50630000	2.66770000	9.90830000
H	10.25800000	3.84940000	10.69350000
O	8.95495322	5.02803953	8.74767323
O	7.20198480	4.22811062	15.31906237

- **[2^{Me}−2*H*$^{+}$]$^{2-}$** Mn—O bond PES scan:

-2 1

Mn	-1.40826700	-1.13596100	-0.02112700
S	-0.28033000	0.27757800	1.54765800
O	-2.35160100	0.61875400	-0.49875600
O	-0.05722000	-3.61420500	0.86454000
N	-3.24415100	2.62706500	0.24494800
O	-2.72351900	-2.62935800	-2.22534300
O	-3.55487200	-1.94278700	1.86011400
C	-2.47487800	1.57914300	0.38225400
C	-0.52810000	-2.61355800	0.47868100
C	-2.20986000	-2.02681200	-1.36486700
C	-1.69222500	1.45562400	1.68755700
H	-2.35213000	1.05198000	2.46756600
H	-1.33071300	2.43267700	2.02206200
C	-2.71323700	-1.58583600	1.12934600
Mn	1.40827000	1.13596300	0.02117200
S	0.28020200	-0.27743800	-1.54763100
O	2.35146800	-0.61885700	0.49869900
O	0.05745400	3.61439700	-0.86430200
N	3.24405900	-2.62705700	-0.24526500
O	2.72375500	2.62901900	2.22548200
O	3.55496300	1.94279600	-1.85996200
C	2.47477000	-1.57913000	-0.38243600
C	0.52821900	2.61367300	-0.47849700
C	2.20999100	2.02662300	1.36496600
C	1.69209100	-1.45546800	-1.68771300
H	2.35197800	-1.05173700	-2.46769400
H	1.33056800	-2.43248400	-2.02231600
C	2.71328000	1.58582200	-1.12925900
C	4.02519300	-2.67111400	0.99666600
H	3.38207400	-2.75949300	1.88797200
H	4.68725600	-3.54531500	0.96961700
H	4.64504300	-1.77035700	1.13816700
C	-4.02528200	2.67097600	-0.99699000
H	-4.64515800	1.77021800	-1.13837200
H	-4.68731900	3.54520100	-0.97006100
H	-3.38215800	2.75921700	-1.88830500

1 3 S 20 0.1

- **[2^{Me}–2H^+]$^{2-}$** O-bound Mn—O bond cleavage optimization:

-2 1

Mn	-3.14376000	2.23538701	0.00000000
S	-3.48697100	0.29128101	1.19443500
O	-1.10650300	-1.20681799	-0.62650800
O	-2.94980200	4.12018101	2.25154700
N	-1.19707100	-2.85425599	1.03737700
O	-2.99150000	4.43690101	-1.99221800
O	-0.18289500	2.06826301	-0.13059400
C	-1.37888900	-1.63087399	0.53911600
C	-3.01608100	3.37700901	1.35124700
C	-3.07368100	3.56293701	-1.22197300
C	-1.91955600	-0.63695299	1.58248800
H	-1.16164800	0.12914701	1.77827800
H	-2.14105000	-1.16258699	2.51589100
C	-1.34759800	2.05335501	-0.08895700
Mn	-4.57432400	0.13769101	-0.95761800
S	-5.47400800	2.22266101	-0.26187300
O	-6.17590800	-0.63738799	0.05787200
O	-2.48126600	0.87643301	-2.93598100
N	-8.06950100	-0.24679699	1.34080200
O	-3.88541500	-2.67491899	-1.62404000
O	-6.26769600	0.27734101	-3.38615600
C	-6.93119100	0.11956301	0.81856800
C	-3.20725600	0.69304501	-2.03412700
C	-4.07751200	-1.57164799	-1.31834300
C	-6.41578300	1.50729401	1.16739500
H	-7.23578500	2.19019001	1.40286400
H	-5.73739400	1.44400101	2.02450900
C	-5.61474600	0.22630301	-2.42279000
C	-8.49524900	-1.61793899	1.03255100
H	-7.80860000	-2.36986099	1.45369400
H	-9.48925100	-1.78527399	1.46379000
H	-8.54979700	-1.80356699	-0.05207200
C	-0.56655300	-3.78033299	0.09033200
H	0.43574400	-3.44214899	-0.23037400
H	-0.45362200	-4.76256199	0.56860100
H	-1.15782200	-3.91470199	-0.83183900
